# Ad-based social media interventions increase belief accuracy and generate pro-social opinions among non-news readers

**DOI:** 10.1371/journal.pone.0352588

**Published:** 2026-06-29

**Authors:** Erin Wertz, Maria Babińska, Dominik Batorski, Debra Louison-Lavoy, Nicholas Blazer, Nancy S. Noble, Magdalena Wojcieszak

**Affiliations:** 1 Center for Excellence in Social Science, University of Warsaw, Warsaw, Poland; 2 Center for Social and Cultural Psychology, Université Libre de Bruxelles, Brussels, Belgium; 3 Interdisciplinary Centre for Mathematical and Computational Modelling, University of Warsaw, Warsaw, Poland; 4 Reality Team, New York City, New York, United States of America; 5 Department of Communication, University of California, Davis, California, United States of America; Toulouse Business School: TBS Education, SPAIN

## Abstract

Democratic challenges are often attributed to the spread of misleading, untrustworthy, or biased information, leading scholars to focus on minimizing exposure to such “bad” content online. Instead, we introduce a scalable intervention to put factual and verified public affairs information in users’ social media feeds to make them better informed and more resilient to various online threats. We conducted 48 field quasi-experiments using Instagram ads targeting news non-users to enhance their belief accuracy, democratic attitudes, and behavioral intentions related to climate change, COVID-19 vaccines, media literacy, and election integrity. The treatment videos reached 2,496,878 Instagram accounts, 690,470 users watched at least 50% of the video, and 40,584 of those users completed post-test assessment. The intervention was effective: 46 out of 48 of the quasi-experiments had positive effect sizes and 40 out of 48 achieved statistical significance. The intervention predicted not only belief accuracy but also attitudes, media literacy, and — to some extent — behavioral intentions related to vaccination. These patterns emerged across topics, did not dissipate with time (two of three climate change quasi-experiments show continued effects), and were not contingent on persuasive appeals and format features presented in the ads.

## Introduction

The United States and other countries face challenges to election integrity, democratic governance, public health, and the politicization of science [1–3]. Social media platforms, and the spread of “bad” content in particular, are often seen as culprits driving these problems [e.g., 1,4–7]. Accordingly, researchers examine how to flag, recognize, or decrease engagement with misinformative, untrustworthy, or biased information online. However, exposure to and engagement with such “bad” content is incredibly low and highly concentrated among a small subset of social media users [8–10]. That is, the core problem may be less that people consume “bad” political content than that many do not consume “good” public affairs information at all, as we note below. In other words, pressing democratic challenges are also and in large part driven by declining political knowledge, growing civic disengagement, and decreasing trust in public institutions among the electorate [11–15]. Yet, we lack versatile interventions that can be deployed online to promote exposure to factual and verified content toward making citizens better informed and more resilient.

In this project, we offer and test a scalable intervention that capitalizes on a readily available affordance of social media — targeted ads — to put factual and verified information on topics of civic importance in users’ feeds. Using the same infrastructure corporations use to sell products, we target news non-users with short video ads to enhance media literacy, belief accuracy, pro-social attitudes, and behavioral intentions.

In so doing, we hope to expand extant work in three ways. For one, we shift focus from minimizing “bad” information to increasing exposure to “good” information. As aforementioned, contemporary democratic challenges may be driven in large part by low exposure to verified public affairs information [16–18], not necessarily or primarily by misinformative, biased, or otherwise problematic content. News makes up only 1.4% of Facebook’s News Feed [19,20] and less than 0.5% on TikTok [21], and most users do not follow any politicians, journalists, or news organizations [21,22]. Instead, most content consumed on platforms is memes, pop-culture, humor, and viral music, dance or lip-sync performances [19], and platform algorithms actively redirect users away from news and toward entertainment [23]. This leads to uninformed and disengaged citizens who are susceptible to misinformative, populist, or hyper-partisan rhetoric [15,24–26]. In contrast, consumption of public affairs information increases knowledge, efficacy, attitude stability, and individual ability to discern true from false information [15,27–31]. Accordingly, our intervention makes factual, verified, and corrective messages readily available and easily accessible in users’ feeds to guarantee at least minimal exposure to content on important public issues. In short, we offer a scalable means of addressing the absence of “good” information.

Second, this shift in focus from minimizing “bad” content to enhancing exposure to accurate and verified information helps circumvent issues with existing interventions in this space. Approaches that alter users’ feeds, such as downranking like-minded sources [32], reranking to decrease the visibility of problematic content [33,34], or — similar to our work — increasing recommendations to diverse news and political content [35–37] can be implemented only by the platforms themselves or necessitate software extensions that consenting participants must install. Other approaches related to misinformation and digital literacy face other limitations. For instance, although fact-checking reduces misperceptions in experiments in the short-term [38–40], only a small fraction of misinformation consumers ever encounter related fact-checks in the real-world [41]. More problematically, fact checking is not effective if misbeliefs align with one’s ideology or elite cues [42], it rarely shifts behaviors [43,44], and generates lingering effects on related attitudes [45]. Other approaches related to misinformation and digital literacy face other limitations. For instance, interventions such as friction-based design tweaks or accuracy nudges [46,47] can reduce misinformation sharing and encourage reflective engagement. Yet, these interventions reduce sharing indiscriminately — suppressing both false and true information [48] — and their real-world impact is limited because most users simply do not share political or news content [22,49]) Furthermore, strategies such as digital literacy or inoculation games help users recognize manipulation techniques and anticipate misinformation [50,51], but they also increase skepticism [52–55] and lower trust in credible journalism [48,56,57]. Thus, despite the theoretical promise of many interventions, many are difficult to scale, unlikely to reach their target audience, or risk inadvertent harm. Our intervention influences information available to social media users without requiring explicit platform cooperation and is unlikely to entail the side effects generated by other approaches.

Third, we focus specifically on the large population of news avoiders and news non-users; those who do not consume news and public affairs [see 58]. This group is not only large but also theoretically and practically important [see 59]. Around half (47%) of citizens internationally do not consume news, the proportion of news non-users has increased by over 10% since 2016 in the U.S. [60] as news avoidance has also grown, with 39% of respondents internationally stating they sometimes or often avoid the news [61]. Those citizens are politically disinterested, largely disengaged, and unlikely to vote [60,62,63], report lower levels of political knowledge, are more likely to answer “not sure” to questions about current events, and more likely to have been exposed to false claims and conspiracy theories than those who consume news [63]. In short, this group can be easily influenced by misinformation, hyper-partisan rhetoric, or irrelevant or manipulative cues in the environment [24,26]. Targeting these citizens to make them better informed and more resilient is therefore important [see 64]. After all, exposure to verified and factual public affairs information increases knowledge [12,15,31], opinion stability, political efficacy, tolerance, and the acceptance of democratic norms [29,30], and individual ability to discern true from false news stories [27,28]. We follow other recent efforts that link the work on news non-use to actionable pro-social interventions [e.g., 17,57,65] and design an intervention that not only offers easily digestible information but also empowers a consequential group of users to recognize misinformation and manipulative techniques, generating over-time outcomes that enhance democracy.

More specifically, we developed a versatile method to approximate randomized control trials, which leverages publicly available features of the Instagram Ad Manager to test the impact of short video messages on non-news readers’ knowledge and attitudes – all within their ordinary platform experience. We relied on Instagram ads to target the users who do not follow, visit, or engage with any news organizations — whether via major news networks, prominent partisan outlets, or local or national newspapers — across Facebook, Instagram, other Meta Products, and off-platforms, such as on non-Facebook websites or apps. The news non-users in various U.S. states were assigned to treatment or control groups using the users’ profile birth month to approximate random assignment, as detailed below. We ensured that each group had a large enough population, roughly 20,000 members, to ascertain enough users who viewed the ads and completed the post-test assessments. This unique method was applied to a series of 48 field quasi-experiments deployed on Instagram, a platform used by approximately half of American adults and over three quarters of those aged 18–29 [66]. Supplementary Materials, S1 Section 1 in S1 Text, details the partnership between academics and a non-profit that ran those ads.

For each quasi-experiment, we created short videos with simple verified information (see S1, Section 2.3 for details in S1 Text). The treatment videos had 4,188,616 impressions, reached 2,496,878 Instagram accounts, and 690,470 users watched at least 50% of the video. The post-test polls were completed by 40,584 of those users. The quasi-experiments focused on climate change (28 quasi-experiments), COVID-19 vaccines (5 quasi-experiments), election integrity (11 quasi-experiments), and media literacy (4 quasi-experiments). S1 Section 3 in S1 Text presents additional details on the topics and the screenshots of the treatments. To understand what ad features may prove most effective, we categorized the specific persuasive appeals used into five types and compared the efficacy of each: source credibility, social norms, benefits, logical inference, and “other.” We also evaluated whether differences in format pertaining to the inclusion and type of visuals in the ads influenced our results. S1 Section 4 in S1 Text details the ads’ appeals and format features in more detail.

We use those data to address five progressively specific questions. Are targeted ad-based interventions effective in generating the intended pro-social outcomes among news non-users (RQ1)? Are these interventions differently effective for belief accuracy, attitudes, versus behavioral intentions (RQ2)? Are these interventions applicable across different divisive topics or do they backfire (RQ3)? Are these interventions effective in the long term (RQ4)? Because nine of our quasi-experiments on climate change had delayed post-tests, our project is uniquely suited to estimating these patterns over time. Do certain ad content features, both in terms of persuasive appeals and format features, influence intervention effectiveness (RQ5)?

We offer five key findings. First, our ad-based intervention was effective: the majority of the quasi-experiments yielded positive outcomes (46 out of 48) and most of them were statistically significant (40 out of 48). On average, the treatment group was 2.80 times more likely to respond to the post-test assessment poll in accordance with the information in the treatment ad than the control. Second, the intervention not only positively predicted belief accuracy but also statistically significantly predicted more pro-social attitudes, with mixed patterns for behavioral intentions. Third, the pattern of positive outcomes were similar across the four topics. For instance, those exposed to our ads on climate change were 2.46 times more likely than the control to hold accurate beliefs on the issue, and those users who watched the treatment videos debunking the alleged 2020 election fraud were 4.62 times more likely than the control to agree that the elections were secure. In short, the intervention did not backfire even though the tested topics represent contentious topics. Further, these statistically significant patterns did not dissipate with time. Even when the assessments for our three climate change quasi-experiments were done 6, 80, 154 and 229 days after actual exposure, we still find significant results. Lastly, we show that the ads were equally effective across the tested theoretical appeals and format features.

Before presenting the data and the results, we acknowledge two core limitations of these studies. First, Meta Ads Manager only offers aggregate data, and so we have no individual-level information on the participants. As such, we cannot control for individual-level covariates or assess pre-to-post individual change, instead showing aggregate differences between the users in the treatment and the control groups (see S1 Section 2 and S1 Section 6.1 in S1 Text). Second, due to the nature of treatment implementation in quasi-experiments in the field, we had no control over who saw the ads and watched the treatment videos. For instance, not everyone who saw the treatment ads responded to the short in-feed post-test assessments, which means that we may have different opt-in biases between control, treatment, and those who respond to the post-test. Because we only have aggregate information on the users who saw the treatments and responded to the in-feed surveys with the post-test assessment, these biases are difficult to quantify. To shed some light on self-selection, we retrieve data on users’ gender and age from Meta’s Ads Manager (no information is available from Meta on other user characteristics), and mention these analyses at the start of the Results section. In short, because there are no pre-intervention measures and the treatment and control groups could potentially contain different subgroups, we cannot make strong causal claims. We acknowledge these limitations in more detail in the discussion.

## Data and methods

**Fig 1:**
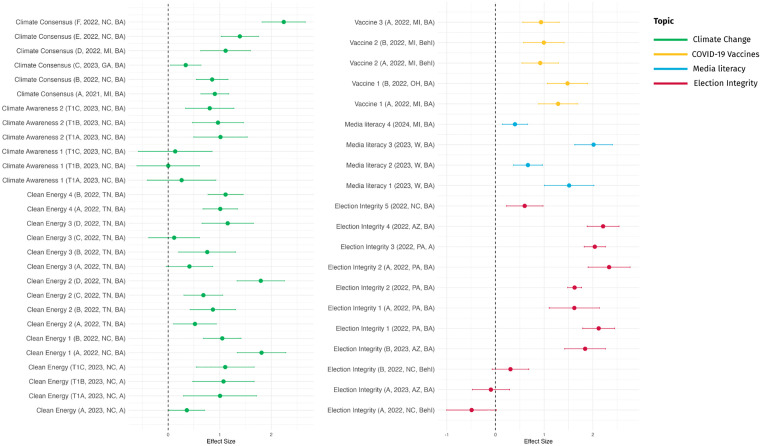
Forest Plot representing effect sizes of the individual quasi-experiments. *Note.* Colors represent the topics of the treatments. The labels indicate the topic and version of the post-test assessment. Letters are used when the same post-test assessment was used in more than one quasi-experiment. T1 to T4 indicates quasi-experiments with delayed post-test. Parentheses indicate the year the quasi-experiment was conducted, the state in which the quasi-experiment took place, and the outcome (BA for belief accuracy, A for attitude, and BehI for Behavioral Intention). Error bars represent standard errors (SE).

We conducted 48 quasi-experiments, each with a treatment and control group, with nine quasi-experiments including four measurements taken at different time points. The methodology follows Randomized Control Trials on Instagram, a method developed by Reality Team, called the Instagram Video Poll (IVP) method. Each quasi-experiment focused on a specific geographic location and topic. Audiences were divided into control and treatment groups. Each quasi-experiment consisted of a treatment campaign with a brief video (i.e., treatment) shown to the treatment audience, and a post-test assessment campaign, shown to both the treatment and the control audiences, consisting of an interactive Instagram story post with a single, binary poll question. In some cases, several questions of both groups were asked (S1 Fig 1 in S1 Text shows the design of the Instagram Video Poll method). In general, each quasi-experiment was run until there were roughly 200 respondents in each group. The exact number varies due to the granularity of ad manager campaign controls. S1 Table 1 in S1 Text details sample sizes of the post-test assessment polls.

### Audiences and targeting

The quasi-experiments aimed to reach an audience that we call news non-users. Those are individuals who are not active consumers of news and public affairs information and more likely to avoid news actively or passively. To reach this group on Instagram, the targeting criteria in 2021 were to exclude users who had followed, visited, and/or interacted with specific news organizations on Instagram, Facebook, and other Meta Products, and who engaged with news organizations off-platforms, such as on non-Facebook websites or apps. Specifically, in 2021, we excluded those who follow, visit, and/or interact with the major news networks in the U.S., the partisan TV channels, newspapers, and podcasters and talk radio. These included ABC News, ABC World News Tonight, Ben Shapiro, CBS Evening News, CBS News, CNN, Fox News Channel, Mark Levin, Matt Walsh, MSNBC, NBC News, NBC Nightly News, Sean Hannity, The New York Times, The New York Times Magazine, The Washington Post, TheBlaze, and TheBlaze Radio Network. In January 2022, Meta replaced the specific outlets with general news categories. Given that Meta’s targeting rules changed, we adjusted the targeting and excluded users who followed, visited, and/or interacted with accounts categorized as All-news radio (radio), Breaking news (news service), News broadcasting (news service), News magazine (publication), News media (news service), Online newspaper (publication), Newspapers (publications).

### Fielding the quasi-experiments

The quasi-experiments were run entirely within the Instagram user experience, using paid ads and various features of the Meta Ad Manager, and constructed using publicly available ad features. We first used geographic targeting to restrict our campaigns to a single state. We then refined the targeting by excluding Instagram accounts that engaged with news outlets, as described above. We used the user profile birth month to approximate the random assignment of users to treatment or control groups. We assigned two non-contiguous birth months for treatment and two other, non-contiguous birth months for control. We ensured that each group had a large enough population (roughly 20,000 members) to generate sufficient levels of engagement with the treatment ads and the post-test assessment polls. This allowed us to control audience size and to run several quasi-experiments in each geography with fresh audiences that had not been exposed to prior treatments. S1 Table 1 in S1 Text offers details on each quasi-experiment, along with the dates, location, and sample sizes in both the treatment and control groups.

### Ethics

Per Meta’s privacy policy, https://www.facebook.com/privacy/policy/, Meta provides advertisers with reports that include the number, general demographics and interests of people who see and engage with their ads. Meta does not share information with advertisers that can be used to identify individuals. Because the quasi-experiments were all conducted on Meta, we have no individual-level information on the participants, who remain fully anonymous, and from whom no personal, sensitive, or other human subjects data are collected, and from whom it would not have been possible to solicit informed consent. The project was reviewed by the UC Davis Institutional Review Board, which determined that the activities do not meet the definition of human research (IRB ID 2219323-1). In sum, only aggregated data are analyzed, there is no deception, and the societal benefits of the studies far outweigh any potential harms to participants, which would not be larger than what individuals experience in their ordinary life.

### Treatment

In each quasi-experiment, we created a 10–15 second video with simple, fact-based and verified information on four topics: climate change, COVID-19 vaccines, electoral integrity, and media literacy. The climate change treatments comprised three message types: (1) information on the scientific consensus that climate change is real and caused by humans, (2) evidence that climate change is solvable, or (3) evidence pertaining to the economic benefits of climate solutions. The COVID-19 messages split between two types: (1) informing users about the low relative risk of vaccine side effects and (2) noting the percentage of medical doctors who were vaccinated at the time of the quasi-experiment. The election integrity treatments were split between (1) a common format providing quotes from public figures across the political spectrum, (2) noting the number of people who work and volunteer in election day in participants’ states, or (3) highlighting the use of local schools and churches as election polling places in those communities to emphasize the role of trusted community institutions in vote counting. Lastly, the media literacy messages warned participants about how ad hominem attacks, false dichotomies and loaded arguments are used for manipulation (see details and examples in S1 Section 3 in S1 Text). The videos primarily conveyed information via text, and did not have any accompanying sound, though many included visual aids of a variety of types (for details, see S1 Section 3.2 in S1 Text).

The treatment group was targeted with that video. The treatment videos had 4,188,616 impressions and reached 2,496,878 Instagram accounts. Given the nature of social media advertisements, i.e., users may ignore the ads or scroll past without noticing, we cannot guarantee that everyone in the audience saw the video, but we could create a list of anonymous account identifiers for those users who did watch the video. Those who watched at least 50% of the video were added to our “watched” group, which comprised 690,470 users. To ascertain equal treatment among the users in the treatment group, once someone was added to the “watched” group, they were removed from the target audience so they would not see the treatment video in their feed again. As aforementioned, although we have a list of accounts that had watched the video, we have no information about those accounts other than that they viewed the video. Again, we do not (and cannot) get any individual (e.g., socio-demographic) information about the users and — as such — our data are aggregated to the level of the quasi-experiments and analyzed as such.

To better understand differences between individual treatments, each treatment was reviewed and assigned to one of five categories based on the most prevalent persuasive appeals used within the treatment ad. These were (a) source credibility, consisting of appeals to the expertise, such as scientists, or to trustworthiness of sources, such as citing officials for issues of election integrity [67]; (b) social norms, consisting of either injunctive norm, what people believe others expect of them, or descriptive norms, how people believe others actually behave [68]; (c) benefits, consisting of both self-benefits which inform participants of what they stand to gain, and other-benefits which suggest that participants’ communities would benefit [69]; (d) logical inference which used numerical or other deductive reasoning to convince participants of basic facts about the topics; and (e) other, those appeals that were either unclear or did not fit with the categories above. For more details, as well as exact definitions used for coding, see S1 Section 3 in S1 Text 1.

### Post-test assessments

Once we had enough people in the “watched” group – a minimum of 10,000 users who watched the treatment video at least 50% of the way through, we began post-test assessment polls targeting this “watched” group and the control group, which was not exposed to any treatment video. The assessment polls were Instagram story ads that contained a “poll sticker.” A poll sticker is an interactive feature of Instagram stories that allows us to ask a user a single, binary (yes/no or true/false) question without asking them to leave their feed (see S1 Section 5 in S1 Text for all outcome measures). Most of our poll stickers evaluated whether the intervention’s message was reflected in participants’ belief accuracy through their ability to either identify whether a given statement was true or false, or to choose the correct statement out of a pair containing one true statement and one false, e.g., “More than 20,000 Tennesseans work for Electric Vehicle manufacturers making more than $20/hr,” “The climate is now changing much faster than in prehistoric times.” A second set of poll stickers measured participants’ attitudes toward the tested issues, e.g., “Should the government invest in renewable energy?” or “Do you trust election workers?” Lastly, a few quasi-experiments tested behavioral intentions, asking the users if they intend to get vaccinated against COVID-19 or to vote in the 2022 midterm elections, though several in the last category suffered from unclear or incorrect wording.

We ran each post-test assessment poll until we got a minimum of 200 answers each in the treatment and control groups. In a few trials, where the treatment audiences were smaller than normal, we had fewer than 200 responses (see S1 Table 1 in S1 Text). In total, the post-test polls were completed by 40,584 of those users. We did not have precise control over how soon after seeing the treatment video a user saw the assessment poll. For most of the quasi-experiments it was a minimum of 24 hours and a maximum of 96 hours after the treatment. For the quasi-experiments measuring the results over-time, the assessment began six days after the start of the treatment ad and was repeated three times (after 80, 154, and 229 days; see S1 Section 6.5 in S1 Text). As for the treatment exposure, as noted, we do not and cannot get any information about which specific accounts answered the poll, but only the total number of responses and how many users selected each answer option.

## Results

As aforementioned, we had no control over who saw the ads and watched the treatment videos, and no access to individual-level data but only aggregate information on the users who saw the treatments and responded to the in-feed surveys with the post-test assessment. Yet, we retrieved data on users’ gender and age from Meta’s Ads Manager, which provides statistics on reach, number of impressions, clicks and ad post engagement by age group and gender. Thus, we first evaluate whether there were statistically significant differences in these demographic characteristics between the groups in terms of randomization and self-selection, as detailed in S1 Section 4 in S1 Text. The findings indicate that randomization was not effective on age and gender due to Meta’s ad delivery systems. Specifically, (a) the users reached by the treatment ads (i.e., videos to the treatment group) and the post-test assessment ads in the control and also (b) those reached by the post-test assessment ads in the treatment and control groups differ on gender and age. If Meta’s advertising algorithm had displayed ads entirely at random, we would expect no differences between the groups, and the detected differences show that the algorithm systematically delivers different ads to different users.

We also find self-selection to watching the treatment video; those who were shown these videos consistently differ from those who watched at least 50% of the video in terms of age, and in half of the cases, also in terms of gender (see Column 3 Tables 2 and 3 in S1 Section 4 in S1 Text). This self-selection, in our view, is fully expected; unlike in controlled experiments, in naturalistic settings exposure is not “forced” and people can choose to watch the treatment or opt out [see 70,71]. More importantly, we also show that self-selection bias is *lower* for the post-assessment ad response. Those who received the post-test assessment ad and those who responded do not significantly differ on gender in 92% of the quasi-experiments and on age in 66% of the quasi-experiments. Lastly, we compare the users in control and treatment who responded to the post-assessment ads. Here, we find no statistically significant differences in gender between the treatment and control groups in 81% of the quasi-experiments. However, for age, the differences are significant in 91% of quasi-experiments.

These findings indicate that despite ineffective randomization and the expected self-selection to watching the treatment video, those who completed the post-assessment ads in the treatment and control were similar on gender, but less so on age. Although limited, these analyses at least partly suggest that the detected patterns are not solely attributable to self-selection on these two factors. We acknowledge that Meta does not offer any additional data on users, which could facilitate comparisons on potentially more consequential variables, such as partisanship, policy attitudes, perceived issue salience, and that we cannot therefore examine whether treatment and control were similar to each other with balance tests. In general, it is difficult to ascertain randomization and minimize opt-in biases in field experiments, and our project is limited in this regard. Nevertheless, the at-scale evidence offered below — although not free from limitations — may be valuable to scholars and practitioners.

### Overall intervention effects

To determine the overall effectiveness of the intervention on all outcomes (RQ1), we conducted a meta-analysis. Given that each quasi-experiment included one control and one treatment group with binary outcomes, we applied a random-effects logistic regression model (see S1 Section 6.1 in S1 Text for model description). This approach allowed us to estimate pooled effect sizes while accounting for heterogeneity across studies. We first analyze data from all 48 studies to estimate the influence of our treatment ads (we include only the first assessments from the three over-time quasi-experiments). Fig 1 plots the results (i.e., log odds ratios), broken down by the topic and S1 Section 6.2 in S1 Text details the model results. Overall, the majority of the effects (46 out of 48) were positive and 40 out of 48 were statistically significant (neither negative result was significant). Their strength varied across the studies, ranging from −0.49 to 2.33 (in log odds ratios). On average, the log odds ratio effect size was μ = 1.04 (z = 10.72, 95% CI [0.85, 1.22]), which means that people who watched the treatment ads were 2.86 more likely to answer in line with the messages presented than the control. We also note high heterogeneity in this analysis (I^2^ = 91.7%), indicating that there are substantial differences by topic and outcomes, which we also explore.

### Intervention effect sizes by outcome

**Fig 2:**
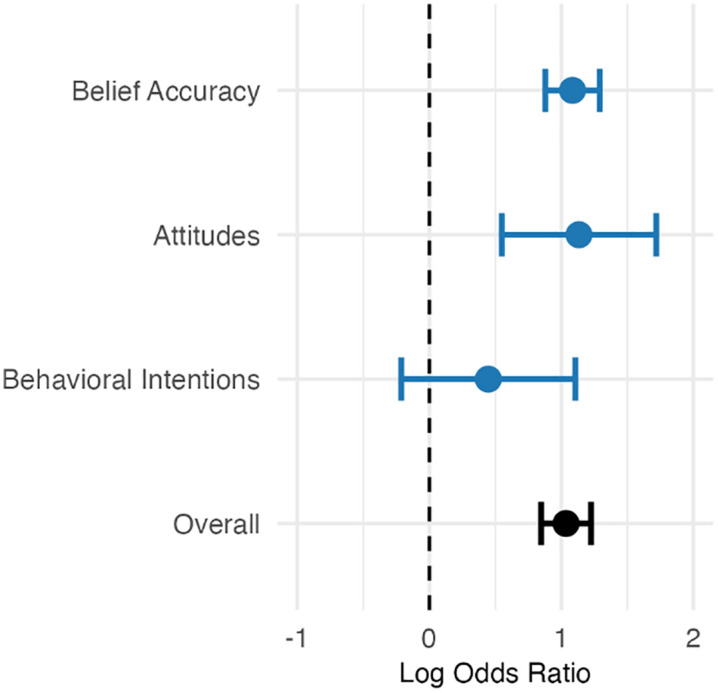
Average effect sizes of treatments on the four types of outcomes. *Note.* Error bars represent 95% CI. We aggregate quasi-experiments that assessed the four kinds of outcomes. Fig 1 shows the effect sizes for each quasi-experiment individually and S1 Table 6 in S1 Text shows the wording of the outcomes.

To examine whether these patterns depend on the type of outcome, we compared the effect sizes across belief accuracy, attitudes and behavioral intentions (RQ2). Although most of the quasi-experiments assessed belief accuracy, three focused on attitudes and four on self-reported behavioral intentions. Fig 2 plots the aggregated effect sizes, S1 Table 6 in S1 Text shows the wording of the outcomes, and S1 Section 6.4 in S1 Text details the analyses. We found no differences in the average effect sizes (Q(2) = 3.39, *p* = .18), suggesting that the differences in effect sizes are not due to differences between the outcomes. Specifically, on average, users in the treatment group were 2.94 times more likely than the control to hold accurate beliefs on the tested topics (μ = 1.08, 95% CI [.88, 1.29], z = 10.32, *p* < 0.001), such as recognizing true statements regarding the speed of climate change or the legitimacy of the 2020 presidential election, as well as correctly detecting manipulation, misinformation, or clickbait. The treated users were also 3 times more likely than the control to report attitudes consistent with the ad, such as supporting investments in green energy or reporting trust in election workers (μ = 1.13, 95% CI [0.55, 1.72], z = 3.80 *p* < 0.001). However, the treatment had no significant effects on behavioral intentions in the aggregate, even though several individual effects on the intentions to get a COVID-19 vaccine were statistically significant.

### Intervention effect sizes by topic

To test if the intervention’s effects varied by topic (RQ3), we compared the effect sizes of the treatments for climate change, COVID-19 vaccine, media literacy, and election integrity (RQ3). The average estimated effect size was statistically significant within the four topics (*p* < 0.001), with no significant differences between them (Q(3) = 3.14, *p* = .37). This indicates that the treatment ads were equally effective regardless of the issue. S1 Section 6.3 in S1 Text presents the details of the analyses.

Descriptively, as shown in Fig 1, for climate change, all the effects were positive and 82% (n = 23) were statistically significant. On average, users who saw the treatment ads on this issue were 2.46 times more likely to answer in accordance with the intention of the ads. The observed log odds ratios, which measure the effect sizes, ranged from 0.002 to 2.24, with the average effect size, calculated using a random-effects model, being μ = 0.90 (95% CI [0.71, 1.09], z = 9.19, *p* < 0.001). There were 3 sub-topics pertaining to climate change. Sixteen ads covered clean energy; people who saw them were, on average, 2.5 times more likely to indicate that green energy generates economic benefits (μ = 0.92, 95% CI [0.69, 1.15], *z* = 7.90, *p* < 0.001). Six ads addressed scientific consensus on climate change; the treatment group was 3.09 times more likely than the control to indicate that “nearly all scientists agree it’s real, and we cause it” (μ = 1.13, 95% CI [0.62, 1.64], *z* = 4.34, *p* < 0.001). Finally, six ads pertained to climate change awareness; the treatment group was 1.79 times more likely to hold more accurate beliefs than the control (μ = 0.58, 95% CI [0.22, 0.93], *z* = 3.18, *p* < 0.001). For instance, 61% of the treatment group correctly responded that the statement “The climate is now changing much faster than in prehistoric times” is true, compared to only 37% in the control (χ²₁ = 14.25, *p* < .001).

With regard to COVID-19 vaccines, the treatment ads had positive and statistically significant effects. The treatment group was, on average, 3.06 times more likely than the control to indicate that vaccines are safe, with the effect sizes ranging from 0.92 to 1.48 (μ = 1.12, 95% CI [0.90, 1.33], z = 10.16, *p* < 0.001). For example, 63% of the treatment group indicated correctly that the statement “Almost all U.S. medical doctors are fully vaccinated against COVID-19” is true, compared to 33% of the control group (χ²₁ = 50.51, *p* < .001) Similarly, 59% of users in the treatment group correctly indicated that “You’re much more likely to get struck by lightning than a serious side effect from a COVID vaccine,” as opposed to 36% in the control (χ²₁ = 23.61, *p* < .001).

Similarly, all four quasi-experiments on media literacy significantly increased users’ ability to spot clickbait and manipulative content. The effect sizes ranged from 0.40 to 2.02, with an average of 1.14 (95% CI [0.39, 1.87], z = 3.01, *p* < 0.001). The treatment group was 3.13 times more likely to recognize such content compared to the control. For instance, 52.94% in the treatment group indicated that the statement “You’re wrong because you’re too dumb to understand” was manipulative, as compared to 13.03% in the control (χ²₂ = 117.16, *p* < .001).

Lastly, 82% of the effects pertaining to election integrity were positive and the majority (89%) were statistically significant. The treatment group was on average 3.42 times more likely to respond in accordance with the intention of the ads than the control group (μ = 1.29, 95% CI [0.70, 1.88], z = 4.26, *p* < 0.001), with the effect sizes ranging from −0.73 to 3.31. For instance, 74% of the treatment group saw the 2020 Election as legitimate, compared to 21% in the control group (χ^2^ = 125.56, *p* < 0.001). Similarly, 62% of the treatment group correctly indicated that the evidence shows that the 2020 election was secure as opposed to 17% in the control group (χ^2^ = 184.82, *p* < 0.001). We found significant heterogeneity in the effect sizes for three of the topics (climate change I² = 84.1%, Q(23) = 128.49; election integrity I² = 97.5%, Q(10) = 261.27, media literacy I² = 94.6%, Q(3) = 53.90), indicating that the effects vary by untested factors, for which we cannot account or control due to the aggregate nature of the data. For COVID-19 vaccines quasi-experiments, we find acceptable homogeneity (Q(4) = 5.95, p = 0.20).

### Intervention effect sizes over-time

**Fig 3:**
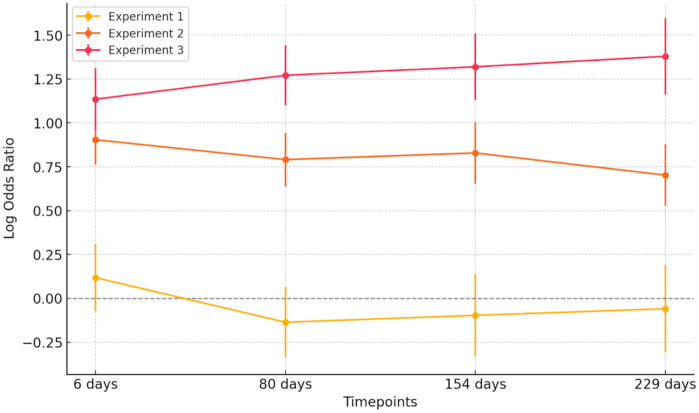
Effect sizes of 3 quasi-experiments across 4 measurements. Note: Quasi-experiment 1 post-test assessment: “The climate is changing at exactly the pace that we are burning fossil fuels;” Quasi-experiment 2 post-test assessment “The climate is now changing much faster than in prehistoric times;” Quasi-experiment 3 post-test assessment “Should the government invest in clean energy solutions?”.

Nine individual quasi-experiments on climate change included delayed post-test measurement at 6, 80, 154, and 229 days after the treatment launch, allowing us to test the over-time effects. Three quasi-experiments had the same treatments and were run in three geographical areas. As the results did not differ by area (see S1 Section 6.5 in S1 Text), we aggregate them and show the overall over-time effects of the three treatments. Fig 3, which plots the effects, shows statistically significant over-time effects in two quasi-experiments. The treatment group was 2.25 times more likely than the control to hold accurate beliefs on the speed of climate change at all the time points (Quasi-experiment 2: μ = 0.82, 95% CI [0.66, 0.97], z = 10.20, *p* < 0.001). Throughout the measurements, the treatment group was also, on average, 3.5 times more likely to support investment in green energy (Quasi-experiment 3: μ = 1.27, 95% CI [1.08, 1.45], z = 13.57, *p* < 0.001). The first set of quasi-experiments did not yield significant results, likely due to a flawed wording of the outcome measure (Quasi-experiment 1; see note to Fig 3).

### Intervention effect sizes by intervention features

**Fig 4:**
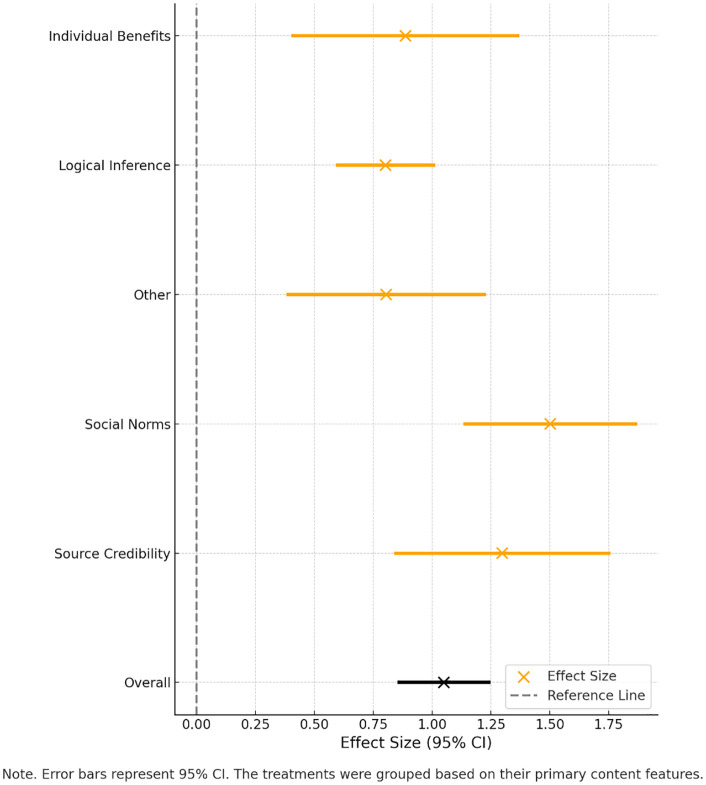
Average effect sizes of treatments in different persuasive strategies. *Note.* Error bars represent 95% CI. We aggregate the treatments that utilize the same primary persuasive strategy.

To offer insights into ad features that could drive the detected results (RQ5), we compared the effects of all advertisements by our coded persuasive appeals (source credibility, social norms, individual benefits, logical inference and other). Fig 4 shows the effects of treatment by persuasive appeal. In order to examine potential sources of variability in effect sizes, we conducted a random-effects meta-regression analysis using REML estimation. The meta-regression model revealed a significant overall effect of the predictors on the effect sizes (Wald Chi-square = 10.285, df = 4, p = .036). However, when examining individual predictors, none demonstrated a statistically significant effect. The predictor ‘Logical Inference’ approached significance (Estimate = −0.529, p = .058), indicating a potential reduction in effect size for this category. Other predictors, including ‘Individual Benefits’ (Estimate = −0.412, p = .204), ‘Other’ (Estimate = −0.493, p = .069), and ‘Social Norms’ (Estimate = 0.206, p = .475), did not significantly explain variability in effect sizes. However, a test of subgroup homogeneity showed a statistically significant (p = .009) difference in effect sizes between appeals. Further analysis indicated that the effect size for Logical Inference was 0.802 (95% CI [0.597, 1.006]). Comparisons of Logical Inference with other subgroups found that logical inference interventions had significantly smaller effects than both Social Norms (95% CI [1.141, 1.864]) and Source Credibility (95% CI [0.846, 1.751]), though not other interventions. These findings suggest that Logical Inference is less effective than appeals to Social Norms and Source Credibility, but not statistically significantly different from other content features.

Furthermore, we accounted for specific format features. Whereas all ads were short videos without sound, they varied in whether they included visual aids in addition to text as well as what form those images took. An additional analysis found that these format types did not have differential effects on the outcomes tested (see S1 Section 6.7 in S1 Text for full model specifications). Although richer media or images could improve ad effectiveness [72] or, conversely, distract the users [73], our informational ads were effective across these content features.

## Discussion

Many countries around the globe are facing crises of elections, democracy, health, and climate change, many of which are driven by polarization, radicalization, and mis- and disinformation. Many scholars study how to increase citizen resilience to these threats. This project proposed an efficient method to aid these efforts, advancing a versatile intervention that relies on social media ads targeting those users who do not follow, visit, or engage with news media. News non-users represent the majority of the American population [59] and are susceptible to misinformation, populism, and hyper-partisan rhetoric [15,26], making it pressing to target this group with factual and corrective information.

Our analysis across all 48 quasi-experiments shows that the intervention exerts substantially and significantly predicts the tested outcomes. This evidence expands past work, where interventions, such as minimizing exposure to like-minded sources on Facebook [32] or increasing recommendations to verified and diverse news on YouTube [37], have no effects on polarization, knowledge, or belief accuracy. Although we do not have individual-level data and, as such, could not measure our outcomes pre-treatment or test individual-level covariates, our results suggest that simple ad-based social media campaigns are correlated with — and may lead to — pro-social outcomes.

We also show that this intervention positively predicts not only belief accuracy but also attitudes, media literacy, and — to some extent — behavioral intentions related to vaccination (we emphasize, however, that behavioral intentions may not translate to actual behavior). These patterns, moreover, emerge across topics. Given that climate change, COVID-19 vaccines, and election integrity constitute salient issues in American politics, the fact that we observe no backfire effects is noteworthy. In fact, the most pronounced results (although not statistically significantly different from those on other topics) emerged for election integrity, an issue that was very divisive at the time of the quasi-experiments. Furthermore, two of our three climate change quasi-experiments found continued treatment effects on belief accuracy and attitudes, indicating that the differences between treatment and control group do not dissipate with time. However, because the delayed quasi-experiments were limited to climate change, we cannot conclude that similar over-time patterns occur across topics. Given the comparable effect sizes across issues, we suspect that these patterns would be similar for COVID-19, election integrity, and media literacy, yet future research is needed to test this. In short, watching short informational videos worked across topics and outcomes.

Lastly, we offer suggestive evidence that the various persuasive appeals and content features had a limited influence on the overall patterns detected in our studies. Logical inference appeals relying on deductive reasoning and statistics about issues had a smaller effect size than others, but this evidence was weak and somewhat inconsistent, while no evidence suggested any difference between appeals to source credibility, social norms or self- and other- benefits. Similarly, including images or graphics neither enhanced ad effectiveness [72] nor distracted from the information presented [73]. In short, the ads worked across the tested theoretical appeals and format richness. We encourage future work to isolate additional features, whether narratives, emotional appeals, gain versus loss frames, among others.

Our results are noteworthy as our ads competed with many other ads that the users see on Instagram and given that there was at least a 24-hour lag between treatment exposure and the in-feed post-assessment polls. Because most experiments isolate the treatments more neatly than our quasi-experiments did and tend to assess effects immediately after exposure, the detected mid- to long-term patterns are promising. To the extent that the intervention aimed to build long-term competences (e.g., recognizing bias or manipulation in media) and offered factual information on the tested issues, the detected patterns should continue (although only our climate change cases featured delayed post-tests).

Again, we acknowledge the major limitation of this project, which is that due to the nature of treatment implementation, we had no control over who saw the ads and watched the treatment videos. For instance, not everyone who saw the treatment ads responded to the short in-feed post-test assessments, which means that we may have different opt-in biases between control, treatment, and those who respond to the post-test. Because we do not have individual-level data but only aggregate information on the users who saw the treatments and responded to the in-feed surveys with the post-test assessment, these biases are difficult to quantify. The analyses, reported in S1 Section 4 in S1 Text, suggest that Meta’s ad manager did not distribute the ads at random and that there was expected self-selection to watching the treatment video, given that it was not a “forced exposure” experiment (S1 Section 4 in S1 Text). Nevertheless, those who completed the post-assessment ads in the treatment and control were similar on gender, but less so on age. Again, we cannot analyze randomization or self-selection across any other individual level variables, and it is therefore possible that the treatment could have driven certain types of people — such as those higher at baseline on post-test measures — to complete the post-test poll. Because we have very limited information about the participants in the study, we cannot robustly estimate the scale of these potential problems, nor do we have co-variates to help adjust the estimates or provide sensitivity analyses. Future work, which collects additional information about treatment assignment and participants, is needed to ascertain whether the treatment effects presented above are fairly close to the estimates that would be obtained from experiments with more controlled assignment to treatment and post-treatment measures. For now, we trust that the large-scale evidence presented above is suggestive of promising and relatively easily scalable pro-social interventions.

Furthermore, we note that the Instagram advertisement features we used to deploy post-tests limited us to using outcome measures consisting of single-question binary polls. Such measures are inherently limited in general and may have compromised our ability to clearly distinguish between, say, beliefs, attitudes, or information gain (e.g., our belief items may contain elements of normative attitudes). Additionally, in the case of belief accuracy, our measures consisted of factual endorsement items administered shortly after exposure, and we cannot ascertain if changes observed using these measures reflect short-term information uptake, accessibility, or demand characteristics rather than deeper or more durable belief change.

We also acknowledge that the detected effectiveness of our intervention could easily be weaponized for nefarious purposes by bad actors aiming to influence individual views and behaviors, or simply disseminate inaccurate information [4,5,7]. To minimize the influence exerted by such problematic efforts, improving users’ digital literacy skills is needed, and ads such as ours can be used toward these ends. Similarly, it is not clear whether similar patterns would be detected in cases of competitive or adversarial messaging. Our studies took place in largely non-confrontational informational contexts, where competing messages are not systematically introduced. Yet, the market-based advertising infrastructure we rely on is equally accessible to actors with competing or opposing agendas. As such, the detected results may be weaker or non-existent in competitive or adversarial advertising environments.

Moreover, our purposefully selected sample of news non-users may respond much more to treatments such as ours than highly partisan or polarized individuals. Because this group has weaker attitudes and partisan positions than those interested in news and current affairs, our target populations are more easily influenced [59,74]. Furthermore, this group could respond to competitive or adversarial messaging just as strongly as to our treatments. That said, we found promising patterns on belief accuracy and manipulation detection, which are especially important among this group and in situations, in which bad actors were to spread misinformation or engage in counter-messaging.

We also encourage future work to test whether our approach works across social media platforms and political contexts. Given the widespread nature of social media ads and the relative ease of audience targeting, we expect that our approach is applicable across platforms and that putting verified, factual, and corrective information in users’ feeds can generate similar outcomes in different countries and on different issues. In a similar vein, studies should compare the effects of similar social media field (quasi-)experiments to alternative delivery methods to show whether users react differently if they receive the ad in a lab or online. Lastly, research should test whether similar interventions can influence actual behaviors. Changes in awareness and attitudes are essential to subsequent behavior change [75] and changing attitudes indeed leads to small-to-medium effects on behavior change [76]. Germane here, some studies using social media ad campaigns to impact election-related behaviors find small positive effects on voter turnout [77], especially among sub-groups of voters [78–80]. Yet other work finds null effects in this context [81,82]. We note that our post-assessment questions about behavioral intentions generated the weakest results. Therefore, although theoretically behavioral intentions should correspond to behavior change, we cannot ascertain these actual behavioral outcomes.

Given that emotional outrage, low-quality content, and false and polarizing information, easily spread online, eroding public health, democratic values, and consensual governance [1], it is pressing to find ways to “promote the Internet’s potential to bolster rather than undermine democratic societies” [83, p. 1102]. We proposed a versatile approach to increase exposure to factual and corrective information and impart knowledge and skills among the consequential population of those who do not consume or engage with news. Given that those social media users are vulnerable to various information threats, it is crucial to empower them to be better informed and more media literate. This is crucial also because those users are not recommended news and quality public affairs information because platform algorithms, in their effort to maximize user engagement, keep recommending entertainment content over news and essentially shield users from public affairs information [23]. Thus, our effort also connects the growing literature on news avoiders and non-users to as-yet siloed off research on online prosocial interventions.

Ultimately, better informed and more digitally literate citizens are able to discern between fact and fabrication, resistant to manipulation, and have more stable policy attitudes [27,31,84]. The proposed intervention — found promising across numerous field quasi-experiments on one of the most popular social media platforms — can make citizens more resilient to various democratic threats. With 4.95 billion people around the world actively using social media, 239 million in the U.S. alone, adapting such versatile and scalable interventions is of societal interest.

### Significance statement

We introduce a scalable intervention to put factual and verified information on topics of civic importance in users’ social media feeds toward making citizens better informed and more resilient to various democratic threats. We conducted 48 field quasi-experiments using Instagram ads targeting news non-users (reach 2,496,878 accounts, 690,470 watches, 40,584 post-test assessments) to enhance their belief accuracy, democratic attitudes, and behavioral intentions related to climate change, COVID-19 vaccines, digital literacy, and election integrity. We show that 96% of the quasi-experiments were effective across topics and formats, and the effects did not dissipate with time. These findings show the potential of ad-based interventions to disrupt informational silos on social media and provide a robust method to bolster democratic resilience.

## Supporting information

S1 Text Supplementary information. All supporting information, extended analyses and methodological detail.
(DOCX)
