# Supplementary material for: Ad-based social media interventions increase belief accuracy and generate pro-social opinions among non-news readers
Source: PLoS One. 2026 Jun 29;21(6):e0352588. doi: 10.1371/journal.pone.0352588 (PMC13313353; doi:10.1371/journal.pone.0352588)
Supplement: S1 Text — All supporting information, extended analyses and methodological detail. (DOCX) [file pone.0352588.s001.docx]

**Supplementary Information (S1)**

# S1 Section 1. Partnership - Background

Founded in August 2020, Reality Team is a non-profit (501(c)(3)) working to offer credible information in social media feeds in naturalistic settings among users. It is supported by donations and grants from individuals and private foundations. For more information, see <https://realityteam.org/>. Reality Team is primarily concerned with fighting misinformation on social media by correcting it, enhancing media literacy skills, and/or promoting factual, credible and diverse information. Reality Team uses the tools and techniques of digital marketing to make credible information more visible. It runs factual ads with all sources of information clearly and openly noted in the content delivered to the users. The targeted campaigns change the ratio of good to “bad” (e.g., clickbaity, misinformative, radical, etc.) information on people’s social media feeds. The campaigns run by Reality Team have used Instagram ads to combat election, vaccine, and climate mis- and disinformation and promote digital literacy (see details Section 2).

The collaboration between Reality Team and the involved academics started early in 2024. Public research conducted solely by employees of Reality Team could face barriers reaching academic audiences and could possibly encounter skepticism if not accompanied by systematic academic analyses. At the same time, the involved academics would encounter challenges running the quasi-experiments by themselves (without the additional know-how of Reality Team). This collaboration is a way to conduct rigorous scientific analyses necessary to answer questions about the impact of digital interventions on social media platforms in naturalistic settings.

All the costs associated with the research (e.g., ad preparation, targeted campaigns, etc.) were covered by Reality Team before the commencement of this collaboration. None of the academic team members nor their institutions received financial or any other compensation from Reality Team. In addition, all the analyses reported in this manuscript were done by the academic authors. Furthermore, Reality Team agreed that there would be no pre-publication approval on the basis of the findings and Reality Team was solely entitled to contribute to and review the manuscript, but could not request any substantive changes.

**S1 Fig 1**. Design of the Instagram Video Poll method


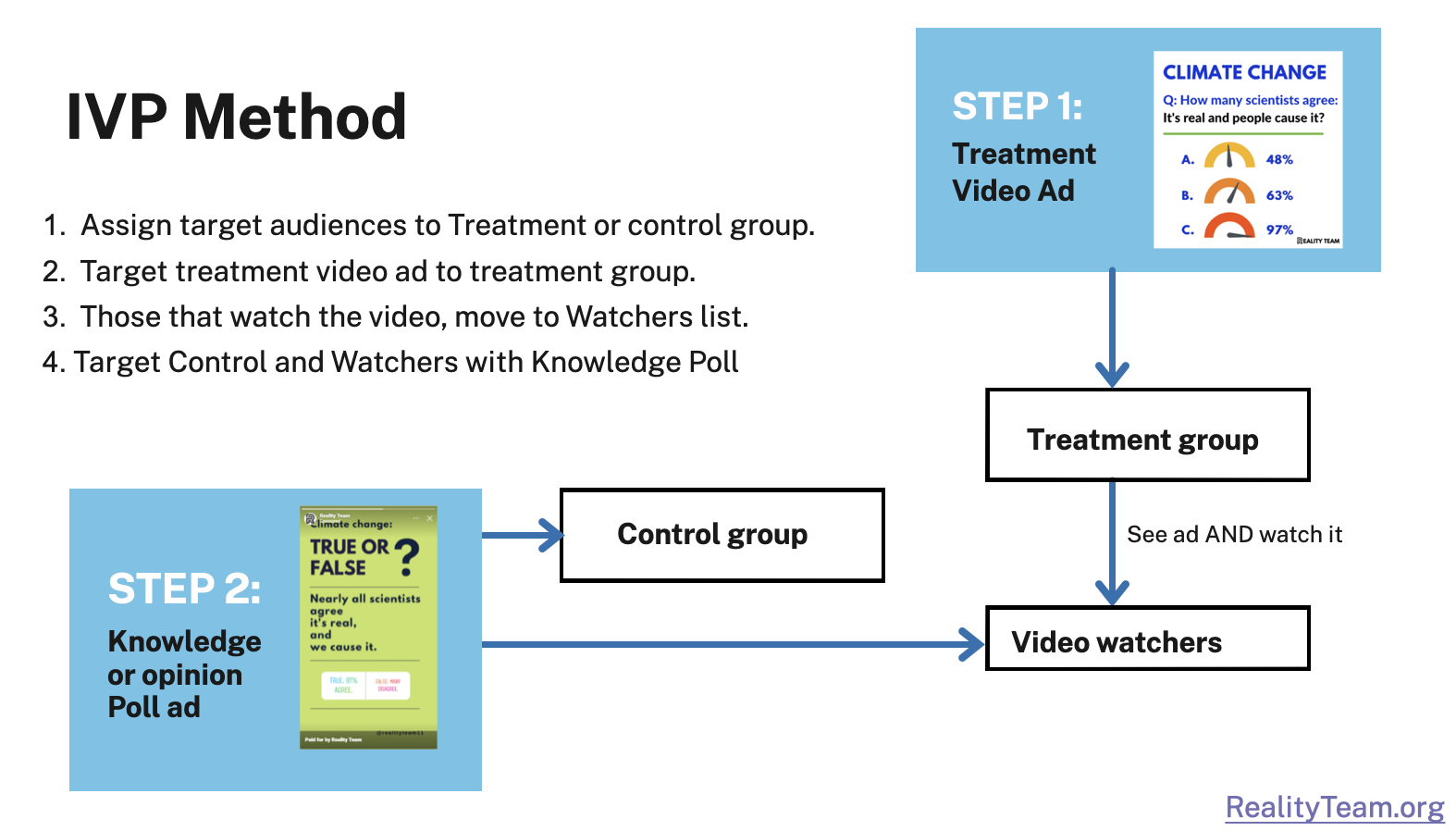


**S1 Table 1. Sample sizes of the post-test assessment polls**

| Label | N Control | N Treatment |
| --- | --- | --- |
| Climate Consensus (A, 2021, MI, BA) | 1040 | 283 |
| Climate Consensus (D, 2022, MI, BA) | 206 | 110 |
| Vaccine 1 (A, 2022, MI, BA) | 204 | 220 |
| Vaccine 2 (A, 2022, MI, BehI) | 288 | 201 |
| Vaccine 3 (2022, MI, BA) | 273 | 201 |
| Vaccine 2 (B, 2022, MI, BehI) | 235 | 161 |
| Vaccine 1 (B, 2022, OH, BA) | 262 | 170 |
| Election Integrity 1 (A, 2022, PA, BA) | 297 | 84 |
| Election Integrity 2 (A, 2022, PA, BA) | 253 | 205 |
| Clean Energy 1 (A, 2022, NC, BA) | 240 | 135 |
| Clean Energy 1 (B, 2022, NC, BA) | 627 | 203 |
| Climate Consensus (B, 2022, NC, BA) | 1004 | 200 |
| Climate Consensus (E, 2022, NC, BA) | 665 | 222 |
| Climate Consensus (F, 2022, NC, BA) | 274 | 468 |
| Clean Energy 2 (A, 2022, TN, BA) | 356 | 157 |
| Clean Energy 2 (B, 2022, TN, BA) | 351 | 135 |
| Clean Energy 2 (C, 2022, TN, BA) | 313 | 216 |
| Clean Energy 2 (D, 2022, TN, BA) | 319 | 151 |
| Clean Energy 3 (C, 2022, TN, BA) | 337 | 98 |
| Clean Energy 3 (D, 2022, TN, BA) | 280 | 106 |
| Clean Energy 3 (A, 2022, TN, BA) | 318 | 117 |
| Clean Energy 3 (B, 2022, TN, BA) | 306 | 77 |
| Clean Energy 4 (A, 2022, TN, BA) | 644 | 201 |
| Clean Energy 4 (B, 2022, TN, BA) | 790 | 195 |
| Election Integrity 1 (2022, PA, BA) | 676 | 247 |
| Election Integrity 2 (2022, PA, BA) | 2073 | 2199 |
| Election Integrity 3 (2022, PA, A) | 2039 | 505 |
| Election Integrity 4 (2022, AZ, BA) | 877 | 224 |
| Election Integrity (A, 2022, NC, BehI) | 217 | 210 |
| Election Integrity 5 (2022, NC, BA) | 221 | 278 |
| Election Integrity (B, 2022, NC, BehI) | 224 | 252 |
| Climate Change Awareness 1 (T1, 2024, NC,BA) | 692 | 221 |
| Climate Change Awareness 1 (T2, 2024, NC,BA) | 703 | 214 |
| Climate Change Awareness 1 (T3, 2024, NC,BA) | 471 | 170 |
| Climate Change Awareness 1 (T4, 2024, NC,BA) | 741 | 116 |
| Climate Change Awareness 2 (T1, 2023, NC, BA) | 772 | 300 |
| Climate Change Awareness 2 (T2, 2023, NC, BA) | 785 | 260 |
| Climate Change Awareness 2 (T3, 2023, NC, BA) | 441 | 225 |
| Climate Change Awareness 2 (T4, 2023, NC, BA) | 962 | 183 |
| Clean Energy (T1, 2023, NC, A) | 644 | 212 |
| Clean Energy (T2, 2023, NC, A) | 867 | 201 |
| Clean Energy (T3, 2023, NC, A) | 666 | 209 |
| Clean Energy (T4, 2023, NC, A) | 538 | 144 |
| Climate Consensus (C, 2023, GA, BA) | 334 | 530 |
| Election Integrity (A, 2023, AZ, BA) | 268 | 254 |
| Election Integrity (B, 2023, AZ, BA) | 329 | 202 |
| Media literacy 1 (2023, WI, BA) | 238 | 144 |
| Media literacy 2 (2023, WI, ML) | 659 | 282 |
| Media literacy 3 (2023, WI, ML) | 376 | 255 |
| Media literacy 4 (2024, MI, ML) | 624 | 364 |
| Clean Energy (B, 2023, NC, A) | 295 | 253 |

*Note.* The labels indicate the topic and version of the post-test assessment. Letters are used when the same assessment was utilized in more than one quasi-experiment (A if same assessment, B if different assessments were used). T1 to T4 indicates quasi-experiments with delayed measurements. The year the quasi-experiment was conducted, the state in which the quasi-experiment took place, and the focus of the assessment (BA for belief accuracy, A for attitude, ML for Media Literacy, and BehI for Behavioral Intention) are also included. M stands for Michigan, OH stands for Ohio, PA stands for Pennsylvania, NC stands for North Carolina, TN stands for Tennessee, GA stands for Georgia, WI stands for Wisconsin.

Example quasi-experiment label: "Clean Energy 4 (B, 2022, TN, BA)"

- **Topic:** Clean Energy
- **Post-test assessment Version:** 4
- **Measurement:** B (same assessment used)
- **Year:** 2022
- **State:** Tennessee (TN)
- **Focus:** Belief accuracy (BA)

# S1 Section 2. Topics and Treatments

This section of the SM describes the topics of the quasi-experiments and presents two examples of treatments per topic. Given that the treatments were distributed using Instagram ads in naturalistic settings, they were all relatively short and visually appealing (e.g., <https://www.facebook.com/ads/library/?id=511406430600768>). We also put considerable effort into ensuring that only true, verified, and constructive statements are included and tested in the treatments to avoid any possibility of spreading mis- or disinformation.

All the quasi-experiments addressed one of four current and societally consequential topic areas: Climate Change, COVID-19 Vaccines, Media Literacy, and Election Integrity. We reviewed extensive research on message strategies that effectively change opinions on accepting the facts of climate change, mitigating vaccine hesitancy, informing citizens about elections and election integrity and correcting election-related misinformation, and enhancing digital literacy skills. In addition, we relied on interviews with community members and other available research, such as focus groups run by credible NGOs, PEW Research data, and other sources to design the treatments, adapt relevant theoretical informational strategies, and use the most effective language and visual presentation in the treatments.

Reality Team deployed interventions in different locations based on varying criteria, including average ad buy cost (larger, more populated states tend to have less expensive ad rates), a desire to avoid recruiting overlapping populations for studies if too many recruited from the same geographic locals, constraints imposed by their own funders (for instance, for some climate change messaging, certain states and rural versus urban areas were funder priorities), and media reports that audiences in ‘swing states’ were disproportionately more likely to be exposed to political mis- and disinformation narratives (e.g., Pratelli et al., 2024).

## Section 2.1 Climate Change

The topics covered in our interventions address three categories. The first topic is related to the scientific consensus that climate change is real and caused by humans. The second topic focused on clean energy solutions, demonstrating that the problem of climate change is solvable. The third topic focused on the economic benefits of climate solutions - especially with regard to job creation. We relied on a previously published compilation of results from several dozen message strategies informing about climate change. The compilation found a high correlation between knowing the scientific consensus around climate change and believing that climate change is real. As such, this strategy became the basis of many of the quasi-experiments.

**S1 Fig 2.** Examples of climate change treatments


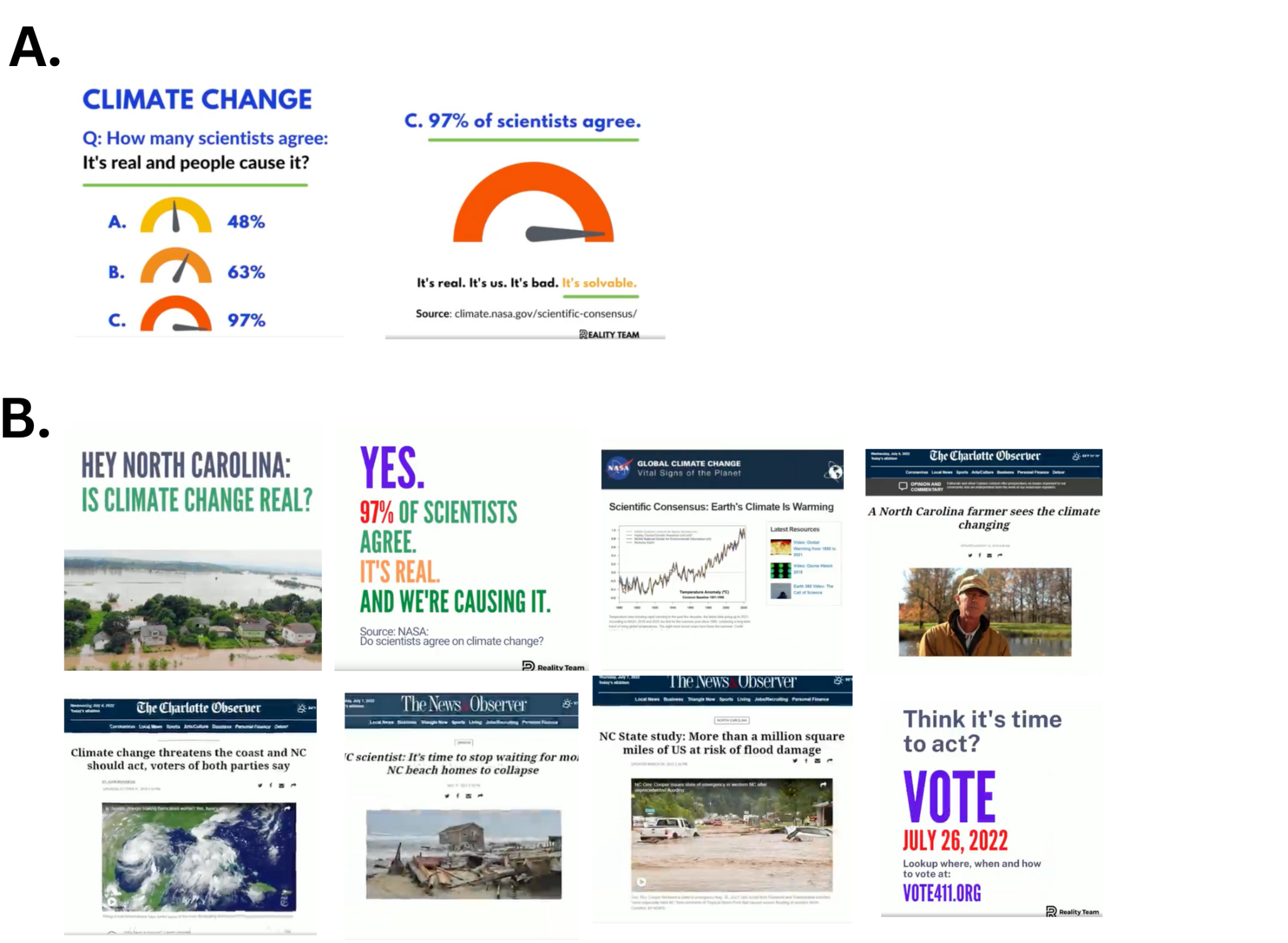


## Section 2.2 COVID-19 Vaccine Safety

In studying this topic, we tested two main messages. The first informed the users about the relative risk of vaccine side effects vs getting struck by lightning, an approach motivated by focus groups and survey findings that people were concerned about vaccine side effects and did not know how to think about the relative risks of these side effects. The second message strategy informed the users about the percentage of medical doctors who were vaccinated at the time of the quasi-experiment. This was designed to incorporate the persuasive effect of ’expert power’ at the time that indicated physicians were considered one of the most trusted messengers for vaccine-hesitant people, and research indicating the effectiveness of consensus messaging for scientific communications (Dixon et al., 2015, Hamel & Brodie, 2021).

**S1 Fig 3.** Examples of vaccine safety treatments


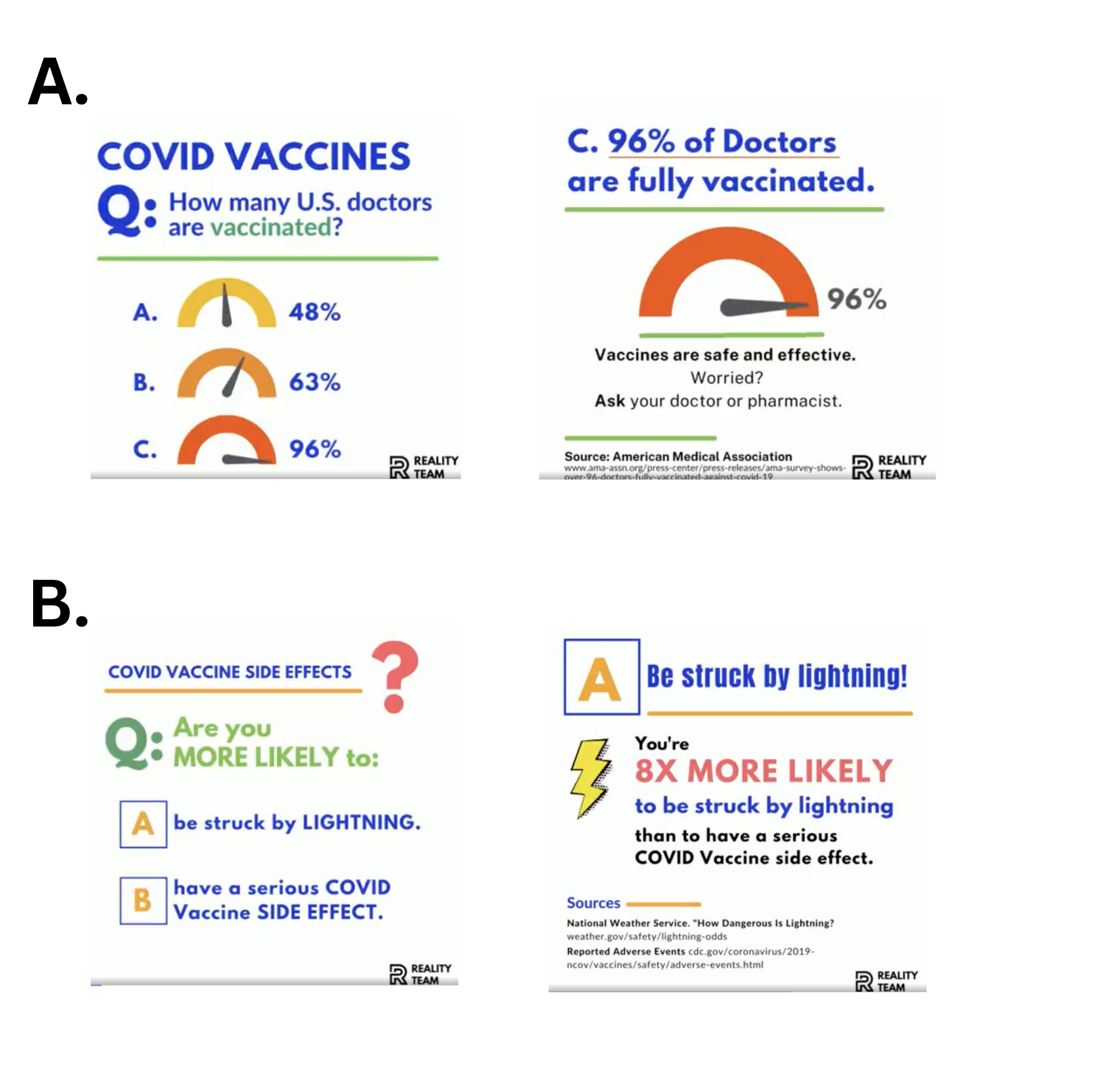


## Section 2.3 Election Integrity

Based on reports that misinformation about the results of the 2020 US presidential election was circulating widely on social media and likely to influence trust in 2022 midterm election results, we designed quasi-experiments to test whether amplifying the viewpoints of several trusted sources could increase acceptance of the 2020 election results. We used a common Reality Team message format of “5 reasons to believe...”. We chose quotes and findings from public figures and agencies that represented a range on the political spectrum, listing the sources at the end of the ad. In addition, to test whether we could increase trust in electoral processes, we designed two quasi-experiments. In Pennsylvania, we highlighted the number of people who work and volunteer on election day in that state. Subsequent research found that public information videos providing facts about the electoral system does increase voter trust (Gaudette et al., April 11, 2023). In Arizona, we highlighted the use of local schools and churches as election polling places in those communities, with the expectation that these are perceived as reliable community-based locations and therefore increase trust in and reduce possible skepticism about election processes. To maximize memory retention, we used various visuals and the multiple choice quiz format (Collier, Pillai, and Fazio, L., 2023).

**S1 Fig 4.** Examples of election integrity treatments


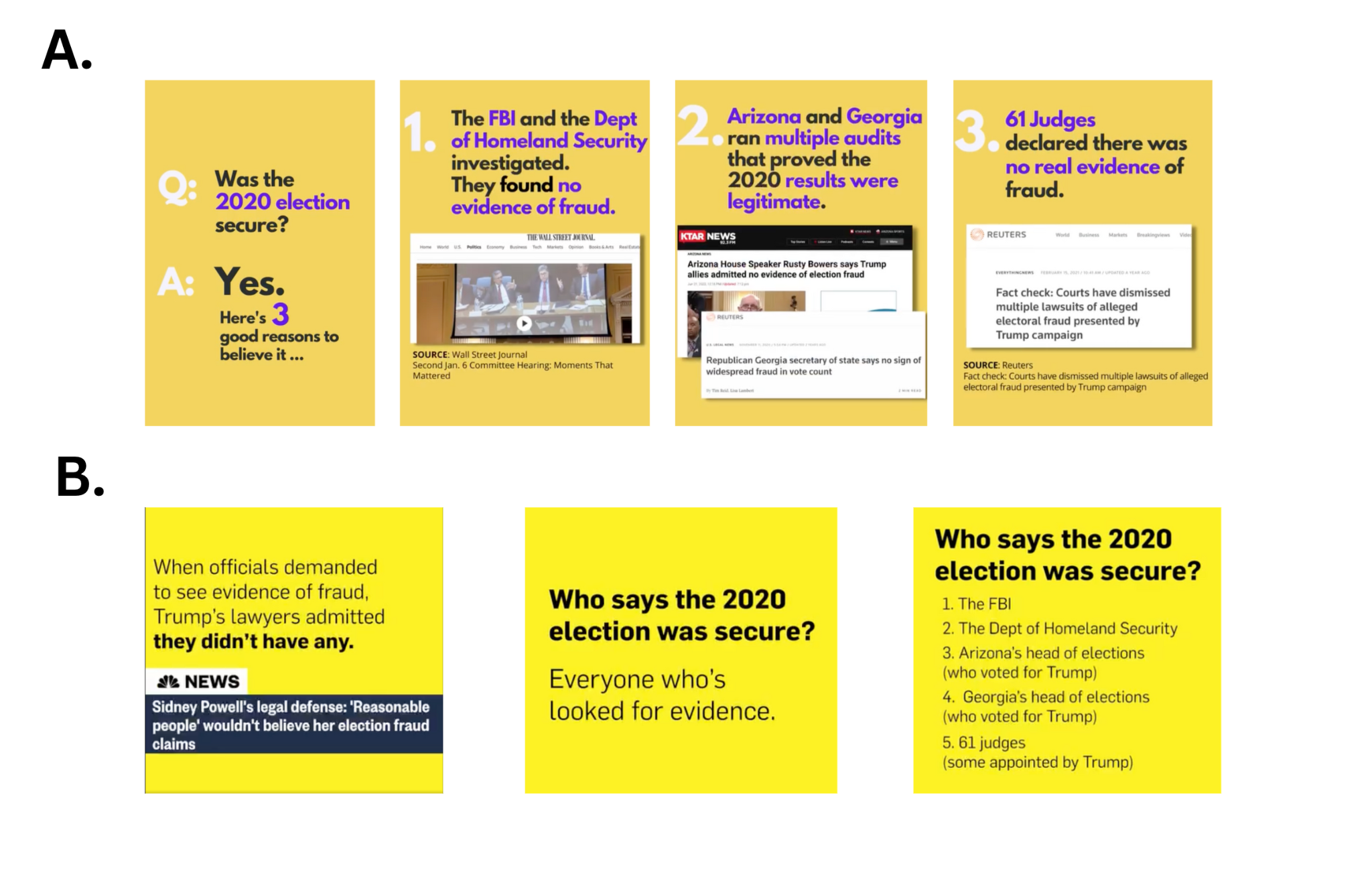


## Section 2.4 Media Literacy

Lastly, we ran quasi-experiments to increase media literacy of social media users as related to well-documented logical fallacies that are commonly used to manipulate individuals. Our primary objective was to further support the potential scalability of protective interventions by reducing the length of treatment to be more aligned with typical ad-based content on Instagram. Drawing on recent research (e.g., Baozyreva, Lewandowsky, & Hertwig, 2020; Roozenbeek et al., 2022), we wanted to translate the academic findings to our very short Instagram ad-based methodology. In preparing the treatments, we studied the 90-second YouTube video lessons developed by Roozenbeek et al. (2022).^^[[1]](#footnote-1)^^ We chose a subset of three manipulative techniques that mapped to the manipulation techniques used in their study (ad hominem, false dichotomy, and loaded language). Using both text and graphics, we incorporated multiple best practices identified by prior research, e.g., to include a forewarning that disinformation is a problem and to be on alert for it, as well as a short accuracy nudge at the end.

**S1 Fig 5.** Examples of election media literacy


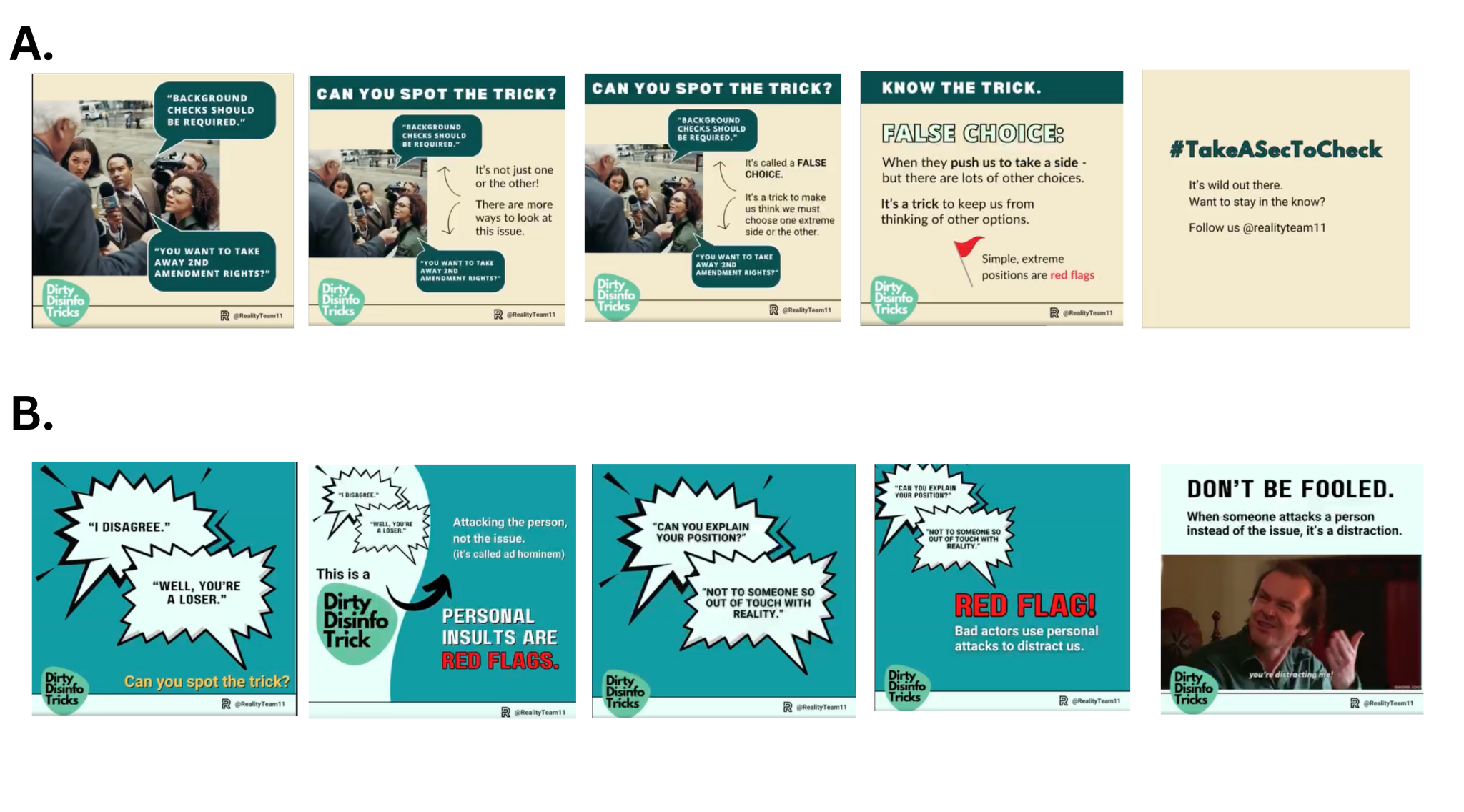


# SM Section 3. Content and Format Features

In contrast to the topical variation, the ads used with the IPV methodology share several structural features in both form and content, while also differing on key aspects that facilitated analyses to assess the relative efficacy of differing intervention types.

## Section 3.1 Persuasive Appeals.

Whereas all information presented in intervention ads was fact-checked, the particular advertisements make use of several distinct persuasive appeals. To identify potential outcome differences, we categorized all ads as belonging to one of four categories based on the most prominently invoked appeal: source credibility, social norms, benefits, and logical inference.

- **Source credibility:** Advertisements emphasizing source credibility included appeals to medical and scientific experts as well as attributed **specific** statements **to** relevant institutions and individual experts. These appeals rely on expertise—as in the case of noting the prevailing opinion of scientists—as well as trustworthiness—for instance using statements from relevant Republican officials in relation to election misinformation**,** which spread primarily among conservatives (Sussman, 2003).
- **Social norms:** Social norms can be understood in terms of injunctive norms, which refer to the actions others approve or disapprove of, and descriptive norms, which refer to the actual behavior of others (Cialdini et al., 1990). Most advertisements relied on descriptive norms, emphasizing that people in a target state were often employed in green energy jobs or that much of election security is managed by local community members.
- **Benefits:** Benefits can be understood in terms of self-benefits (i.e., personal gain) and other-benefits (i.e., gains for the broader community or other people; White & Peloza, 2009). While a few advertisements made indirect appeals to self-benefits (e.g., noting the cheap cost of electric cars), many focused on social goods and other-benefits (e.g., ads noting that green energy is a growing industry which can help create a strong economy for a target state).
- **Logical inference:** These ads invoked “math” or other declarations of sound and logical reasoning and provided rational arguments regarding the basic facts of issues, which participants could understand and endorse by virtue of argument quality.

## Section 3.2 Format Features.

All interventions consisted of short video advertisements inserted into participants’ Instagram feeds. No advertisements featured sound, and all advertisements relied primarily on text to communicate information. However, the ads differed in a few relevant format features related to content richness. For analytic purposes, we categorized the ads based on the inclusion of differing features. When multiple features were included, ads were categorized into the “richest” of the four categories below.

1. Text only
2. Text + Informational graphics
3. Text + Drawings
4. Text + Realistic images

On the one hand, the ads presented information simply and directly, and so richer or more intense media might distract from the information presented and increase attentional demands on participants (Robert & Dennis, 2005). On the other hand, the presence of images might improve any effects of video advertisements (Li, 2024).

# S1 Section 4. Randomization and Self-Selection Bias

As mentioned in the main text, given the nature of the treatment implementation, we had no control over who saw the treatment ads and watched the treatment videos. For instance, not everyone who saw the treatment ads responded to the short in-feed post-test assessments, which means that we may have different opt-in biases between control, treatment, and those who respond to the post-test. Because it is difficult to ascertain perfect random assignment and self-selection biases in field quasi-experiments, we attempted to test whether randomization to treatments and control groups was successful and whether there were opt-in biases in exposure to the treatments and completing the post-test assessment by comparing the distribution of age and gender of individuals who saw and engaged with the ads in the control and treatment groups. These two characteristics were retrieved from Meta’s Ads Manager, which provides statistics on reach, number of impressions, clicks, and ad post engagement by age group and gender. Meta does not offer any additional user information, which could facilitate the comparisons on other variables. We used Chi-squared tests to evaluate whether there were statistically significant differences in the demographic characteristics between the groups. Also, given the numerous significance tests performed, we corrected for multiple comparisons using Bonferroni correction separately for each group of tests.

These findings indicate that despite ineffective randomization and the expected self-selection to watching the treatment video, those who completed the post-assessment ads in the treatment and control were similar on gender, but less so on age. Although limited, these analyses offer some assurance that the detected results are not solely attributable to self-selection on these two factors. We acknowledge that Meta does not offer any additional data on users, which could facilitate comparisons on potentially more consequential variables, such as partisanship. In general, it is difficult to ascertain randomization and minimize opt-in biases in field experiments, and our project is limited in this regard. Nevertheless, the at-scale evidence offered below may be valuable to scholars and practitioners.

The adjusted p-values for gender distribution are presented in Table 2 and for age distribution are shown in Table 3. Additionally, for cases where the chi-squared test indicated statistical significance, Tables 4 and 5 show Cramér’s V values for all pairwise comparisons.

**S1 Fig 6.** Quasi**-**experimental and randomization design schema, including metrics used to verify randomization and self-selection bias.


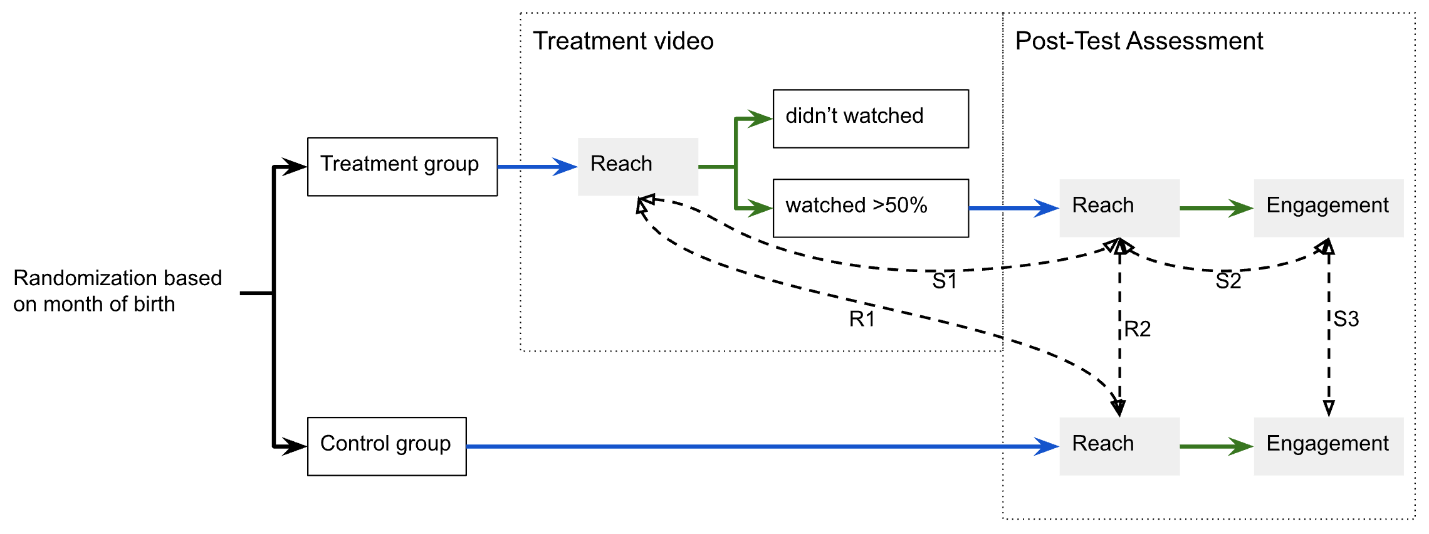


Note: Shaded gray areas represent the metrics measured within gender and age groups, which are used for statistical tests. Blue arrows represent the functioning of ad delivery algorithms; green arrows indicate user engagement, illustrating interactions with ads (e.g., viewing or completing post-test assessments). Dashed lines depict the comparisons made in each test, focusing on randomization (e.g., between treatment and control groups) and self-selection bias (e.g., between users reached by ads and those who engaged).

For randomization, we compared the gender and age distributions in two ways:

1. First, we compare users reached by treatment video and the post-test assessment ad in the control [R1]. As shown in SM Section 5 Tables 2 and 3 (first column), we find significant differences between the groups (p < 0.05). All the quasi-experiments have significant differences in gender and age distribution. The analysis revealed that gender-related differences, as measured by Cramér’s V, generally fall within the range of 0.1–0.2 or lower, indicating a weak to moderate association. In contrast, age-related differences exhibit substantially higher values of Cramér’s V, suggesting stronger associations and greater disparities between groups.
2. Second, we compare gender and age of those reached by the post-test assessment ad in the treatment and control groups [R2] (second column in Tables 2 and 3). We find that only five quasi-experiments have similar gender distribution and none for age. Cramér’s V values for gender are below 0.1 in most cases, while the remaining values fall between 0.1 and 0.23, indicating weak to moderate associations. In contrast, the differences in age are more pronounced, with Cramér’s V values exceeding 0.3 in 17 quasi-experiments, while the rest range between 0.1 and 0.3. These findings suggest substantial differences in the age structure between the compared groups.

These results indicate that the randomization was not effective and that these differences are attributable to Meta’s ads delivery algorithm. If Meta's advertising system had displayed ads entirely at random to individuals in the target group, as was intended by the design, we would expect no significant differences by gender and age between the treatment and control groups. However, the detected differences show that the Meta advertising system does not assign users randomly but instead delivers ads to different users with some systematic differences. In short, the *reach* of the videos was determined by the Meta algorithm.

For self-selection, we compared age and gender in a few ways:

1. We compared individuals who were shown the treatment ads with those who watched at least 50% of the treatment video and later were reached by post-test assessment [S1]. Column 3 Tables 2 and 3 in SM Section 5 show significant differences in terms of age in the majority of quasi-experiments and in terms of gender in about half of them, suggesting *self-selection in terms of watching* (as can be expected; one of the reasons why controlled experiments lack ecological validity is that they do not allow people to self-select into treatment but ‘force’ all participants to see it, which does not reflect information exposure in naturalistic settings). Cramér’s V values for gender differences are very small (below 0.1), suggesting that any observed variation in gender distribution is minor and may not substantially influence the outcomes of the study. For age differences, the values range between 0.1 and 0.3 in 18 quasi-experiments and exceed 0.3 in only 3 cases, suggesting that individuals of certain age groups may have been more or less likely to watch the treatment video.
2. Importantly, we also compare those who received the ad with the post-test assessment and those who completed it [S2], a more direct test of self-selection. Here, there were no significant differences for gender in 92% of the quasi-experiments, and the differences for age were absent in two-thirds of the quasi-experiments. In almost all cases, Cramér’s V values were below 0.1, with only 6 quasi-experiments showing values between 0.1 and 0.2 for comparisons of age structure. *This suggests that despite the initial self-selection to watching, self-selection in terms of seeing the post-assessment ad and completing it is much smaller*.
3. The most important comparison is between the users in control and treatment who responded to the post-test assessment ads [S3]. Here, we find no statistically significant differences in gender between the treatment and control groups in 81% of the quasi-experiments. However, for age, the differences are significant in 91% of quasi-experiments. In most of them, Cramér’s V values are over 0.3.

In sum, these findings indicate that randomization was not fully effective on these two characteristics due to Meta’s ad delivery systems, which determines who sees ads. We also find (an expected) self-selection to watching the treatment video. However, self-selection bias was substantially lower for the key engagement phase. That is, those in the treatment group who received the post-assessment ad do not substantially differ from those who clicked on it, and we achieve a more balanced representation in terms of the treatment and control users who engaged with the post-treatment quizzes. These additional analyses offer some assurance that the results are not likely due to self-selection alone even though the detected differences may suggest that certain age groups are more likely to watch Instagram video ads and partake in in-line quizzes than others. Given the lack of data from Meta, we cannot ascertain if there are self-selection biases across more directly relevant variables, e.g. individual partisanship, ideology, or prior issue attitudes.

**S1 Table 2. Results of Chi-square tests (p-values) comparing the Gender structure between treatment and control groups across different quasi-experiments.**

|  | Test of Randomization | | Test of Self-Selection | | |
| --- | --- | --- | --- | --- | --- |
|  | [R1] Reach of Treatment Video Ad (TG) vs Reach of Post-test (CG) | [R2] Reach of Post-test in TG vs in CG | [S1] Reach of Treatment Video Ad (TG) vs Reach of Post-test (TG) | [S2] Reach of Post-test (TG) vs Engagement of Post-test (TG) | [S3] Post-test Engagement in TG vs CG |
| Clean Energy (A, 2023, NC) | NA | 0 | NA | 1 | 1 |
| Clean Energy (B, 2023, NC) | NA | 0 | NA | 1 | 0.024 |
| Clean Energy (T1A, 2023, NC) | NA | 0 | NA | 1 | 0.006 |
| Clean Energy (T1B, 2023, NC) | NA | 0 | NA | 1 | 1 |
| Clean Energy (T1C, 2023, NC) | NA | 0 | NA | 1 | 0.344 |
| Clean Energy (T2A, 2023, NC) | NA | 0 | NA | 1 | 0.428 |
| Clean Energy (T2B, 2023, NC) | NA | 0 | NA | 1 | 1 |
| Clean Energy (T2C, 2023, NC) | NA | 0 | NA | 1 | 1 |
| Clean Energy (T3A, 2023, NC) | NA | 0 | NA | 1 | 1 |
| Clean Energy (T3B, 2023, NC) | NA | 0 | NA | 1 | 1 |
| Clean Energy (T3C, 2023, NC) | NA | 0 | NA | 1 | 1 |
| Clean Energy (T4A, 2023, NC) | NA | 0 | NA | 1 | 0.449 |
| Clean Energy (T4B, 2023, NC) | NA | 0 | NA | 1 | 1 |
| Clean Energy (T4C, 2023, NC) | NA | 0 | NA | 1 | 1 |
| Clean Energy 1 (A, 2022, NC) | 0 | 0 | 0.001 | 1 | 1 |
| Clean Energy 1 (B, 2022, NC) | 0 | 0 | 0 | 1 | 1 |
| Clean Energy 2 (A, 2022, TN) | 0 | 0 | 0.003 | 1 | 1 |
| Clean Energy 2 (B, 2022, TN) | 0 | 0 | 0.681 | 1 | 1 |
| Clean Energy 2 (C, 2022, TN) | 0 | 0 | 0.08 | 1 | 0.794 |
| Clean Energy 2 (D, 2022, TN) | 0 | 0 | 0 | 1 | 1 |
| Clean Energy 3 (A, 2022, TN) | 0 | 0 | 0 | 1 | 0.016 |
| Clean Energy 3 (B, 2022, TN) | 0 | 0 | 0.016 | 1 | 1 |
| Clean Energy 3 (C, 2022, TN) | 0 | 0 | 0 | 1 | 0.252 |
| Clean Energy 3 (D, 2022, TN) | 0 | 0 | 1 | 1 | 0 |
| Clean Energy 4 (A, 2022, TN) | 0 | 0 | 0.013 | 0.916 | 0.001 |
| Clean Energy 4 (B, 2022, TN) | 0 | 0 | 0 | 1 | 0 |
| Climate Change Awareness 1 (T1A, 2023, NC) | 0 | 0 | 1 | 1 | 1 |
| Climate Change Awareness 1 (T1B, 2023, NC) | 0 | 0 | 1 | 1 | 0.215 |
| Climate Change Awareness 1 (T1C, 2023, NC) | 0 | 0 | 1 | 1 | 1 |
| Climate Change Awareness 1 (T2A, 2023, NC) | 0 | 0 | 1 | 1 | 1 |
| Climate Change Awareness 1 (T2B, 2023, NC) | 0 | 0 | 1 | 1 | 0.59 |
| Climate Change Awareness 1 (T2C, 2023, NC) | 0 | 0 | 1 | 1 | 1 |
| Climate Change Awareness 1 (T3A, 2023, NC) | 0 | 0 | 1 | 1 | 1 |
| Climate Change Awareness 1 (T3B, 2023, NC) | 0 | 0 | 1 | 1 | 0.123 |
| Climate Change Awareness 1 (T3C, 2023, NC) | 0 | 0 | 1 | 1 | 0.047 |
| Climate Change Awareness 1 (T4A, 2023, NC) | 0 | 0 | 1 | 1 | 1 |
| Climate Change Awareness 1 (T4B, 2023, NC) | 0 | 0 | 1 | 1 | 1 |
| Climate Change Awareness 1 (T4C, 2023, NC) | 0 | 0 | 1 | 1 | 1 |
| Climate Change Awareness 2 (T1A, 2023, NC) | 0 | 0 | 1 | 1 | 1 |
| Climate Change Awareness 2 (T1B, 2023, NC) | 0 | 0 | 0 | 1 | 1 |
| Climate Change Awareness 2 (T1C, 2023, NC) | 0 | 0 | 1 | 1 | 0.273 |
| Climate Change Awareness 2 (T2A, 2023, NC) | 0 | 0 | 1 | 1 | 1 |
| Climate Change Awareness 2 (T2B, 2023, NC) | 0 | 0 | 0 | 1 | 1 |
| Climate Change Awareness 2 (T2C, 2023, NC) | 0 | 0 | 1 | 1 | 1 |
| Climate Change Awareness 2 (T3A, 2023, NC) | 0 | 0 | 1 | 1 | 0.004 |
| Climate Change Awareness 2 (T3B, 2023, NC) | 0 | 0 | 0 | 1 | 1 |
| Climate Change Awareness 2 (T3C, 2023, NC) | 0 | 0 | 1 | 1 | 1 |
| Climate Change Awareness 2 (T4A, 2023, NC) | 0 | 0 | 1 | 1 | 0.167 |
| Climate Change Awareness 2 (T4B, 2023, NC) | 0 | 0 | 0 | 1 | 1 |
| Climate Change Awareness 2 (T4C, 2023, NC) | 0 | 0 | 1 | 1 | 1 |
| Climate Consensus (A, 2021, MI) | 0 | 0 | 0 | 0.009 | 1 |
| Climate Consensus (B, 2022, NC) | 0 | 0 | 0 | 1 | 0.11 |
| Climate Consensus (C, 2023, GA) | 0 | 0.208 | 0.654 | 1 | 1 |
| Climate Consensus (D, 2022, MI) | 0 | 0.566 | 1 | 1 | 1 |
| Climate Consensus (E, 2022, NC) | 0 | 0 | 0 | 1 | 0.061 |
| Climate Consensus (F, 2022, NC) | 0 | 0 | 0 | 1 | 0 |
| Election Integrity (A, 2022, NC.) | NA | 0.005 | NA | 1 | 0.771 |
| Election Integrity (A, 2023, AZ) | 0 | 0 | 1 | 1 | 0.553 |
| Election Integrity (B, 2022, NC) | 0 | 0.142 | 1 | 1 | 1 |
| Election Integrity (B, 2023, AZ) | 0 | 0 | 0 | 0.296 | 1 |
| Election Integrity 1 (2022, PA) | 0 | 0 | 0 | 1 | 0.865 |
| Election Integrity 2 (2022, PA) | 0 | 0 | 0 | 0 | 0 |
| Election Integrity 3 (2022, PA) | 0 | 0 | 0.09 | 1 | 0.007 |
| Election Integrity 4 (2022, AZ) | 0 | 0 | 0.023 | 0.196 | 1 |
| Election Integrity 5 (2022, NC) | 0.027 | 1 | 1 | 1 | 1 |
| Media literacy 1 (A, 2023, WI) | 0 | 1 | 0 | 0 | 0 |
| Media literacy 1 (B, 2023, WI) | 0 | 0 | 0.302 | 0 | 0 |
| Media literacy 2 (A, 2023, WI) | 0 | 0 | 0.009 | 0 | 0 |
| Media literacy 3 (2024, MI) | 0 | 0 | 0 | 0 | 0 |
| Vaccine 1 (A, 2022, MI) | 0 | 0 | 0 | 1 | 1 |
| Vaccine 1 (B, 2022, OH) | 0 | 0 | 0 | 1 | 0.064 |
| Vaccine 2 (A, 2022, MI.) | NA | 0 | NA | 0.386 | 1 |
| Vaccine 2 (B, 2022, MI.) | NA | 0 | NA | 1 | 1 |
| Vaccine 3 (2022, MI) | 0 | 0 | 0 | 1 | 1 |

Note: This table presents the p-values from Chi-square tests comparing the gender structure between treatment and control groups across various quasi-experiments. In parentheses, letters are used when the same assessment was utilized in more than one quasi-experiment: A if same assessment, B if different assessments were used; the year, and the state: M Michigan, OH Ohio, PA Pennsylvania, NC North Carolina, TN Tennessee, GA Georgia, WI Wisconsin. The lack of significant differences is highlighted with a grayed-out background. The first two columns assess the effectiveness of randomization by comparing the gender distributions of users reached by the quasi-experimental intervention and those reached by the control group assessment (1st column) and users reached by the post-test assessment in both groups (2nd). The third and fourth columns test for self-selection bias by comparing the gender distributions in the treatment group: users reached by the intervention and those reached by post-test assessment (3rd), as well as those who were reached by and those who engaged with the treatment post-test (4th). The fifth column evaluates the gender distribution differences between users who engaged with the treatment assessment and those in the control group. NA in the table indicates that the demographic data necessary to perform the test were not available.

**S1 Table 3. Results of Chi-square tests (p-values) comparing the Age structure between treatment and control groups across different quasi-experiments.**

|  | Test of Randomization | | Test of Self-Selection | | |
| --- | --- | --- | --- | --- | --- |
|  | [R1] Reach of Treatment Video Ad (TG) vs Reach of Post-test (CG) | [R2] Reach of Post-test in TG vs in CG | [S1] Reach of Treatment Video Ad (TG) vs Reach of Post-test (TG) | [S2] Reach of Post-test (TG) vs Engagement of Post-test (TG) | [S3] Post-test Engagement in TG vs CG |
| Clean Energy (A, 2023) | NA | 0 | NA | 0.583 | 0 |
| Clean Energy (B, 2023, NC) | NA | 0 | NA | 0 | 0 |
| Clean Energy (T1A, 2023) | NA | 0 | NA | 1 | 0.094 |
| Clean Energy (T1B, 2023) | NA | 0 | NA | 1 | 0.002 |
| Clean Energy (T1C, 2023) | NA | 0 | NA | 1 | 0 |
| Clean Energy (T2A, 2023) | NA | 0 | NA | 1 | 0.004 |
| Clean Energy (T2B, 2023) | NA | 0 | NA | 1 | 0 |
| Clean Energy (T2C, 2023) | NA | 0 | NA | 0.01 | 0 |
| Clean Energy (T3A, 2023) | NA | 0 | NA | 1 | 0 |
| Clean Energy (T3B, 2023) | NA | 0 | NA | 0.101 | 0 |
| Clean Energy (T3C, 2023) | NA | 0 | NA | 1 | 0 |
| Clean Energy (T4A, 2023) | NA | 0 | NA | 1 | 0 |
| Clean Energy (T4B, 2023) | NA | 0 | NA | 1 | 0 |
| Clean Energy (T4C, 2023) | NA | 0 | NA | 1 | 0 |
| Clean Energy 1 (A, 2022) | 0 | 0 | 0 | 1 | 0 |
| Clean Energy 1 (B, 2022) | 0 | 0 | 0 | 1 | 0 |
| Clean Energy 2 (A, 2022) | 0 | 0 | 0 | 1 | 0 |
| Clean Energy 2 (B, 2022) | 0 | 0 | 0 | 1 | 0 |
| Clean Energy 2 (C, 2022) | 0 | 0 | 0 | 0.026 | 0 |
| Clean Energy 2 (D, 2022) | 0 | 0 | 0 | 1 | 0 |
| Clean Energy 3 (A, 2022) | 0 | 0 | 0.001 | 1 | 0 |
| Clean Energy 3 (B, 2022) | 0 | 0 | 1 | 1 | 0 |
| Clean Energy 3 (C, 2022) | 0 | 0 | 0 | 1 | 0 |
| Clean Energy 3 (D, 2022) | 0 | 0 | 0 | 1 | 0 |
| Clean Energy 4 (A, 2022) | 0 | 0 | 0 | 0.081 | 0 |
| Clean Energy 4 (B, 2022) | 0 | 0 | 0 | 0.003 | 0 |
| Climate Change Awareness 1 (T1A, 2023) | 0 | 0 | 0 | 0.39 | 0.001 |
| Climate Change Awareness 1 (T1B, 2023) | 0 | 0 | 0 | 1 | 0 |
| Climate Change Awareness 1 (T1C, 2023) | 0 | 0 | 0 | 1 | 0 |
| Climate Change Awareness 1 (T2A, 2023) | 0 | 0 | 0 | 1 | 0 |
| Climate Change Awareness 1 (T2B, 2023) | 0 | 0 | 0 | 1 | 0 |
| Climate Change Awareness 1 (T2C, 2023) | 0 | 0 | 0 | 1 | 0 |
| Climate Change Awareness 1 (T3A, 2023) | 0 | 0 | 0 | 1 | 0 |
| Climate Change Awareness 1 (T3B, 2023) | 0 | 0 | 0 | 1 | 0 |
| Climate Change Awareness 1 (T3C, 2023) | 0 | 0 | 0 | 0.139 | 0 |
| Climate Change Awareness 1 (T4A, 2023) | 0 | 0 | 0 | 1 | 1 |
| Climate Change Awareness 1 (T4B, 2023) | 0 | 0 | 0 | 1 | 0.005 |
| Climate Change Awareness 1 (T4C, 2023) | 0 | 0 | 0 | 1 | 0 |
| Climate Change Awareness 2 (T1A, 2023) | 0 | 0 | 0 | 0.008 | 0 |
| Climate Change Awareness 2 (T1B, 2023) | 0 | 0 | 0 | 1 | 0 |
| Climate Change Awareness 2 (T1C, 2023) | 0 | 0 | 0 | 0.004 | 0 |
| Climate Change Awareness 2 (T2A, 2023) | 0 | 0 | 0 | 0 | 0 |
| Climate Change Awareness 2 (T2B, 2023) | 0 | 0 | 0 | 1 | 0 |
| Climate Change Awareness 2 (T2C, 2023) | 0 | 0 | 0 | 0.103 | 0 |
| Climate Change Awareness 2 (T3A, 2023) | 0 | 0 | 0 | 0.013 | 0 |
| Climate Change Awareness 2 (T3B, 2023) | 0 | 0 | 0 | 1 | 0 |
| Climate Change Awareness 2 (T3C, 2023) | 0 | 0 | 0 | 0.008 | 0 |
| Climate Change Awareness 2 (T4A, 2023) | 0 | 0 | 0 | 1 | 0 |
| Climate Change Awareness 2 (T4B, 2023) | 0 | 0 | 0 | 1 | 0 |
| Climate Change Awareness 2 (T4C, 2023) | 0 | 0 | 0 | 0.015 | 0 |
| Climate Consensus (A, 2021, MI) | 0 | 0 | 0 | 0 | 0.204 |
| Climate Consensus (B, 2022, NC) | 0 | 0 | 0 | 0.021 | 0 |
| Climate Consensus (C, 2023, GA) | 0 | 0 | 0 | 0 | 0 |
| Climate Consensus (D, 2022, MI) | 0 | 0 | 0 | 0.01 | 1 |
| Climate Consensus (E, 2022, NC) | 0 | 0 | 0 | 1 | 0 |
| Climate Consensus (F, 2022, NC) | 0 | 0 | 0 | 1 | 0 |
| Election Integrity (A, 2022, NC) | NA | 0 | NA | 0.016 | 0 |
| Election Integrity (A, 2023, AZ) | 0 | 0 | 0 | 0.057 | 0.066 |
| Election Integrity (B, 2022, NC.) | 0 | 0 | 0 | 0.919 | 0 |
| Election Integrity (B, 2023, AZ) | 0 | 0 | 0 | 0 | 0 |
| Election Integrity 1 (2022, PA) | 0 | 0 | 0 | 0 | 0 |
| Election Integrity 2 (2022, PA) | 0 | 0 | 0 | 0 | 0 |
| Election Integrity 3 (2022, PA) | 0 | 0 | 0 | 0 | 0 |
| Election Integrity 4 (2022, AZ) | 0 | 0 | 0 | 0 | 0 |
| Election Integrity 5 (2022, NC) | 0 | 0 | 0.011 | 0.093 | 0 |
| Media literacy 1 (A, 2023, WI) | 0 | 0 | 0 | 0 | 0 |
| Media literacy 1 (B, 2023, WI) | 0 | 0 | 0 | 0 | 0 |
| Media literacy 2 (A, 2023, WI) | 0 | 0 | 0 | 0 | 0 |
| Media literacy 3 (2024, MI) | 0 | 0 | 0 | 0 | 0 |
| Vaccine 1 (A, 2022, MI) | 0 | 0 | 0 | 1 | 0.014 |
| Vaccine 1 (B, 2022, OH) | 0 | 0 | 0 | 0.196 | 1 |
| Vaccine 2 (A, 2022, MI) | NA | 0 | NA | 0.73 | 0.002 |
| Vaccine 2 (B, 2022, MI) | NA | 0 | NA | 0.252 | 1 |
| Vaccine 3 (2022, MI) | 0 | 0 | 1 | 0.016 | 0.043 |

Note: This table presents the p-values from Chi-square tests comparing the age structure between treatment and control groups across various quasi-experiments. In parentheses, letters are used when the same assessment was utilized in more than one quasi-experiment: A if same assessment, B if different assessments were used; the year, and the state: M Michigan, OH Ohio, PA Pennsylvania, NC North Carolina, TN Tennessee, GA Georgia, WI Wisconsin. The lack of significant differences is highlighted with a grayed-out background. The first two columns assess the effectiveness of randomization by comparing the age distributions of users reached by the intervention and those reached by the control group assessment (1st column) and users reached by the post-test assessment in both groups (2nd). The third and fourth columns test for self-selection bias by comparing the age distributions in the treatment group: users reached by the intervention and those reached by post-test assessment (3rd), as well as those who were reached by and those who engaged with the treatment post-test (4th). The fifth column evaluates the age distribution differences between users who engaged with the treatment assessment and those in the control group. NA in the table indicates that the demographic data necessary to perform the test were not available.

**S1 Table 4. Cramér’s V values for all pairwise comparisons by gender, presented only for cases where the chi-squared test indicated statistical significance (**adjusted p < 0.05**).**

|  | [R1] Reach of Treatment Video Ad (TG) vs Reach of Post-test (CG) | [R2] Reach of Post-test in TG vs in CG | [S1] Reach of Treatment Video Ad (TG) vs Reach of Post-test (TG) | [S2] Reach of Post-test (TG) vs Engagement of Post-test (TG) | [S3] Post-test Engagement in TG vs CG |
| --- | --- | --- | --- | --- | --- |
| Clean Energy (A, 2023, NC) | NA | 0.12 | NA |  |  |
| Clean Energy (B, 2023, NC) | NA | 0.09 | NA |  | 0.06 |
| Clean Energy (T1A, 2023, NC) | NA | 0.18 | NA |  | 0.25 |
| Clean Energy (T1B, 2023, NC) | NA | 0.08 | NA |  |  |
| Clean Energy (T1C, 2023, NC) | NA | 0.12 | NA |  |  |
| Clean Energy (T2A, 2023, NC) | NA | 0.13 | NA |  |  |
| Clean Energy (T2B, 2023, NC) | NA | 0.06 | NA |  |  |
| Clean Energy (T2C, 2023, NC) | NA | 0.09 | NA |  |  |
| Clean Energy (T3A, 2023, NC) | NA | 0.09 | NA |  |  |
| Clean Energy (T3B, 2023, NC) | NA | 0.04 | NA |  |  |
| Clean Energy (T3C, 2023, NC) | NA | 0.06 | NA |  |  |
| Clean Energy (T4A, 2023, NC) | NA | 0.11 | NA |  |  |
| Clean Energy (T4B, 2023, NC) | NA | 0.06 | NA |  |  |
| Clean Energy (T4C, 2023, NC) | NA | 0.09 | NA |  |  |
| Clean Energy 1 (A, 2022, NC) | 0.14 | 0.10 | 0.02 |  |  |
| Clean Energy 1 (B, 2022, NC) | 0.11 | 0.05 | 0.03 |  |  |
| Clean Energy 2 (A, 2022, TN) | 0.14 | 0.09 | 0.02 |  |  |
| Clean Energy 2 (B, 2022, TN) | 0.10 | 0.06 |  |  |  |
| Clean Energy 2 (C, 2022, TN) | 0.06 | 0.04 |  |  |  |
| Clean Energy 2 (D, 2022, TN) | 0.07 | 0.04 | 0.02 |  |  |
| Clean Energy 3 (A, 2022, TN) | 0.18 | 0.10 | 0.03 |  | 0.18 |
| Clean Energy 3 (B, 2022, TN) | 0.12 | 0.06 | 0.02 |  |  |
| Clean Energy 3 (C, 2022, TN) | 0.18 | 0.08 | 0.04 |  |  |
| Clean Energy 3 (D, 2022, TN) | 0.18 | 0.13 |  |  | 0.28 |
| Clean Energy 4 (A, 2022, TN) | 0.16 | 0.11 | 0.02 |  | 0.15 |
| Clean Energy 4 (B, 2022, TN) | 0.17 | 0.12 | 0.02 |  | 0.21 |
| Climate Change Awareness 1 (T1A, 2023, NC) | 0.18 | 0.14 |  |  |  |
| Climate Change Awareness 1 (T1B, 2023, NC) | 0.14 | 0.10 |  |  |  |
| Climate Change Awareness 1 (T1C, 2023, NC) | 0.19 | 0.15 |  |  |  |
| Climate Change Awareness 1 (T2A, 2023, NC) | 0.19 | 0.14 |  |  |  |
| Climate Change Awareness 1 (T2B, 2023, NC) | 0.13 | 0.07 |  |  |  |
| Climate Change Awareness 1 (T2C, 2023, NC) | 0.17 | 0.11 |  |  |  |
| Climate Change Awareness 1 (T3A, 2023, NC) | 0.19 | 0.15 |  |  |  |
| Climate Change Awareness 1 (T3B, 2023, NC) | 0.17 | 0.12 |  |  |  |
| Climate Change Awareness 1 (T3C, 2023, NC) | 0.18 | 0.12 |  |  | 0.24 |
| Climate Change Awareness 1 (T4A, 2023, NC) | 0.17 | 0.12 |  |  |  |
| Climate Change Awareness 1 (T4B, 2023, NC) | 0.07 | 0.04 |  |  |  |
| Climate Change Awareness 1 (T4C, 2023, NC) | 0.13 | 0.08 |  |  |  |
| Climate Change Awareness 2 (T1A, 2023, NC) | 0.18 | 0.12 |  |  |  |
| Climate Change Awareness 2 (T1B, 2023, NC) | 0.15 | 0.06 | 0.06 |  |  |
| Climate Change Awareness 2 (T1C, 2023, NC) | 0.14 | 0.09 |  |  |  |
| Climate Change Awareness 2 (T2A, 2023, NC) | 0.22 | 0.15 |  |  |  |
| Climate Change Awareness 2 (T2B, 2023, NC) | 0.17 | 0.06 | 0.06 |  |  |
| Climate Change Awareness 2 (T2C, 2023, NC) | 0.20 | 0.12 |  |  |  |
| Climate Change Awareness 2 (T3A, 2023, NC) | 0.23 | 0.17 |  |  | 0.28 |
| Climate Change Awareness 2 (T3B, 2023, NC) | 0.17 | 0.07 | 0.06 |  |  |
| Climate Change Awareness 2 (T3C, 2023, NC) | 0.19 | 0.13 |  |  |  |
| Climate Change Awareness 2 (T4A, 2023, NC) | 0.20 | 0.12 |  |  |  |
| Climate Change Awareness 2 (T4B, 2023, NC) | 0.10 | 0.02 | 0.05 |  |  |
| Climate Change Awareness 2 (T4C, 2023, NC) | 0.15 | 0.09 |  |  |  |
| Climate Consensus (A, 2021, MI) | 0.11 | 0.06 | 0.02 | 0.05 |  |
| Climate Consensus (B, 2022, NC) | 0.18 | 0.07 | 0.05 |  |  |
| Climate Consensus (C, 2023, GA) | 0.03 |  | 0.01 |  |  |
| Climate Consensus (D, 2022, MI) | 0.03 |  |  |  |  |
| Climate Consensus (E, 2022, NC) | 0.17 | 0.08 | 0.03 |  |  |
| Climate Consensus (F, 2022, NC) | 0.22 | 0.13 | 0.09 |  | 0.17 |
| Election Integrity (A, 2022, NC) | NA | 0.03 | NA |  |  |
| Election Integrity (A, 2023, AZ) | 0.06 | 0.07 |  |  |  |
| Election Integrity (B, 2022, NC) | 0.01 |  |  |  |  |
| Election Integrity (B, 2023, AZ) | 0.11 | 0.06 | 0.02 |  |  |
| Election Integrity 1 (2022, PA) | 0.06 | 0.03 | 0.02 |  |  |
| Election Integrity 2 (2022, PA) | 0.16 | 0.14 | 0.01 | 0.02 | 0.10 |
| Election Integrity 3 (2022, PA) | 0.11 | 0.05 |  |  |  |
| Election Integrity 4 (2022, AZ) | 0.10 | 0.05 | 0.01 |  |  |
| Election Integrity 5 (2022, NC) | 0.01 |  |  |  |  |
| Media literacy 1 (A, 2023, WI) | 0.04 |  | 0.03 | 0.07 | 0.10 |
| Media literacy 1 (B, 2023, WI) | 0.16 | 0.12 |  | 0.04 | 0.09 |
| Media literacy 2 (A, 2023, WI) | 0.22 | 0.17 | 0.02 | 0.07 | 0.09 |
| Media literacy 3 (2024, MI) | 0.18 | 0.16 | 0.02 | 0.04 | 0.17 |
| Vaccine 1 (A, 2022, MI) | 0.05 | 0.13 | 0.05 |  |  |
| Vaccine 1 (B, 2022, OH) | 0.14 | 0.22 | 0.05 |  |  |
| Vaccine 2 (A, 2022, MI) | NA | 0.11 | NA |  |  |
| Vaccine 2 (B, 2022, MI) | NA | 0.07 | NA |  |  |
| Vaccine 3 (2022, MI) | 0.03 | 0.08 | 0.04 |  |  |

Note: This table presents the p-values from Chi-square tests comparing the age structure between treatment and control groups across various quasi-experiments. In parentheses, letters are used when the same assessment was utilized in more than one quasi-experiment: A if same assessment, B if different assessments were used; the year, and the state: M Michigan, OH Ohio, PA Pennsylvania, NC North Carolina, TN Tennessee, GA Georgia, WI Wisconsin. The lack of significant differences is highlighted with a grayed-out background. The first two columns assess the effectiveness of randomization by comparing the age distributions of users reached by the intervention and those reached by the control group assessment (1st column) and users reached by the post-test assessment in both groups (2nd). The third and fourth columns test for self-selection bias by comparing the age distributions in the treatment group: users reached by the intervention and those reached by post-test assessment (3rd), as well as those who were reached by and those who engaged with the treatment post-test (4th). The fifth column evaluates the age distribution differences between users who engaged with the treatment assessment and those in the control group. NA in the table indicates that the demographic data necessary to perform the test were not available.

**S1 Table 5. Cramér’s V values for all pairwise comparisons by age, presented only for cases where the chi-squared test indicated statistical significance (**adjusted p < 0.05**).**

| Label | [R1] Reach of Treatment Video Ad (TG) vs Reach of Post-test (CG) | [R2] Reach of Post-test in TG vs in CG | [S1] Reach of Treatment Video Ad (TG) vs Reach of Post-test (TG) | [S2] Reach of Post-test (TG) vs Engagement of Post-test (TG) | [S3] Post-test Engagement in TG vs CG |
| --- | --- | --- | --- | --- | --- |
| Clean Energy (A, 2023, NC) | NA | 0.19 | NA |  | 0.22 |
| Clean Energy (B, 2023, NC) | NA | 0.19 | NA | 0.09 | 0.32 |
| Clean Energy (T1A, 2023, NC) | NA | 0.14 | NA |  |  |
| Clean Energy (T1B, 2023, NC) | NA | 0.20 | NA |  | 0.31 |
| Clean Energy (T1C, 2023, NC) | NA | 0.18 | NA |  | 0.35 |
| Clean Energy (T2A, 2023, NC) | NA | 0.12 | NA |  | 0.22 |
| Clean Energy (T2B, 2023, NC) | NA | 0.21 | NA |  | 0.32 |
| Clean Energy (T2C, 2023, NC) | NA | 0.18 | NA | 0.07 | 0.49 |
| Clean Energy (T3A, 2023, NC) | NA | 0.16 | NA |  | 0.30 |
| Clean Energy (T3B, 2023, NC) | NA | 0.26 | NA |  | 0.48 |
| Clean Energy (T3C, 2023, NC) | NA | 0.21 | NA |  | 0.36 |
| Clean Energy (T4A, 2023, NC) | NA | 0.13 | NA |  | 0.32 |
| Clean Energy (T4B, 2023, NC) | NA | 0.24 | NA |  | 0.47 |
| Clean Energy (T4C, 2023, NC) | NA | 0.18 | NA |  | 0.43 |
| Clean Energy 1 (A, 2022, NC) | 0.52 | 0.35 | 0.16 |  | 0.48 |
| Clean Energy 1 (B, 2022, NC) | 0.59 | 0.30 | 0.19 |  | 0.35 |
| Clean Energy 2 (A, 2022, TN) | 0.28 | 0.30 | 0.04 |  | 0.30 |
| Clean Energy 2 (B, 2022, TN) | 0.29 | 0.28 | 0.03 |  | 0.32 |
| Clean Energy 2 (C, 2022, TN) | 0.33 | 0.37 | 0.03 | 0.05 | 0.31 |
| Clean Energy 2 (D, 2022, TN) | 0.30 | 0.33 | 0.04 |  | 0.38 |
| Clean Energy 3 (A, 2022, TN) | 0.41 | 0.38 | 0.02 |  | 0.32 |
| Clean Energy 3 (B, 2022, TN) | 0.34 | 0.28 |  |  | 0.41 |
| Clean Energy 3 (C, 2022, TN) | 0.42 | 0.38 | 0.03 |  | 0.39 |
| Clean Energy 3 (D, 2022, TN) | 0.34 | 0.33 | 0.03 |  | 0.33 |
| Clean Energy 4 (A, 2022, TN) | 0.33 | 0.32 | 0.03 |  | 0.29 |
| Clean Energy 4 (B, 2022, TN) | 0.38 | 0.37 | 0.02 | 0.04 | 0.33 |
| Climate Change Awareness 1 (T1A, 2023, NC) | 0.07 | 0.10 | 0.06 |  | 0.26 |
| Climate Change Awareness 1 (T1B, 2023, NC) | 0.16 | 0.19 | 0.07 |  | 0.32 |
| Climate Change Awareness 1 (T1C, 2023, NC) | 0.17 | 0.18 | 0.05 |  | 0.29 |
| Climate Change Awareness 1 (T2A, 2023, NC) | 0.06 | 0.11 | 0.09 |  | 0.36 |
| Climate Change Awareness 1 (T2B, 2023, NC) | 0.18 | 0.22 | 0.11 |  | 0.31 |
| Climate Change Awareness 1 (T2C, 2023, NC) | 0.15 | 0.21 | 0.11 |  | 0.34 |
| Climate Change Awareness 1 (T3A, 2023, NC) | 0.10 | 0.16 | 0.10 |  | 0.34 |
| Climate Change Awareness 1 (T3B, 2023, NC) | 0.25 | 0.31 | 0.10 |  | 0.39 |
| Climate Change Awareness 1 (T3C, 2023, NC) | 0.18 | 0.25 | 0.11 |  | 0.50 |
| Climate Change Awareness 1 (T4A, 2023, NC) | 0.07 | 0.11 | 0.07 |  |  |
| Climate Change Awareness 1 (T4B, 2023, NC) | 0.14 | 0.14 | 0.08 |  | 0.24 |
| Climate Change Awareness 1 (T4C, 2023, NC) | 0.16 | 0.17 | 0.07 |  | 0.35 |
| Climate Change Awareness 2 (T1A, 2023, NC) | 0.36 | 0.13 | 0.17 | 0.07 | 0.35 |
| Climate Change Awareness 2 (T1B, 2023, NC) | 0.48 | 0.21 | 0.20 |  | 0.38 |
| Climate Change Awareness 2 (T1C, 2023, NC) | 0.47 | 0.19 | 0.20 | 0.07 | 0.42 |
| Climate Change Awareness 2 (T2A, 2023, NC) | 0.36 | 0.13 | 0.15 | 0.08 | 0.38 |
| Climate Change Awareness 2 (T2B, 2023, NC) | 0.47 | 0.20 | 0.16 |  | 0.33 |
| Climate Change Awareness 2 (T2C, 2023, NC) | 0.49 | 0.22 | 0.17 |  | 0.51 |
| Climate Change Awareness 2 (T3A, 2023, NC) | 0.39 | 0.19 | 0.13 | 0.07 | 0.38 |
| Climate Change Awareness 2 (T3B, 2023, NC) | 0.55 | 0.32 | 0.16 |  | 0.50 |
| Climate Change Awareness 2 (T3C, 2023, NC) | 0.48 | 0.26 | 0.14 | 0.08 | 0.52 |
| Climate Change Awareness 2 (T4A, 2023, NC) | 0.33 | 0.11 | 0.13 |  | 0.31 |
| Climate Change Awareness 2 (T4B, 2023, NC) | 0.42 | 0.16 | 0.15 |  | 0.34 |
| Climate Change Awareness 2 (T4C, 2023, NC) | 0.44 | 0.19 | 0.14 | 0.07 | 0.50 |
| Climate Consensus (A, 2021, MI) | 0.20 | 0.11 | 0.04 | 0.08 |  |
| Climate Consensus (B, 2022, NC) | 0.56 | 0.12 | 0.30 | 0.05 | 0.26 |
| Climate Consensus (C, 2023, GA) | 0.12 | 0.05 | 0.07 | 0.05 | 0.08 |
| Climate Consensus (D, 2022, MI) | 0.18 | 0.15 | 0.05 | 0.07 |  |
| Climate Consensus (E, 2022, NC) | 0.66 | 0.24 | 0.31 |  | 0.25 |
| Climate Consensus (F, 2022, NC) | 0.63 | 0.20 | 0.49 |  | 0.23 |
| Election Integrity (A, 2022, NC) | NA | 0.32 | NA | 0.05 | 0.36 |
| Election Integrity (A, 2023, AZ) | 0.15 | 0.03 | 0.14 |  |  |
| Election Integrity (B, 2022, NC) | 0.10 | 0.20 | 0.01 |  | 0.24 |
| Election Integrity (B, 2023) | 0.40 | 0.27 | 0.06 | 0.18 | 0.50 |
| Election Integrity 1 (2022) | 0.35 | 0.31 | 0.03 | 0.08 | 0.57 |
| Election Integrity 2 (2022) | 0.32 | 0.33 | 0.03 | 0.09 | 0.38 |
| Election Integrity 3 (2022) | 0.37 | 0.23 | 0.02 | 0.09 | 0.40 |
| Election Integrity 4 (2022) | 0.35 | 0.25 | 0.04 | 0.17 | 0.53 |
| Election Integrity 5 (2022) | 0.12 | 0.29 | 0.01 |  | 0.31 |
| Media literacy 1 (A, 2023) | 0.42 | 0.31 | 0.03 | 0.17 | 0.50 |
| Media literacy 1 (B, 2023) | 0.48 | 0.39 | 0.05 | 0.12 | 0.57 |
| Media literacy 2 (A, 2023) | 0.44 | 0.39 | 0.04 | 0.15 | 0.46 |
| Media literacy 3 (2024, MI) | 0.55 | 0.46 | 0.05 | 0.15 | 0.60 |
| Vaccine 1 (A, 2022, MI) | 0.18 | 0.23 | 0.03 |  | 0.20 |
| Vaccine 1 (B, 2022, OH) | 0.03 | 0.12 | 0.03 |  |  |
| Vaccine 2 (A, 2022, MI) | NA | 0.21 | NA |  | 0.21 |
| Vaccine 2 (B, 2022, MI) | NA | 0.21 | NA |  |  |
| Vaccine 3 (2022, MI) | 0.22 | 0.23 |  | 0.07 | 0.18 |

Note: This table presents the p-values from Chi-square tests comparing the age structure between treatment and control groups across various quasi-experiments. In parentheses, letters are used when the same assessment was utilized in more than one quasi-experiment: A if same assessment, B if different assessments were used; the year, and the state: M Michigan, OH Ohio, PA Pennsylvania, NC North Carolina, TN Tennessee, GA Georgia, WI Wisconsin. The lack of significant differences is highlighted with a grayed-out background. The first two columns assess the effectiveness of randomization by comparing the age distributions of users reached by the intervention and those reached by the control group assessment (1st column) and users reached by the post-test assessment in both groups (2nd). The third and fourth columns test for self-selection bias by comparing the age distributions in the treatment group: users reached by the quasi-experimental intervention and those reached by post-test assessment (3rd), as well as those were reached by and those who engaged with the treatment post-test (4th). The fifth column evaluates the age distribution differences between users who engaged with the treatment assessment and those in the control group. NA in the table indicates that the demographic data necessary to perform the test were not available.

# S1 Section 5. Outcome Measures

In all the analyzed quasi-experiments, we measured binary outcomes. This binary format was necessary due to the structure of Instagram ads, which allowed only two alternatives to be presented and recorded in polls. Most outcomes (n = 41) evaluated whether the intervention's message was reflected in participants' belief accuracy through true or false statements. For example, participants responded to statements like "98% of scientists believe that climate change is real." Some outcomes (n = 7) presented participants with two alternative answers, among which one was correct. For instance, participants might choose between two statements about a specific fact, identifying the correct answer after viewing the intervention video. Additionally, some outcomes measured participants' attitudes toward the tested issues. We assessed whether seeing our intervention increased the likelihood of presenting a pro-social attitude, such as supporting government investment in green energy or trust in the democratic process. Examples include questions like "Should the government invest in renewable energy?" or "Do you trust election workers?” Lastly, a few quasi-experiments tested behavioral intentions, asking the users if they intend to get vaccinated against COVID-19 or to vote in the 2022 midterm elections. These outcome measurements, however, suffer from unclear or incorrect question wording, including one which asked about a specific past behavior rather than a behavioral intention. Table 2 presents the list and frequency of all outcomes used in the quasi-experiments as well as the correct answer. In italics, we indicate the questions that were incorrectly or unclearly worded and did not generate statistically significant effects.

**S1 Table 6. Outcome measures used in the post-assessment quizzes.**

| **Question** | **N** | **True answer** | **False Answer** | **Type of outcome** |
| --- | --- | --- | --- | --- |
| Should the government invest in clean energy solutions? | 4 | Yes | No | Attitude |
| North Carolina is FIRST in the nation for rural clean energy jobs. | 2 | True | False | Belief Accuracy |
| Electric vehicles are greener than gas-cars- including electricity and batteries. | 4 | True | False | Belief Accuracy |
| Many Electric Vehicles cost less each month than gas | 4 | True | False | Belief Accuracy |
| More than 20,000 Tennesseans work for Electric Vehicle manufacturers making more than $ 20/hr | 2 | True | False | Belief Accuracy |
| *The climate is changing at exactly the pace that we are burning fossil fuels.* | 3 | True | False | Belief Accuracy |
| The climate is now changing much faster than in prehistoric times | 3 | True | False | Belief Accuracy |
| Nearly all scientists agree it's real, and we cause it. | 6 | True | False | Belief Accuracy |
| *Will you vote?* | 1 | Definitely | Maybe | Behavioral intention |
| Do you trust our state's election workers? | 1 | Yes | No | Attitude |
| The 2020 Election was legitimate and secure | 3 | True | False | Belief Accuracy |
| *Have you voted yet?* | 1 | Yes | No | Behavioral intention |
| The evidence shows that the 2020 election was secure. | 1 | True | False | Belief Accuracy |
| Most Americans think Congressman who helped plan the Jan 6 attack on the Capitol should be removed from office | 1 | True | False | Belief Accuracy |
| 100,000 Pennsylvanians are election workers. | 1 | True | False | Belief Accuracy |
| The most common polling places in Arizona are School & Churches | 1 | True | False | Belief Accuracy |
| STATE officials set North Carolina's Abortion, Gun, and Election laws. | 1 | True | False | Belief Accuracy |
| Which headline is trying to manipulate us? | 2 | Baby Formula is linked to horrifying disease among helpless infants. Parents despair | Infants who receive formula may be at slightly higher risk of ear infections. | Media Literacy |
| Which of these is trying to distract us from the issue by using insults? | 1 | You're wrong because you're too dumb to understand | You're wrong because you don't understand the issue | Media Literacy |
| Which of these is trying to trick us into choosing an extreme view? | 1 | Gun control is just politicians taking away our freedom | Gun laws are one part of making cities safer | Media Literacy |
| Did you, or do you want to get a COVID vaccine? | 2 | Yes | No | Behavioral Intention |
| Almost all U.S. medical doctors are fully vaccinated against COVID-19 | 2 | True | False | Belief Accuracy |
| You're much more likely to get struck by lightning than a serious side effect from a COVID vaccine. | 1 | True | False | Belief Accuracy |

*Note.* N indicates the number of times the outcome was used in separate quasi-experiments. The items indicated in italics are those that generated null effects due to the faulty wording of the question or the response options. The last column indicates which type of outcome each question represents (i.e., attitude, belief accuracy, behavioral intention, or media literacy).

#

# S1 Section 6. Analyses and Full Models

## Section 6.1. Model Description

In order to assess treatment outcomes, we conducted a random-effects meta-analysis of chi-squared effects. Given our research questions and the significant heterogeneity of the observed effect sizes, we conducted a moderated meta-regression analysis to explore potential sources of variability with topic and outcome (belief accuracy, attitude, and behavioral intention) as moderators. All analyses were conducted using the metafor package (Viechtbauer, 2010).

The analysis was carried out using the log odds ratio as the outcome measure. A random-effects model was fitted to the data. The amount of heterogeneity (i.e., τ²) was estimated using the restricted maximum-likelihood estimator (Viechtbauer, 2005). In addition to the estimate of τ², the Q-test for heterogeneity (Cochran, 1954) and the I² statistic are reported. In case any amount of heterogeneity is detected (i.e., τ² > 0, regardless of the results of the Q-test), a prediction interval for the true outcomes is also provided. Studentized residuals and Cook's distances are used to examine whether studies may be outliers and/or influential in the context of the model.

A total of k = 48 studies were included in the analysis. The observed log odds ratios ranged from -0.49 to 2.33, with the majority of estimates being positive (96%). The estimated average log odds ratio based on the random-effects model was μ ​ =1.04 (95% CI [0.85, 1.22]). This indicates that the average outcome differed significantly from zero, z = 10.72, *p* < .0001. According to the Q-test, the true outcomes appear to be heterogeneous, Q(47)=576.19, *p* <.0001,τ2=0.40, I2=91.71% . A 95% prediction interval for the true outcomes ranged from -0.22 to 2.29.

## Section 6.2. Model Results

**S1 Table 7.** Correct and false responses in all assessments included in the meta-analysis.

| Study | Control | | Treatment | | Chi2 | *p* |
| --- | --- | --- | --- | --- | --- | --- |
|  | n Correct | n False | n Correct | n False |  |  |
| Clean Energy (A. 2023. NC) | 162 | 133 | 161 | 92 | 3.93 | .048 |
| Clean Energy (T1A. 2023. NC) | 25 | 188 | 16 | 44 | 7.05 | .008 |
| Clean Energy (T1B. 2023. NC) | 35 | 108 | 36 | 38 | 11.87 | .001 |
| Clean Energy (T1C. 2023. NC) | 45 | 243 | 28 | 50 | 14.55 | .000 |
| Election Integrity (A. 2022. NC) | 45 | 172 | 29 | 181 | 3.11 | .078 |
| Election Integrity 3 (2022. PA) | 254 | 1785 | 264 | 241 | 393.34 | .000 |
| Vaccine 2 (A. 2022. MI) | 85 | 203 | 103 | 98 | 22.71 | .000 |
| Vaccine 2 (B. 2022. MI) | 70 | 165 | 86 | 75 | 21.36 | .000 |
| Clean Energy 1 (A. 2022. NC) | 45 | 195 | 79 | 56 | 59.96 | .000 |
| Clean Energy 1 (B. 2022. NC) | 94 | 533 | 68 | 135 | 32.26 | .000 |
| Clean Energy 2 (A. 2022. TN) | 81 | 275 | 52 | 105 | 5.57 | .018 |
| Clean Energy 2 (B. 2022. TN) | 66 | 285 | 48 | 87 | 14.32 | .000 |
| Clean Energy 2 (C. 2022. TN) | 75 | 238 | 83 | 133 | 12.08 | .001 |
| Clean Energy 2 (D. 2022. TN) | 40 | 279 | 70 | 81 | 63.51 | .000 |
| Clean Energy 3 (A. 2022. TN) | 86 | 232 | 42 | 75 | 2.82 | .093 |
| Clean Energy 3 (B. 2022. TN) | 59 | 247 | 26 | 51 | 6.66 | .010 |
| Clean Energy 3 (C. 2022. TN) | 95 | 242 | 30 | 68 | 0.12 | .734 |
| Clean Energy 3 (D. 2022. TN) | 48 | 232 | 42 | 64 | 20.49 | .000 |
| Clean Energy 4 (A. 2022. TN) | 140 | 504 | 87 | 114 | 35.10 | .000 |
| Clean Energy 4 (B. 2022. TN) | 137 | 653 | 76 | 119 | 41.92 | .000 |
| Climate Awareness 1 (T1A. 2023. NC) | 35 | 203 | 15 | 67 | 0.35 | .552 |
| Climate Awareness 1 (T1B. 2023. NC) | 60 | 155 | 19 | 49 | 0.00 | 1.000 |
| Climate Awareness 1 (T1C. 2023. NC) | 36 | 203 | 12 | 59 | 0.04 | .850 |
| Climate Awareness 2 (T1A. 2023. NC) | 48 | 204 | 37 | 57 | 14.17 | .000 |
| Climate Awareness 2 (T1B. 2023. NC) | 85 | 145 | 57 | 37 | 14.25 | .000 |
| Climate Awareness 2 (T1C. 2023. NC) | 65 | 225 | 44 | 68 | 10.80 | .001 |
| Climate Consensus (A. 2021. MI) | 408 | 632 | 174 | 109 | 43.81 | .000 |
| Climate Consensus (B. 2022. NC) | 353 | 651 | 112 | 88 | 29.68 | .000 |
| Climate Consensus (C. 2023. GA) | 95 | 239 | 190 | 340 | 4.75 | .029 |
| Climate Consensus (D. 2022. MI) | 79 | 127 | 72 | 38 | 20.04 | .000 |
| Climate Consensus (E. 2022. NC) | 79 | 586 | 78 | 144 | 60.20 | .000 |
| Climate Consensus (F. 2022. NC) | 30 | 244 | 251 | 217 | 132.01 | .000 |
| Election Integrity (A. 2023. AZ) | 79 | 189 | 70 | 184 | 0.15 | .698 |
| Election Integrity (B. 2022. NC) | 73 | 151 | 100 | 152 | 2.28 | .131 |
| Election Integrity (B. 2023. AZ) | 45 | 284 | 101 | 101 | 81.02 | .000 |
| Election Integrity 1 (2022. PA) | 112 | 564 | 154 | 93 | 182.60 | .000 |
| Election Integrity 1 (A. 2022. PA) | 81 | 216 | 55 | 29 | 39.99 | .000 |
| Election Integrity 2 (2022. PA) | 338 | 1735 | 1094 | 1105 | 534.12 | .000 |
| Election Integrity 2 (A. 2022. PA) | 55 | 198 | 152 | 53 | 123.46 | .000 |
| Election Integrity 4 (2022. AZ) | 162 | 715 | 151 | 73 | 207.63 | .000 |
| Election Integrity 5 (2022. NC) | 63 | 158 | 117 | 161 | 9.27 | .002 |
| Media literacy 1 (2023. W) | 30 | 208 | 57 | 87 | 35.61 | .000 |
| Media literacy 2 (2023. W) | 358 | 301 | 197 | 85 | 19.06 | .000 |
| Media literacy 3 (2023. W) | 49 | 327 | 135 | 120 | 115.24 | .000 |
| Media literacy 4 (2024. BA) | 284 | 340 | 202 | 162 | 8.77 | .003 |
| Vaccine 1 (A. 2022. BA) | 63 | 141 | 136 | 84 | 39.44 | .000 |
| Vaccine 1 (B. 2022. OH) | 86 | 176 | 116 | 54 | 50.52 | .000 |
| Vaccine 3 (A. 2022. BA) | 99 | 174 | 119 | 82 | 23.61 | .000 |

*Note.* The labels indicate the topic and version of the post-test assessment. In parentheses, letters are used when the same assessment was utilized in more than one experiment: A if same assessment, B if different assessments were used; the year, and the state: M Michigan, OH Ohio, PA Pennsylvania, NC North Carolina, TN Tennessee, GA Georgia, WI Wisconsin. Letters are used when the same assessment was utilized in more than one experiment. T1 to T4 indicates experiments with delayed measurements. The year the experiment was conducted, the state in which the experiment took place

## Section 6.3. Analyses by Topic

The test of subgroup homogeneity revealed that the Chi-square (Q statistic) was 3.15 with 3 degrees of freedom, which was not statistically significant (p = .37), indicating that there is no significant difference in effect sizes across the examined subgroups.

**S1 Table 8.** Heterogeneity measures by topic of the assessment

| Topic | τ² | H² | I² |
| --- | --- | --- | --- |
| Climate Change | 0.212 | 5.523 | 81.9 |
| Election Integrity | 0.972 | 39.356 | 97.5 |
| Media literacy | 0.531 | 18.392 | 94.6 |
| COVID-19 Vaccines | 0.019 | 1.477 | 32.3 |
| Overall | 0.398 | 12.060 | 91.7 |

*Note:* The heterogeneity measures indicate varying degrees of heterogeneity across the different subgroups, with the Election Integrity subgroup showing the highest level of heterogeneity and the COVID-19 Vaccine subgroup showing the lowest.

## Section 6.4. Analyses by outcome

The test of subgroup homogeneity revealed that the Chi-square (Q statistic) was 3.401 with 3 degrees of freedom, which was not statistically significant (p = .334), indicating that there is no significant difference in effect sizes across the examined subgroups.

**S1 Table 9.** Heterogeneity measures by outcome of the assessment

| Topic | τ² | H² | I² |
| --- | --- | --- | --- |
| Attitudes | .38 | 9.45 | 89.4% |
| Behavioral Intention | .41 | 10.09 | 90.1% |
| Belief Accuracy | .38 | 11.40 | 91.2% |
| Media Literacy | .53 | 18.39 | 94.6% |
| Overall | 0.398 | 12.060 | 91.7 |

## Section 6.5. Analyses Over-Time

We ran nine quasi-experiments on climate change measuring outcomes at four time points: 6, 80, 154, and 229 days after the start of the treatment advertisement. The targeted populations came from 3 geographical locations in North Carolina: Coastal Plains Rural (Place 1), Mountains Rural (Place 2), and Piedmont Rural (Place 3). We did not find any statistical differences between the targeted populations, hence we aggregated the results and analyzed them together in the main manuscript. The individual assessments targeted at three audiences in North Carolina and their sums at different time points, along with the 2x2 chi-square statistics, are presented in Table 10.

Quasi-experiment 1 assessed whether users correctly answered that “The climate is changing at exactly the pace that we are burning fossil fuels.” This quasi-experiment yielded no significant results across the three geographical locations because of a faulty premise of the outcome (i.e., the pace of change cannot be determined to be “exactly the came” and the rate at which the climate is changing is influenced by multiple factors in addition to fossil fuel consumption, such as deforestation, industrial processes, and agricultural practices, etc.; see Figure 7). Quasi-experiment 2 tested differences between treatment and control groups on responding to the statement, “The climate is now changing much faster than in prehistoric times.” Overall, at all time points, people who saw the treatment ad were more likely to answer correctly (i.e. “true”) to the given question (Figure 8). Lastly, in Quasi-experiment 3, which targeted people who already saw the previous two, found that the users had a significantly higher chance of responding 'yes' to the question “Should the government invest in clean energy solutions?” than the control group (Figure 9).

**S1 Fig 7.** Effect sizes of assessments of Quasi-experiment 1 at 4 measurement points.


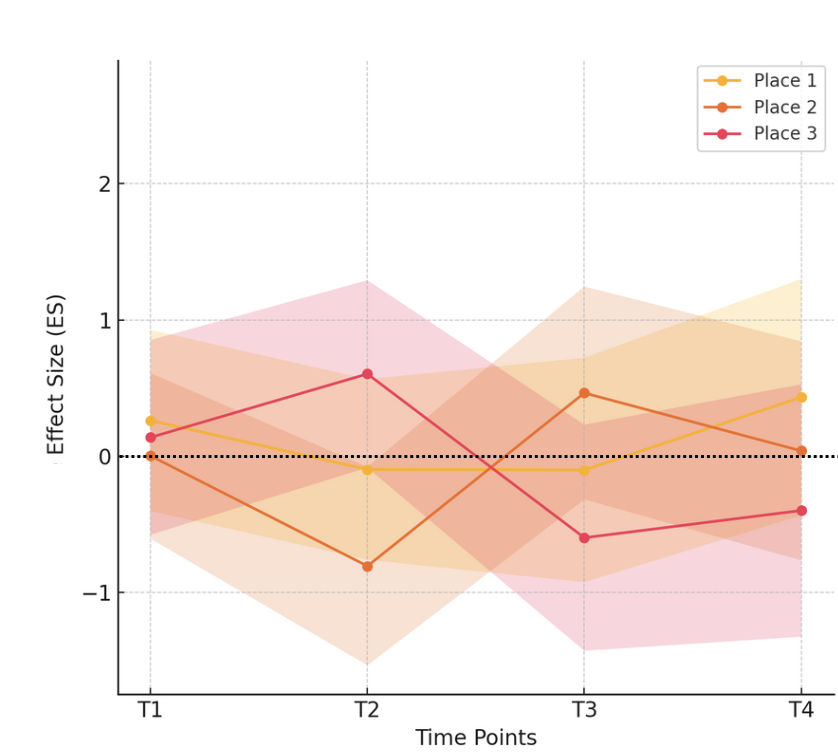


*Note.* Effect sizes of treatment targeted at audiences in 3 areas of North Carolina. Coastal Plains Rural (Place 1), Mountains Rural (Place 2), and Piedmont Rural (Place 3). Effect sizes are expressed as Log Odds Ratios. T1 measurement 6 days after the start of the treatment advertisement, T2 measurement 80 days, T3 measurement 153 days, and T4 measurement 229 days.

**S1 Fig 8.** Effect sizes of assessments of Quasi-experiment 2 at 4 measurement points.
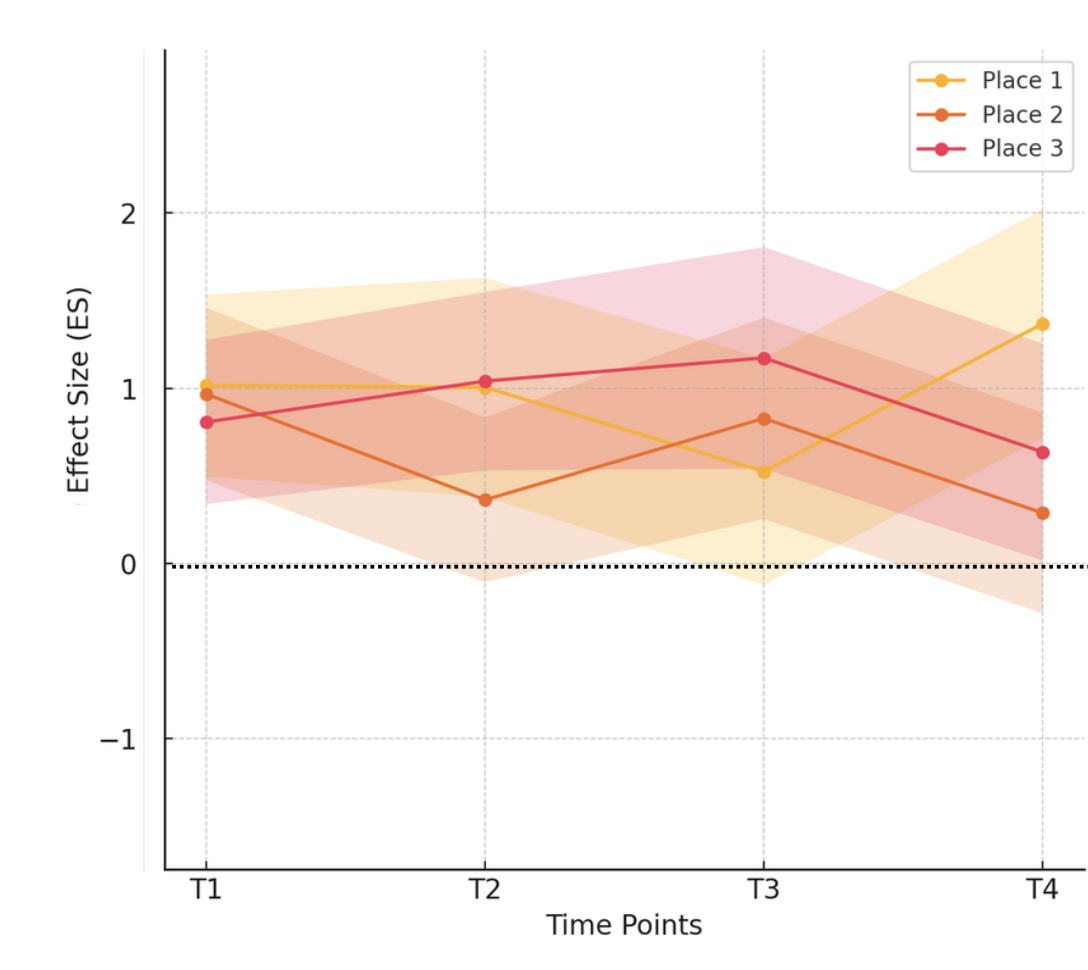


*Note.* Effect sizes of treatment targeted at audiences in 3 areas of North Carolina. Coastal Plains Rural (Place 1), Mountains Rural (Place 2), and Piedmont Rural (Place 3). Effect sizes are expressed as Log Odds Ratios. T1 measurement 6 days after the start of the treatment advertisement, T2 measurement 80 days, T3 measurement 153 days, and T4 measurement 229 days.

**S1 Fig 9.** Effect sizes of assessments of Quasi-experiment 2 at 4 measurement points.


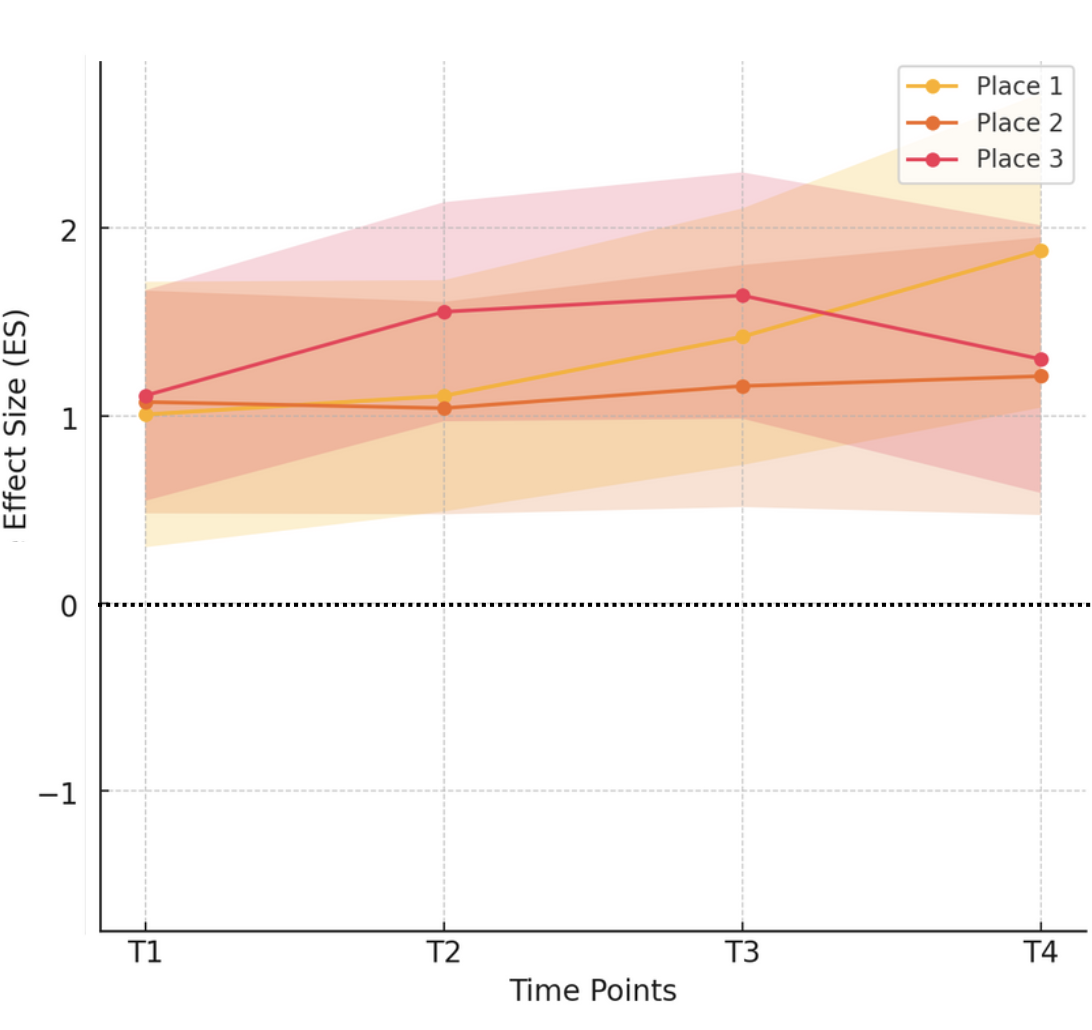


*Note.* Effect sizes of treatment targeted at audiences in 3 areas of North Carolina. Coastal Plains Rural (Place 1), Mountains Rural (Place 2), and Piedmont Rural (Place 3). Effect sizes are expressed as Log Odds Ratios. T1 measurement 6 days after the start of the treatment advertisement, T2 measurement 80 days, T3 measurement 153 days, and T4 measurement 229 days.

**S1 Table 10.** Correct and false responses in all assessments included in the delayed measurement analysis.

| Study | Place | Control | | Treatment | | Chi2 | *p* |
| --- | --- | --- | --- | --- | --- | --- | --- |
|  |  | N Correct | N False | N Correct | N False |  |  |
| Climate Change Awareness 1 (T1A. 2023. NC) | 1 | 35 | 203 | 15 | 67 | 0.354 | 0.552 |
| Climate Change Awareness 1 (T1B. 2023. NC) | 2 | 60 | 155 | 19 | 49 | 0.000 | 1.000 |
| Climate Change Awareness 1 (T1C. 2023. NC) | 3 | 36 | 203 | 12 | 59 | 0.036 | 0.850 |
| Climate Change Awareness 1 (T1. 2023. NC) | SUM | 131 | 561 | 46 | 175 | 0.269 | 0.604 |
| Climate Change Awareness 1 (T2A. 2023. NC) | 1 | 49 | 184 | 14 | 58 | 0.015 | 0.901 |
| Climate Change Awareness 1 (T2B. 2023. NC) | 2 | 65 | 171 | 10 | 59 | 4.225 | 0.040 |
| Climate Change Awareness 1 (T2C. 2023. NC) | 3 | 29 | 205 | 15 | 58 | 2.386 | 0.122 |
| Climate Change Awareness 1 (T2. 2023. NC) | SUM | 143 | 560 | 39 | 175 | 0.339 | 0.561 |
| Climate Change Awareness 1 (T3A. 2023. NC) | 1 | 26 | 133 | 9 | 51 | 0.001 | 0.971 |
| Climate Change Awareness 1 (T3B. 2023. NC) | 2 | 27 | 118 | 12 | 33 | 0.914 | 0.339 |
| Climate Change Awareness 1 (T3C. 2023. NC) | 3 | 34 | 133 | 8 | 57 | 1.539 | 0.215 |
| Climate Change Awareness 1 (T3. 2023. NC) | SUM | 87 | 384 | 29 | 141 | 0.086 | 0.769 |
| Climate Change Awareness 1 (T4A. 2023. NC) | 1 | 27 | 182 | 8 | 35 | 0.547 | 0.459 |
| Climate Change Awareness 1 (T4B. 2023. NC) | 2 | 79 | 228 | 9 | 25 | 0.000 | 1.000 |
| Climate Change Awareness 1 (T4C. 2023. NC) | 3 | 48 | 177 | 6 | 33 | 0.404 | 0.525 |
| Climate Change Awareness 1 (T4. 2023. NC) | SUM | 154 | 587 | 23 | 93 | 0.013 | 0.910 |
| Climate Change Awareness 2 (T1A. 2023. NC) | 1 | 48 | 204 | 37 | 57 | 14.169 | 0.000 |
| Climate Change Awareness 2 (T1B. 2023. NC) | 2 | 85 | 145 | 57 | 37 | 14.254 | 0.000 |
| Climate Change Awareness 2 (T1C. 2023. NC) | 3 | 65 | 225 | 44 | 68 | 10.800 | 0.001 |
| Climate Change Awareness 2 (T1. 2023. NC) | SUM | 198 | 574 | 138 | 162 | 40.645 | 0.000 |
| Climate Change Awareness 2 (T2A. 2023. NC) | 1 | 39 | 213 | 21 | 42 | 9.297 | 0.002 |
| Climate Change Awareness 2 (T2B. 2023. NC) | 2 | 90 | 158 | 45 | 55 | 1.925 | 0.165 |
| Climate Change Awareness 2 (T2C. 2023. NC) | 3 | 51 | 234 | 37 | 60 | 15.615 | 0.000 |
| Climate Change Awareness 2 (T2. 2023. NC) | SUM | 180 | 605 | 103 | 157 | 26.697 | 0.000 |
| Climate Change Awareness 2 (T3A. 2023. NC) | 1 | 27 | 116 | 22 | 56 | 2.031 | 0.154 |
| Climate Change Awareness 2 (T3B. 2023. NC) | 2 | 45 | 95 | 39 | 36 | 7.277 | 0.007 |
| Climate Change Awareness 2 (T3C. 2023. NC) | 3 | 26 | 132 | 28 | 44 | 12.634 | 0.000 |
| Climate Change Awareness 2 (T3. 2023. NC) | SUM | 98 | 343 | 89 | 136 | 21.316 | 0.000 |
| Climate Change Awareness 2 (T4A. 2023. NC) | 1 | 41 | 379 | 17 | 40 | 17.082 | 0.000 |
| Climate Change Awareness 2 (T4B. 2023. NC) | 2 | 110 | 223 | 23 | 35 | 0.693 | 0.405 |
| Climate Change Awareness 2 (T4C. 2023. NC) | 3 | 40 | 169 | 21 | 47 | 3.465 | 0.063 |
| Climate Change Awareness 2 (T4. 2023. NC) | SUM | 191 | 771 | 61 | 122 | 15.498 | 0.000 |
| Clean Energy (T1A. 2023. NC) | 1 | 25 | 188 | 16 | 44 | 7.048 | 0.008 |
| Clean Energy (T1B. 2023. NC) | 2 | 35 | 108 | 36 | 38 | 11.870 | 0.001 |
| Clean Energy (T1C. 2023. NC) | 3 | 45 | 243 | 28 | 50 | 14.553 | 0.000 |
| Clean Energy (T1. 2023. NC) | SUM | 105 | 539 | 80 | 132 | 41.986 | 0.000 |
| Clean Energy (T2A. 2023. NC) | 1 | 41 | 291 | 20 | 47 | 11.868 | 0.001 |
| Clean Energy (T2B. 2023. NC) | 2 | 54 | 162 | 33 | 35 | 12.390 | 0.000 |
| Clean Energy (T2C. 2023. NC) | 3 | 43 | 276 | 28 | 38 | 28.567 | 0.000 |
| Clean Energy (T2. 2023. NC) | SUM | 138 | 729 | 81 | 120 | 58.019 | 0.000 |
| Clean Energy (T3A. 2023. NC) | 1 | 18 | 210 | 22 | 62 | 16.783 | 0.000 |
| Clean Energy (T3B. 2023. NC) | 2 | 39 | 150 | 24 | 29 | 11.811 | 0.001 |
| Clean Energy (T3C. 2023. NC) | 3 | 22 | 227 | 24 | 48 | 25.344 | 0.000 |
| Clean Energy (T3. 2023. NC) | SUM | 79 | 587 | 70 | 139 | 51.162 | 0.000 |
| Clean Energy (T4A. 2023. NC) | 1 | 12 | 189 | 15 | 36 | 20.980 | 0.000 |
| Clean Energy (T4B. 2023. NC) | 2 | 32 | 113 | 19 | 20 | 9.605 | 0.002 |
| Clean Energy (T4C. 2023. NC) | 3 | 23 | 169 | 18 | 36 | 12.343 | 0.000 |
| Clean Energy (T4. 2023. NC) | SUM | 67 | 471 | 52 | 92 | 42.511 | 0.000 |

*Note.* The labels indicate the topic and version of the post-test assessment. In parentheses, letters are used when the same assessment was utilized in more than one quasi-experiment: A if same assessment, B if different assessments were used; the year, and the state: M Michigan, OH Ohio, PA Pennsylvania, NC North Carolina, TN Tennessee, GA Georgia, WI Wisconsin. Letters are used when the same assessment was utilized in more than one quasi-experiment. T1 to T4 indicates quasi-experiments with delayed measurements. The year the quasi-experiment was conducted, the state in which the quasi-experiment took place

## Section 6.6. Analyses by main persuasive appeal

The **test of subgroup homogeneity** revealed a Chi-square (Q statistic) value of 13.645 with 4 degrees of freedom, which was statistically significant (p = .009). This indicates significant differences in effect sizes across the examined subgroups.

**S1 Table 11.** Heterogeneity measures by main persuasive strategy of the treatment video

| Content feature | τ² | H² | I² |
| --- | --- | --- | --- |
| Individual Benefits | 0.298 | 6.324 | 84.2% |
| Logical Inference | 0.044 | 1.603 | 37.6% |
| Other | 0.457 | 13.080 | 92.4% |
| Social Norms | 0.240 | 10.947 | 90.9% |
| Source Credibility | 0.551 | 19.072 | 94.8% |
| Overall | 0.397 | 11.984 | 91.7% |

**S1 Fig 10.** Average effect sizes of treatments by persuasive strategy


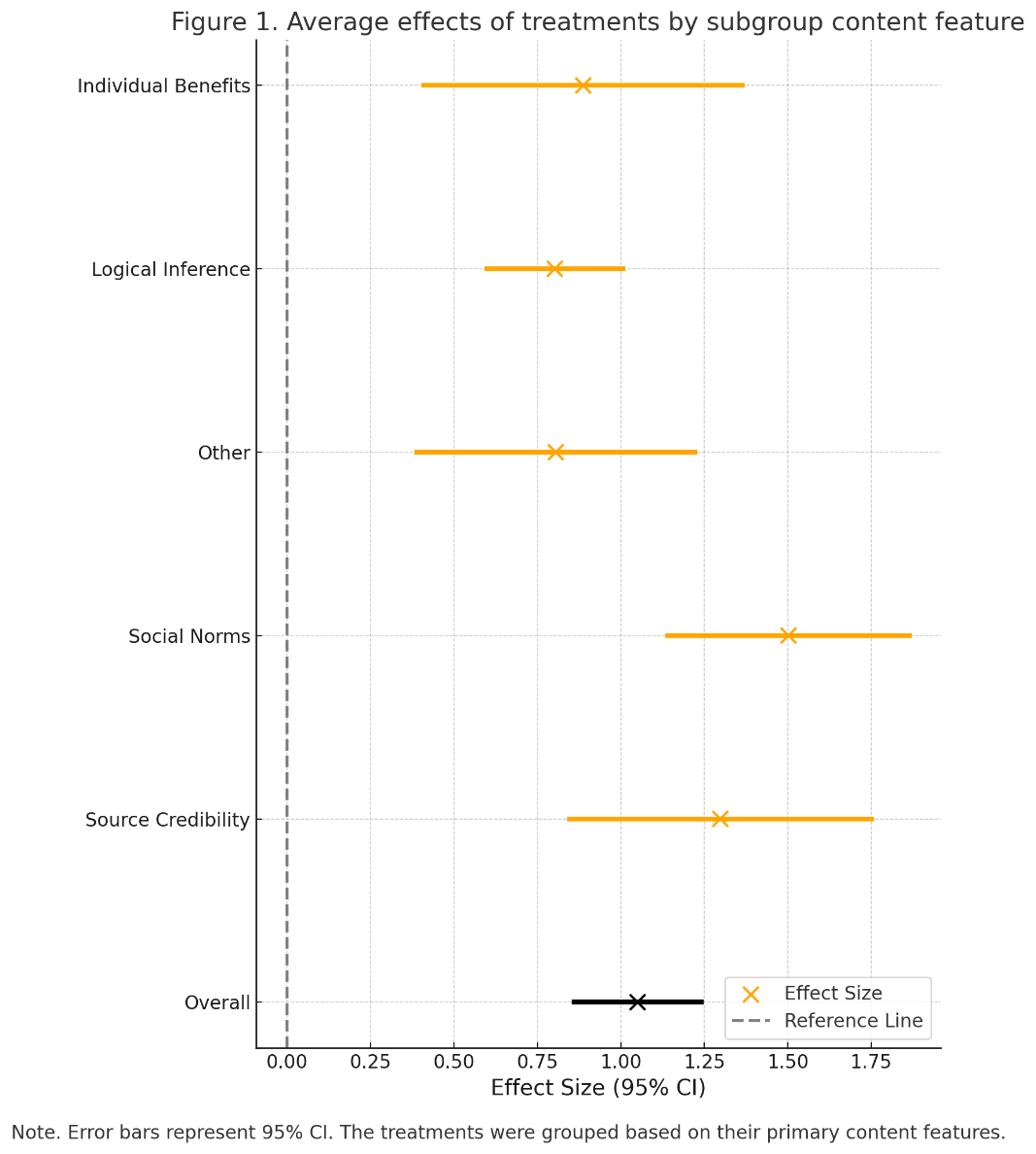


*Note.* Error bars represent 95% CI. We aggregate the treatments that utilize the same primary persuasive strategy

Descriptive comparisons of the average effect sizes indicated that the Logical Inference appeal produced an effect size of 0.802 (95% CI [0.597, 1.006]). The confidence intervals for Social Norms (95% CI [1.141, 1.864]) and Source Credibility (95% CI [0.846, 1.751]) were higher and did not fully overlap with that of Logical Inference, suggesting potentially larger effect sizes for these strategies. In contrast, the confidence intervals for Individual Benefits (95% CI [0.409, 1.364]) and Other (95% CI [0.388, 1.223]) overlapped with Logical Inference, indicating no clear difference at the descriptive level.

To statistically test whether these apparent differences in effect sizes were significant, we conducted a random-effects meta-regression using REML estimation. The model showed a significant overall moderation effect, Wald χ²(4) = 10.285, p = .036, indicating that persuasive strategy as a set of predictors explained some variability in effect sizes. However, none of the individual predictors reached statistical significance. The coefficient for Logical Inference approached significance (Estimate = –0.529, p = .058), suggesting a trend toward smaller effect sizes relative to the reference category, though not a reliable difference. Other predictors—Individual Benefits (Estimate = –0.412, p = .204), Other (Estimate = –0.493, p = .069), and Social Norms (Estimate = 0.206, p = .475)—were not significant.

Overall, while the descriptive patterns hint that Logical Inference may yield smaller effect sizes than Social Norms or Source Credibility, the meta-regression results indicate that these differences are not statistically robust. Substantial residual heterogeneity remains, implying that additional unmeasured study characteristics may account for variability in the observed results.

**Section 6.7. Analyses by modality**

In order to examine potential sources of variability in effect sizes, we conducted a random-effects meta-regression analysis using REML estimation. The test of subgroup homogeneity revealed a Chi-square (Q statistic) value of 4.060 with 3 degrees of freedom, which was not statistically significant (p = .255). This indicates that there are no significant differences in effect sizes across the examined subgroups.

**S1 Table 12.** Heterogeneity measures by modality of the treatment video

| Modality | τ² | H² | I² |
| --- | --- | --- | --- |
| Text and Drawings | 0.122 | 3.793 | 73.6% |
| Text and Informational Graphics | 0.173 | 7.912 | 87.4% |
| Text and Realistic Images | 0.418 | 13.795 | 92.8% |
| Text Only | 0.706 | 15.970 | 93.7% |
| Overall | 0.397 | 11.984 | 91.7% |

**S1 Fig 11.** Average effect sizes of treatments by modality


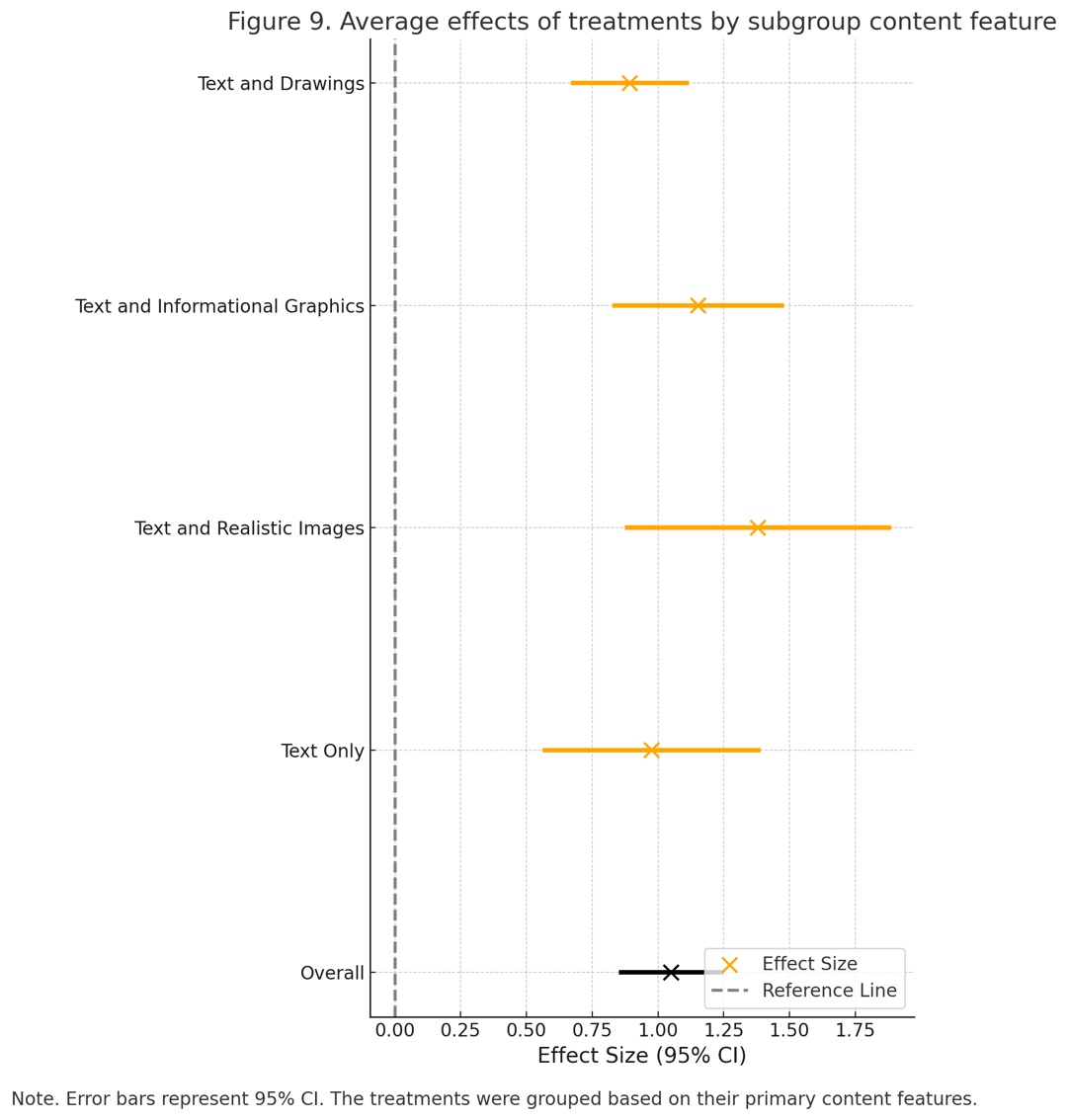


*Note.* Error bars represent 95% CI. We aggregate the treatment videos utilizing the same modality

Further analysis of the average effect sizes indicated that the effect size for Text and Drawings was 0.893 (95% CI [0.678, 1.108]), representing one of the content features utilized in the treatment conditions. Comparisons of Text and Drawings with other subgroups, including Text and Informational Graphics (95% CI [0.836, 1.470]) and Text and Realistic Images (95% CI [0.882, 1.879]), revealed overlapping confidence intervals, indicating that the differences were not statistically significant. Similarly, the confidence intervals for Text Only (95% CI [0.570, 1.381]) also overlapped with those of Text and Drawings, further supporting the lack of significant differences among these subgroups.

#

# References

Cialdini, R. B., Kallgren, C. A., & Reno, R. R. (1991). A focus theory of normative conduct: A theoretical refinement and reevaluation of the role of norms in human behavior. In *Advances in experimental social psychology* (Vol. 24, pp. 201-234). Academic Press.

Collier, J. R., Pillai, R. M., & Fazio, L. K. (2023). Multiple-choice quizzes improve memory for misinformation debunks, but do not reduce belief in misinformation. *Cognitive Research: Principles and Implications*, *8*. <https://doi.org/10.1186/s41235-023-00488-9>

Capewell, G., Maertens, R., Linden, S., Remshard, M., Compton, J., Lewandowsky, S., & Roozenbeek, J. (2023, July 24). Misinformation interventions decay rapidly without an immediate post-test. <https://doi.org/10.31234/osf.io/93ujx>

Dixon, G., McBAeever, B. W., Holton, A. E., Clarke, C., & Eosco, G. M. (2015). The power of a picture: overcoming scientific misinformation by communicating weight-of-evidence information with visual exemplars. *Journal of Communication, 65*(4), 639-659. <https://doi.org/10.1111/jcom.12159>

Gaudette, J., Hill, S., Kousser, T., Lockhart, M., & Romero, M. (2023, November). *Evaluating practitioner interventions to increase trust in elections*. Evaluating Practitioner Interventions to Increase Trust in Elections | MIT Election Lab. <https://electionlab.mit.edu/articles/evaluating-practitioner-interventions-increase-trust-elections>

Hamel, L. & Brodie, M. (Apr 16, 2021). KFF COVID-19 Vaccine Monitor: What We’ve Learned. Available at<https://www.kff.org/coronavirus-covid-19/poll-finding/kff-covid-19-vaccine-monitor-what-weve-learned-april-2021/>

Kozyreva, A., Lewandowsky, S., & Hertwig, R. (2020). Citizens versus the internet: confronting digital challenges with cognitive tools. *Psychological Science in the Public Interest, 21(*3), 103-156. <https://doi.org/10.1177/1529100620946707>

Lewandowsky, S., & van der Linden, S. (2021). Countering Misinformation and Fake News Through Inoculation and Prebunking. *European Review of Social Psychology, 32*(2), 348–384. <https://doi.org/10.1080/10463283.2021.1876983>

Li, J. (2024). Promoting HPV vaccination: effectiveness of mobile short videos for shaping attitudes and influencing behaviors. *Humanities and Social Sciences Communications*, *11*(1), 1-11.

Petty, R. E., & Cacioppo, J. T. (1986). The elaboration likelihood model of persuasion. *Advances in experimental social psychology*, *19*.

Pratelli, Manuel, Marinella Petrocchi, Fabio Saracco, and Rocco De Nicola. 2024. “Online Disinformation in the 2020 U.S. Election: Swing vs. Safe States.” *EPJ Data Science* 13(1):1–23. doi:[10.1140/epjds/s13688-024-00461-6](https://doi.org/10.1140/epjds/s13688-024-00461-6).

Robert, L. P., & Dennis, A. R. (2005). Paradox of richness: A cognitive model of media choice. *IEEE transactions on professional communication*, *48*(1), 10-21.

Roozenbeek, J., Goldberg, B., Rathje, S., & Lewandowsky, S. (2022). Psychological inoculation improves resilience against misinformation on social media. *Science Advances*. <https://doi.org/abo6254>

Sussman, S. W., & Siegal, W. S. (2003). Informational influence in organizations: An integrated approach to knowledge adoption. *Information systems research*, *14*(1), 47-65.

White, K., & Peloza, J. (2009). Self-benefit versus other-benefit marketing appeals: Their effectiveness in generating charitable support. *Journal of Marketing*, *73*(4), 109-124.

1. <https://youtu.be/gDfQHWQwJ8Q> [↑](#footnote-ref-1)
