# Supplementary material for: Ad-based social media interventions increase belief accuracy and generate pro-social opinions among non-news readers
Source: PLoS One. 2026 Jun 29;21(6):e0352588. doi: 10.1371/journal.pone.0352588 (PMC13313353; doi:10.1371/journal.pone.0352588)
Supplement: Attachment — Submitted filename: RR Memo.docx [file pone.0352588.s003.docx]

I have now received two reviews. Both reviewers were very positive about the paper’s overall quality, and I share their view that this is an ambitious, carefully executed, and potentially highly useful contribution. At the same time, some of the reviewers and I converged on a similar point: the main remaining issues concern framing, positioning, and interpretive precision, rather than the underlying analyses. In other words, the reviewers found the empirical core of the paper strong, but they felt the manuscript would benefit from a clearer articulation of exactly what its contribution is.

Thank you and both reviewers for your careful and constructive evaluations. We are grateful for the positive assessment of the manuscript’s empirical contribution and methodological rigor. We found the comments helpful and have revised the manuscript to respond to all the suggestions. Across the revision, our main goals were to:

(1) clarify and front-load the manuscript’s core contribution, namely to propose and test an intervention that presents more verified and factual information in the social media feeds of users who do not encounter much public affairs information at all;

(2) position that contribution more explicitly within existing literatures on news non-use, informational absence, and misinformation interventions;

(3) revisit and clarify language around behaviors versus behavioral intentions, as well as around belief accuracy and attitudes to more carefully bracket each;

(4) expand the discussion of boundary conditions and limitations.

Below, we respond point by point to each comment from the editor and from each reviewer and the final manuscript highlights the changes in blue.

Most importantly, the reviewers encourage you to front-load the paper’s central contribution more explicitly. As they read it, the manuscript’s main value is not simply to show that short factual ads can persuade people online, since persuasive effects of media interventions are already well established. Rather, the more distinctive contribution is that the paper reframes a problem often discussed as “misinformation” into one of informational absence among news non-users, and then demonstrates that this reframing can be operationalized at scale through existing advertising infrastructure. Making this point more clearly in the introduction and discussion would help align expectations with what the study actually shows and improve the paper’s visibility.

We have moved paragraphs talking about the importance of presenting verified and factual public affairs information to earlier in our introduction, foregrounding that our work seeks to reframe problems often thought of as caused by exposure to ‘bad’ information as those (more likely) caused by informational absence. Correspondingly, we have adjusted language throughout to emphasize these issues over misinformation.

Relatedly, one reviewer asks that this reframing be situated more explicitly within the relevant literature. The manuscript already cites work on news avoidance, limited exposure to verified information, and interventions aimed at misinformation, but the reviewers felt this could be woven into a more coherent intellectual lineage. This would clarify that the paper is not claiming to invent the broader insight from scratch, but rather to extend and empirically implement it in a novel and scalable way.

We have added language making our connection to the news avoidance literature—as well as the literature on existing prosocial interventions for misinformation, etc.—more explicit. To that end, we have added citations to two recent reviews of news avoidance literature to better highlight the research gap that this paper helps address.

I would also encourage somewhat more explicit discussion of the paper’s boundary conditions. In particular, because the intervention relies on advertising infrastructure that is also available to actors with competing or adversarial agendas, it would strengthen the manuscript to briefly acknowledge that the present studies occur in relatively non-confrontational informational environments. A short discussion of how effectiveness may differ in more competitive or adversarial advertising ecologies would improve the paper’s policy relevance without requiring new analyses.

Thank you for this feedback. We have expanded the paragraph in our limitations section (p. 18), which mentions how interventions such as ours could be weaponized. We also note that our current results cannot address what would occur in more contested information environments.

A further set of revisions concerns interpretive precision around the outcomes. One reviewer recommends being more careful with the term “belief accuracy,” noting that the current measures may capture short-term information uptake, accessibility, or response tendencies shortly after exposure rather than deeper or more durable belief revision. Along similar lines, it would help to maintain sharper distinctions among beliefs, attitudes, and behavioural intentions, especially where some items may be politically or normatively loaded, and to avoid language that implies demonstrated behavioural change when only intentions were measured.

As R1 suggests, we have added a new paragraph to our limitations section (p. 18) concerning the limited nature of our DVs, and adjusted language around behavioral change and behavioral intention to more carefully distinguish between them.

There are also a few smaller points worth addressing. The reviewers suggest that the limitations section would be strengthened by integrating these interpretive caveats more directly. It may also be useful to clarify that the longer-term persistence evidence is shown most clearly for the climate change outcomes, and to briefly note that effects may differ among audiences with stronger prior attitudes or greater issue engagement.

As above, we have adjusted our limitations section to make all of the above concerns more explicit, as indicated above. Additionally, we have modified language on p. 18 to more firmly emphasize that we only test long term effects with climate change outcomes.

Overall, however, the tenor of the reviews is highly encouraging. Both reviewers and I see this as a strong manuscript that already makes an important empirical contribution. The requested changes are primarily to help readers understand the paper on its own terms and to ensure that the claims are framed as precisely as the evidence allows.

I would therefore encourage you to revise the manuscript with these points in mind. I believe that a careful revision along these lines would substantially strengthen what is already a very promising paper.

Again, thank you for the positive response. We trust that via reframing our introduction and introducing more careful and explicit mentions of our boundary conditions and limitations we have substantially improved the manuscript to a state ready for publication.

In addition, we make sure to comply with each point of the journal and publication requirements listed in the decision email.

- We have adjusted our headings, figures and supporting information, including filenames, to match PLOS One’s guidelines.
- We have provided details regarding participant consent, noting in the ethics statement in the Methods and online submission information that there was no consent for this study.
- We have made sure that the funding information provided in the ‘Funding Information’ and ‘Financial Disclosure’ sections match. We only note that there are no specific grant numbers for the foundation awards that Reality Team received for their work.
- With regard to financial disclosure, you are correct that the funders had no role in the study. As such, the final section should read:

“The authors also gratefully acknowledge the support of the European Research Council (ERC Consolidator 101126218, NEWSUSE: Incentivizing Citizen Exposure to Quality News Online: Framework and Tools, PI Magdalena Wojcieszak) and of the Center for Excellence in Social Sciences at the University of Warsaw. The funders had no role in study design, data collection and analysis, decision to publish, or preparation of the manuscript."

- We have adjusted the Acknowledgments Section to not include any funding information. As noted above, we also update our Funding Statement to read:

“The authors also gratefully acknowledge the support of the European Research Council (ERC Consolidator 101126218, NEWSUSE: Incentivizing Citizen Exposure to Quality News Online: Framework and Tools, PI Magdalena Wojcieszak) and of the Center for Excellence in Social Sciences at the University of Warsaw.

- We have made sure that the ethics statement only appears in the Methods section of the final manuscript, and delete it from Supplementary material as requested.
- We have included an “other” file in the online submission system, where we detail the information regarding the figures in our manuscript and where we present written permission from the copyright holder to publish all figures (written and signed by the co-authors from Reality Team). All relevant S1 figures have added the relevant language.
- Regarding Figures SM2B and SM5, please know that these figures do not include study participants. Rather, they are stock photos used in the treatment materials, and, in one case, a meme featuring the actor Jack Nicholson. In particular, SM Figure 5 (the Dirty Disinfo Tricks), in panel A, those images were stock photos in Canva. For panel B, the Jack Nicholson meme was acquired from memes.com, a meme generator site. Our use complies with both Canva’s and memes.com’s policies regarding dissemination of this content.
- We were asked to submit an upscaled version of Figure 1. We include large versions of all figure images alongside our revised manuscript files in filetypes listed as acceptable on PLOS One’s guidelines.
- With regard to captions for our Supporting Information files, we rename SM to S1; and adjust all section labels accordingly; rather than splitting into several documents.
- As recommended, we have uploaded our dataset to a repository (OSF) and will begin reformatting and uploading all code and supporting documents at this time. We will be prepared to immediately make that repository public upon acceptance.
- Lastly, we review our reference list, correcting errors and adding previously missing references cited in text. In response to R1’s feedback, we have revised our literature review; adding several citations and removing others that are less relevant to the reframing requested by reviewers. All adjustments to the bibliography are marked in blue.

**Reviewers' comments:**

**Reviewer #1: Review of “Ad-Based Social Media Interventions Increase Belief Accuracy and Generate Pro-Social Opinions Among Non-News Readers”**

Summary

This manuscript reports a large set of field quasi-experiments testing whether short, factual Instagram advertisements can improve belief accuracy, attitudes, and behavioural intentions among news non-users. Using Meta’s advertising infrastructure, the authors demonstrate consistent positive effects on factual endorsement and some attitudinal outcomes across multiple issue domains (climate change, COVID-19 vaccines, election integrity, and media literacy), with no evidence of backfire and some evidence of persistence over time.

The study is ambitious in scale, carefully executed within the constraints of platform-based field experimentation, and transparent about its limitations. The work is well suited to PLOS One’s focus on methodological soundness and contribution through empirical validation. The main issues raised below concern framing, interpretation, and positioning of the contribution rather than the analyses themselves.

Thank you for noting that the study is ambitious, carefully executed, and transparent about its limitations. We greatly appreciate your positive assessment, as well as the thoughtful comments, all of which we incorporate.

Major Comments

1. Clarify the paper’s contribution as reframing rather than persuasion

The manuscript would benefit from more explicitly stating that its primary contribution is not demonstrating that advertising can persuade, an established finding, but rather reframing the broader problem often discussed under the banner of “misinformation” as one of informational absence among news non-users, and empirically showing that this reframing leads to scalable, non-backfiring intervention strategies using existing platform infrastructure.

At present, some parts of the manuscript risk being read as advancing a general claim about persuasive effectiveness of online videos, which invites evaluation criteria (e.g., depth of belief change, behavioural outcomes) that go beyond what the study is designed to address. Clearer signposting of the reframing as the central contribution would better align reader expectations with the paper’s actual strengths.

For example, the authors might consider language along the lines of:

“Rather than testing whether online advertising can persuade, a well-established result, this study demonstrates that reframing the problem as one of informational absence among news non-users yields viable, scalable interventions that improve factual endorsement and attitudes in real-world settings.”

Thank you for this comment. As you suggest, we have reorganized the front end of the article to first state that the broader societal problems often attributed to “bad” information online (e.g., biased, untrustworthy, misinformative) are likely due to the absence of “good” (factual and verified) public affairs information among social media users. As such, instead of implying that our contribution is the finding that advertising can persuade, we focus on making factual and verified information easily available in users’ feeds. In addition, we better foreground that our tested interventions public affairs information to those who are unlikely to encounter such information in their social media feeds. In particular, we have substantially rewritten the introduction and moved (and adjusted) one paragraph from later in the introduction to page 1 of the paper, emphasizing this aspect of our contribution. We have also adjusted language in the abstract, significance statement and the discussion (p. 19) to reflect this reemphasis. We agree that this reframing and re-emphasis better highlight our unique strengths and contributions.

2. Situate the reframing more explicitly within existing literature

Related to the above, the manuscript draws on several existing literatures that have already pointed toward aspects of this reframing, including work on news avoidance and non-use, the informational consequences of limited exposure to verified news, and the unintended effects of debunking- or skepticism-focused interventions. Much of this work is cited, but it is largely treated as background motivation rather than as part of a coherent intellectual lineage.

Making this lineage more explicit, by clarifying how the proposed reframing builds on and extends prior arguments about informational absence, would strengthen the contribution and avoid the impression that the reframing is entirely novel. Doing so would also highlight more clearly what is distinctive here: the empirical operationalisation of this reframing at scale using platform advertising tools.

Thank you for this suggestion. We have adjusted our manuscript in the following ways.

1. Fully restructuring the front end to create a more coherent narrative, which – as aforementioned – clearly situates our contribution in existing literatures in this space, including changes to our reference list to better reflect the current framing.
2. More clearly emphasizing in our discussion that our findings apply to those with little prior knowledge, who may be more amenable to integrating new information than those with strong (contrary) beliefs or attitudes. There, we also note that our work bridges the literature on “good” information gaps with the research on online interventions (which have mostly focused on minimizing “bad” information).

3. Consider implications of competitive or adversarial advertising environments

A key implication of the proposed reframing is that it relies on market-based advertising infrastructure, which is equally accessible to actors with competing or opposing agendas. While the authors briefly acknowledge that such interventions could be weaponized, the manuscript does not fully engage with what this means for real-world deployment.

The present studies demonstrate effectiveness in largely non-confrontational informational contexts, where competing messages are not systematically introduced. Explicitly acknowledging this boundary condition, and briefly discussing how effectiveness might change in competitive or adversarial advertising environments (i.e. its effectiveness is likely to be dramatically reduced), would strengthen the manuscript’s policy relevance and conceptual completeness, without requiring additional analyses.

Thank you for these good points. We have expanded our discussion section to acknowledge that our results may be harder to generalize to cases of competing informational campaigns targeting the same populations; cautioning that effects we observe may be attenuated or destabilized in more competitive or adversarial ad ecologies, particularly when actors with opposing goals use the same infrastructure to target overlapping audiences. There, we also include an optimistic note that our intervention enhanced digital media literacy and the ability to recognize manipulation and misinformation, which – we hope – could increase users’ resilience to such potential adversarial campaigns.

4. Interpret “belief accuracy” with greater precision

Belief accuracy is operationalized through factual endorsement items administered shortly after exposure. While this is reasonable and clearly reported, the manuscript would benefit from more explicitly acknowledging that such measures may reflect short-term information uptake, accessibility or demand characteristics rather than deeper or more durable belief change.

Clarifying this distinction would help prevent overinterpretation and better align the discussion with what the measures can support, particularly given the paper’s emphasis on belief accuracy as a key outcome.

This is a good point. We have added a paragraph to the limitations portion of our discussion (p. 18 ) noting that the limited nature of our belief accuracy outcome measures means we cannot rule out the possibility of short-term information uptake, accessibility or demand characteristics.

5. Clarify distinctions between beliefs, attitudes, and behavioural intentions

The manuscript distinguishes between beliefs (factual accuracy), attitudes (evaluative judgments), and behavioural intentions, but these distinctions remain largely implicit. Some belief items are politically or normatively loaded, and attitudes are measured using similarly brief binary items, which can blur conceptual boundaries.

Similarly, while the authors generally distinguish behavioural intentions from behaviour, some discussion sections gesture toward downstream behavioural implications more strongly than the evidence allows. Tightening language throughout to maintain these distinctions consistently would improve conceptual clarity and interpretive precision.

Thank you for the insightful comments. We integrate this feedback into the discussion section on p. 18. First, we move the paragraph emphasizing the (very key) limitations of relying on binary questions as outcome measure up in that section. Second, we expand this paragraph to note that our attitude and accuracy measures in particular may have some slippage due to the limitations of working with a single binary poll question. Additionally, we add language in the discussion on p. 17-19	, and amend phrasing on p. 17	to emphasize that we only have direct evidence measuring behavioral intentions, not any downstream behavior. We trust that these changes and additions well address your comment.

Minor Comments

1. The limitations section is generally strong; integrating brief acknowledgements of the points above (belief accuracy interpretation and competitive messaging environments) would improve coherence.

Thank you for this positive assessment. As also noted above, we integrate your comments 4 and 5 into our limitations section on p. 18	.

2. The discussion of persistence effects is appropriately cautious; it may be useful to reiterate clearly that longer-term effects are demonstrated for climate change outcomes specifically.

As suggested, we adjust phrasing on p. 18 of the discussion to emphasize that we only report longer term effects for climate change outcomes and cannot be sure that similar longer term effects would emerge for other issue domains.

3. Consider briefly noting that effects may differ for populations with stronger priors or higher issue engagement, to further contextualize generalizability.

Thank you for this suggestion. We address this point in two ways. First, in the new introduction, we emphasize that our interventions specifically target those who are uninformed (and outline the important theoretical and practical reasons to target this group). Second, we strengthen existing language on this point in the limitations section on p. 9 to acknowledge that the effects may be different among different groups given that news non-users are likely more amenable to new information than those with high-issue engagement.

In sum, thank you for your great feedback. We trust that you will see the final manuscript as theoretically stronger and ready for publication in Pols One.

**Reviewer #2:**

In this paper, the authors presented an internal meta-analysis of 48 social media field experiments designed to promote belief accuracy and change attitudes that may be downstream of misinformation. The authors find that most of their experiments work in increasing belief accuracy and changing public opinion among their samples (people disproportionately unlikely to consume political/news media). Positive effects seem to exist regardless of the intervention type, and the effects of these interventions are highly durable.

I am largely satisfied with the paper as it is. It details an impressive array of experiments. It provides a useful and citable set of evidence that not only are the positive findings for many misinformation interventions credible, but scalable. I really only have one suggestion for the manuscript, which is generally highlighting what the contribution of the interventions are in the front end. In a first read, it sounded like an alternative intervention was being proposed, but in reality, it sounds like the gap this research fills is a) how to scale up that interventions and b) to see if, upon scaling it up, the interventions work in the field like they do in the lab/surveys. Front-loading this would be very useful for the manuscript's visibility. Otherwise, I think this paper is in excellent shape and doesn't really need further changes.

First, thank you for the positive words on our manuscript. Second, we greatly appreciate your important suggestion and agree fully. In response, we have substantially rewritten and restructured the introduction to foreground our unique focus on providing and testing a scalable intervention to introduce factual and verified public affairs information in the social media feeds of news non-users. The revised introduction also notes that the many contemporary challenges which are often attributed to “bad” information online (e.g. misinformative, biased, manipulative) are (as if not more likely) due to the absence of “good” public affairs information in many people’s media diets (this change also addresses a comment from R1). Accordingly, our approach is presented and described as a scalable intervention to address this problem. In addition, on p. 19, we strengthen language in the discussion to note that a major contribution of our project is connecting contemporary work on the absence of “good” public affairs information being a potentially greater democratic problem than ‘bad information’ to research on prosocial interventions.

We believe this change also improves the manuscript’s visibility and makes its distinctive value easier to grasp quickly. Thank you again for your positive assessment of our article!
