# Supplementary material for: Ad-based social media interventions increase belief accuracy and generate pro-social opinions among non-news readers
Source: PLoS One. 2026 Jun 29;21(6):e0352588. doi: 10.1371/journal.pone.0352588 (PMC13313353; doi:10.1371/journal.pone.0352588)
Supplement: Peer review history

# Peer review: Decision Letter 0

**DOI:** 10.1371/journal.pone.0352588.r001

**Authors:** Carlos Carrasco-Farré

2 Apr 2026

PONE-D-25-67892

Ad-Based Social Media Interventions Increase Belief Accuracy and Generate Pro-Social Opinions Among Non-News Readers

PLOS One

Dear Dr. WOJCIESZAK,

Thank you for submitting your manuscript to PLOS ONE. After careful consideration, we feel that it has merit but does not fully meet PLOS ONE’s publication criteria as it currently stands. Therefore, we invite you to submit a revised version of the manuscript that addresses the points raised during the review process.

==============================

I have now received two reviews. Both reviewers were very positive about the paper’s overall quality, and I share their view that this is an ambitious, carefully executed, and potentially highly useful contribution. At the same time, some of the reviewers and I converged on a similar point: the main remaining issues concern framing, positioning, and interpretive precision, rather than the underlying analyses. In other words, the reviewers found the empirical core of the paper strong, but they felt the manuscript would benefit from a clearer articulation of exactly what its contribution is.

Most importantly, the reviewers encourage you to front-load the paper’s central contribution more explicitly. As they read it, the manuscript’s main value is not simply to show that short factual ads can persuade people online, since persuasive effects of media interventions are already well established. Rather, the more distinctive contribution is that the paper reframes a problem often discussed as “misinformation” into one of informational absence among news non-users, and then demonstrates that this reframing can be operationalized at scale through existing advertising infrastructure. Making this point more clearly in the introduction and discussion would help align expectations with what the study actually shows and improve the paper’s visibility.

Relatedly, one reviewer asks that this reframing be situated more explicitly within the relevant literature. The manuscript already cites work on news avoidance, limited exposure to verified information, and interventions aimed at misinformation, but the reviewers felt this could be woven into a more coherent intellectual lineage. This would clarify that the paper is not claiming to invent the broader insight from scratch, but rather to extend and empirically implement it in a novel and scalable way.

I would also encourage somewhat more explicit discussion of the paper’s boundary conditions. In particular, because the intervention relies on advertising infrastructure that is also available to actors with competing or adversarial agendas, it would strengthen the manuscript to briefly acknowledge that the present studies occur in relatively non-confrontational informational environments. A short discussion of how effectiveness may differ in more competitive or adversarial advertising ecologies would improve the paper’s policy relevance without requiring new analyses.

A further set of revisions concerns interpretive precision around the outcomes. One reviewer recommends being more careful with the term “belief accuracy,” noting that the current measures may capture short-term information uptake, accessibility, or response tendencies shortly after exposure rather than deeper or more durable belief revision. Along similar lines, it would help to maintain sharper distinctions among beliefs, attitudes, and behavioural intentions, especially where some items may be politically or normatively loaded, and to avoid language that implies demonstrated behavioural change when only intentions were measured.

There are also a few smaller points worth addressing. The reviewers suggest that the limitations section would be strengthened by integrating these interpretive caveats more directly. It may also be useful to clarify that the longer-term persistence evidence is shown most clearly for the climate change outcomes, and to briefly note that effects may differ among audiences with stronger prior attitudes or greater issue engagement.

Overall, however, the tenor of the reviews is highly encouraging. Both reviewers and I see this as a strong manuscript that already makes an important empirical contribution. The requested changes are primarily to help readers understand the paper on its own terms and to ensure that the claims are framed as precisely as the evidence allows.

I would therefore encourage you to revise the manuscript with these points in mind. I believe that a careful revision along these lines would substantially strengthen what is already a very promising paper.

==============================

Please submit your revised manuscript by May 16 2026 11:59PM. If you will need more time than this to complete your revisions, please reply to this message or contact the journal office at plosone@plos.org. When you're ready to submit your revision, log on to https://www.editorialmanager.com/pone/ and select the 'Submissions Needing Revision' folder to locate your manuscript file.

Please include the following items when submitting your revised manuscript:

- A letter that responds to each point raised by the academic editor and reviewer(s). You should upload this letter as a separate file labeled 'Response to Reviewers'.
- A marked-up copy of your manuscript that highlights changes made to the original version. You should upload this as a separate file labeled 'Revised Manuscript with Track Changes'.
- An unmarked version of your revised paper without tracked changes. You should upload this as a separate file labeled 'Manuscript'.

If you would like to make changes to your financial disclosure, please include your updated statement in your cover letter. Guidelines for resubmitting your figure files are available below the reviewer comments at the end of this letter.

If applicable, we recommend that you deposit your laboratory protocols in protocols.io to enhance the reproducibility of your results. Protocols.io assigns your protocol its own identifier (DOI) so that it can be cited independently in the future. For instructions see: https://journals.plos.org/plosone/s/submission-guidelines#loc-laboratory-protocols. Additionally, PLOS ONE offers an option for publishing peer-reviewed Lab Protocol articles, which describe protocols hosted on protocols.io. Read more information on sharing protocols at https://plos.org/protocols?utm_medium=editorial-email&utm_source=authorletters&utm_campaign=protocols.

As the corresponding author, your ORCID iD is verified in the submission system and will appear in the published article. PLOS supports the use of ORCID, and we encourage all coauthors to register for an ORCID iD and use it as well. Please encourage your coauthors to verify their ORCID iD within the submission system before final acceptance, as unverified ORCID iDs will not appear in the published article. *Only*  the individual author can complete the verification step; PLOS staff *cannot*  verify ORCID iDs on behalf of authors.

We look forward to receiving your revised manuscript.

Kind regards,

Carlos Carrasco-Farré

Academic Editor

PLOS One

**Journal Requirements:**

1. When submitting your revision, we need you to address these additional requirements.

Please ensure that your manuscript meets PLOS ONE's style requirements, including those for file naming. The PLOS ONE style templates can be found at

https://journals.plos.org/plosone/s/file?id=wjVg/PLOSOne_formatting_sample_main_body.pdf and

https://journals.plos.org/plosone/s/file?id=ba62/PLOSOne_formatting_sample_title_authors_affiliations.pdf

2. Please provide details regarding participant consent. In the ethics statement in the Methods and online submission information, please ensure that you have specified (a) whether consent was informed and (b) what type you obtained (for instance, written or verbal, and if verbal, how it was documented and witnessed). If your study included minors, state whether you obtained consent from parents or guardians. If the need for consent was waived by the ethics committee, please include this information.

If you are reporting a retrospective study of medical records or archived samples, please ensure that you have discussed whether all data were fully anonymized before you accessed them and/or whether the IRB or ethics committee waived the requirement for informed consent. If patients provided informed written consent to have data from their medical records used in research, please include this information.

3. We note that the grant information you provided in the ‘Funding Information’ and ‘Financial Disclosure’ sections do not match.

When you resubmit, please ensure that you provide the correct grant numbers for the awards you received for your study in the ‘Funding Information’ section.

4. Thank you for stating the following financial disclosure:

The authors also gratefully acknowledge the support of the European Research Council (ERC Consolidator 101126218, NEWSUSE: Incentivizing Citizen Exposure to Quality News Online: Framework and Tools, PI Magdalena Wojcieszak) and of the Center for Excellence in Social Sciences at the University of Warsaw.

Please state what role the funders took in the study.  If the funders had no role, please state: "The funders had no role in study design, data collection and analysis, decision to publish, or preparation of the manuscript."

If this statement is not correct you must amend it as needed.

Please include this amended Role of Funder statement in your cover letter; we will change the online submission form on your behalf.

5. Thank you for stating the following in the Acknowledgments Section of your manuscript:

The authors thank Craig Newmark Philanthropies, Northwest Conservation Fellows, the Stolte Family Foundation, Luminate Group, the Argosy Foundation, and others for their financial support, and Jon Ozaksut from the Yale Program on Climate Change Communication and Dr. John Cook, University of Melbourne for their feedback on the experimental design. The authors also gratefully acknowledge the support of the European Research Council (ERC Consolidator 101126218, NEWSUSE: Incentivizing Citizen Exposure to Quality News Online: Framework and Tools, PI Magdalena Wojcieszak) and of the Center for Excellence in Social Sciences at the University of Warsaw. Any opinions, findings, conclusions, or recommendations expressed in this material are those of the authors and do not necessarily reflect the views of the funders.

We note that you have provided funding information that is not currently declared in your Funding Statement. However, funding information should not appear in the Acknowledgments section or other areas of your manuscript. We will only publish funding information present in the Funding Statement section of the online submission form.

Please remove any funding-related text from the manuscript and let us know how you would like to update your Funding Statement. Currently, your Funding Statement reads as follows:

The authors also gratefully acknowledge the support of the European Research Council (ERC Consolidator 101126218, NEWSUSE: Incentivizing Citizen Exposure to Quality News Online: Framework and Tools, PI Magdalena Wojcieszak) and of the Center for Excellence in Social Sciences at the University of Warsaw.

Please include your amended statements within your cover letter; we will change the online submission form on your behalf.

6. In the online submission form, you indicated that your data will be submitted to a repository upon acceptance.  We strongly recommend all authors deposit their data before acceptance, as the process can be lengthy and hold up publication timelines. Please note that, though access restrictions are acceptable now, your entire minimal  dataset will need to be made freely accessible if your manuscript is accepted for publication. This policy applies to all data except where public deposition would breach compliance with the protocol approved by your research ethics board. If you are unable to adhere to our open data policy, please kindly revise your statement to explain your reasoning and we will seek the editor's input on an exemption.

7. When completing the data availability statement of the submission form, you indicated that you will make your data available on acceptance. We strongly recommend all authors decide on a data sharing plan before acceptance, as the process can be lengthy and hold up publication timelines. Please note that, though access restrictions are acceptable now, your entire data will need to be made freely accessible if your manuscript is accepted for publication. This policy applies to all data except where public deposition would breach compliance with the protocol approved by your research ethics board. If you are unable to adhere to our open data policy, please kindly revise your statement to explain your reasoning and we will seek the editor's input on an exemption. Please be assured that, once you have provided your new statement, the assessment of your exemption will not hold up the peer review process.

8. Your ethics statement should only appear in the Methods section of your manuscript. If your ethics statement is written in any section besides the Methods, please move it to the Methods section and delete it from any other section. Please ensure that your ethics statement is included in your manuscript, as the ethics statement entered into the online submission form will not be published alongside your manuscript.

9. We note that Figures SM1, SM2, SM3 and SM4 in your submission contain copyrighted images. All PLOS content is published under the Creative Commons Attribution License (CC BY 4.0), which means that the manuscript, images, and Supporting Information files will be freely available online, and any third party is permitted to access, download, copy, distribute, and use these materials in any way, even commercially, with proper attribution. For more information, see our copyright guidelines: http://journals.plos.org/plosone/s/licenses-and-copyright.

We require you to either present written permission from the copyright holder to publish these figures specifically under the CC BY 4.0 license, or remove the figures from your submission:

a. You may seek permission from the original copyright holder of Figures S1, S2, S3 and S4 to publish the content specifically under the CC BY 4.0 license.

We recommend that you contact the original copyright holder with the Content Permission Form (http://journals.plos.org/plosone/s/file?id=7c09/content-permission-form.pdf) and the following text:

“I request permission for the open-access journal PLOS ONE to publish XXX under the Creative Commons Attribution License (CCAL) CC BY 4.0 (http://creativecommons.org/licenses/by/4.0/). Please be aware that this license allows unrestricted use and distribution, even commercially, by third parties. Please reply and provide explicit written permission to publish XXX under a CC BY license and complete the attached form.”

Please upload the completed Content Permission Form or other proof of granted permissions as an "Other" file with your submission.

In the figure caption of the copyrighted figure, please include the following text: “Reprinted from [ref] under a CC BY license, with permission from [name of publisher], original copyright [original copyright year].”

b. If you are unable to obtain permission from the original copyright holder to publish these figures under the CC BY 4.0 license or if the copyright holder’s requirements are incompatible with the CC BY 4.0 license, please either i) remove the figure or ii) supply a replacement figure that complies with the CC BY 4.0 license. Please check copyright information on all replacement figures and update the figure caption with source information. If applicable, please specify in the figure caption text when a figure is similar but not identical to the original image and is therefore for illustrative purposes only.

10. We note that Figures SM2B and SM5 includes an image of a participant in the study.

As per the PLOS ONE policy (http://journals.plos.org/plosone/s/submission-guidelines#loc-human-subjects-research) on papers that include identifying, or potentially identifying, information, the individual(s) or parent(s)/guardian(s) must be informed of the terms of the PLOS open-access (CC-BY) license and provide specific permission for publication of these details under the terms of this license. Please download the Consent Form for Publication in a PLOS Journal (http://journals.plos.org/plosone/s/file?id=8ce6/plos-consent-form-english.pdf). The signed consent form should not be submitted with the manuscript, but should be securely filed in the individual's case notes. Please amend the methods section and ethics statement of the manuscript to explicitly state that the patient/participant has provided consent for publication: “The individual in this manuscript has given written informed consent (as outlined in PLOS consent form) to publish these case details”.

If you are unable to obtain consent from the subject of the photograph, you will need to remove the figure and any other textual identifying information or case descriptions for this individual."

11. Please upload a new copy of Figure 1, as the detail is not clear. Please follow the link for more information:  https://journals.plos.org/plosone/s/figures

12. Please include captions for your Supporting Information files at the end of your manuscript, and update any in-text citations to match accordingly. Please see our Supporting Information guidelines for more information: http://journals.plos.org/plosone/s/supporting-information.

13. If the reviewer comments include a recommendation to cite specific previously published works, please review and evaluate these publications to determine whether they are relevant and should be cited. There is no requirement to cite these works unless the editor has indicated otherwise.

14. Please review your reference list to ensure that it is complete and correct. If you have cited papers that have been retracted, please include the rationale for doing so in the manuscript text, or remove these references and replace them with relevant current references. Any changes to the reference list should be mentioned in the rebuttal letter that accompanies your revised manuscript. If you need to cite a retracted article, indicate the article’s retracted status in the References list and also include a citation and full reference for the retraction notice.

[Note: HTML markup is below. Please do not edit.]

Reviewers' comments:

Reviewer's Responses to Questions

**Comments to the Author**

1. Is the manuscript technically sound, and do the data support the conclusions?

The manuscript must describe a technically sound piece of scientific research with data that supports the conclusions. Experiments must have been conducted rigorously, with appropriate controls, replication, and sample sizes. The conclusions must be drawn appropriately based on the data presented.

Reviewer #1: Yes

Reviewer #2: Yes

**********

2. Has the statistical analysis been performed appropriately and rigorously?

Reviewer #1: Yes

Reviewer #2: Yes

**********

3. Have the authors made all data underlying the findings in their manuscript fully available?

The PLOS Data policy requires authors to make all data underlying the findings described in their manuscript fully available without restriction, with rare exception (please refer to the Data Availability Statement in the manuscript PDF file). The data should be provided as part of the manuscript or its supporting information, or deposited to a public repository. For example, in addition to summary statistics, the data points behind means, medians and variance measures should be available. If there are restrictions on publicly sharing data—e.g. participant privacy or use of data from a third party—those must be specified.

Reviewer #1: Yes

Reviewer #2: Yes

**********

4. Is the manuscript presented in an intelligible fashion and written in standard English?

PLOS ONE does not copyedit accepted manuscripts, so the language in submitted articles must be clear, correct, and unambiguous. Any typographical or grammatical errors should be corrected at revision, so please note any specific errors here.

Reviewer #1: Yes

Reviewer #2: Yes

**********

5. Review Comments to the Author

Please use the space provided to explain your answers to the questions above. You may also include additional comments for the author, including concerns about dual publication, research ethics, or publication ethics. (Please upload your review as an attachment if it exceeds 20,000 characters)

**Reviewer #1:**  Review of “Ad-Based Social Media Interventions Increase Belief Accuracy and Generate Pro-Social Opinions Among Non-News Readers”

Summary

This manuscript reports a large set of field quasi-experiments testing whether short, factual Instagram advertisements can improve belief accuracy, attitudes, and behavioural intentions among news non-users. Using Meta’s advertising infrastructure, the authors demonstrate consistent positive effects on factual endorsement and some attitudinal outcomes across multiple issue domains (climate change, COVID-19 vaccines, election integrity, and media literacy), with no evidence of backfire and some evidence of persistence over time.

The study is ambitious in scale, carefully executed within the constraints of platform-based field experimentation, and transparent about its limitations. The work is well suited to PLOS One’s focus on methodological soundness and contribution through empirical validation. The main issues raised below concern framing, interpretation, and positioning of the contribution rather than the analyses themselves.

Major Comments

1. Clarify the paper’s contribution as reframing rather than persuasion

The manuscript would benefit from more explicitly stating that its primary contribution is not demonstrating that advertising can persuade, an established finding, but rather reframing the broader problem often discussed under the banner of “misinformation” as one of informational absence among news non-users, and empirically showing that this reframing leads to scalable, non-backfiring intervention strategies using existing platform infrastructure.

At present, some parts of the manuscript risk being read as advancing a general claim about persuasive effectiveness of online videos, which invites evaluation criteria (e.g., depth of belief change, behavioural outcomes) that go beyond what the study is designed to address. Clearer signposting of the reframing as the central contribution would better align reader expectations with the paper’s actual strengths.

For example, the authors might consider language along the lines of:

“Rather than testing whether online advertising can persuade, a well-established result, this study demonstrates that reframing the problem as one of informational absence among news non-users yields viable, scalable interventions that improve factual endorsement and attitudes in real-world settings.”

2. Situate the reframing more explicitly within existing literature

Related to the above, the manuscript draws on several existing literatures that have already pointed toward aspects of this reframing, including work on news avoidance and non-use, the informational consequences of limited exposure to verified news, and the unintended effects of debunking- or skepticism-focused interventions. Much of this work is cited, but it is largely treated as background motivation rather than as part of a coherent intellectual lineage.

Making this lineage more explicit, by clarifying how the proposed reframing builds on and extends prior arguments about informational absence, would strengthen the contribution and avoid the impression that the reframing is entirely novel. Doing so would also highlight more clearly what is distinctive here: the empirical operationalisation of this reframing at scale using platform advertising tools.

3. Consider implications of competitive or adversarial advertising environments

A key implication of the proposed reframing is that it relies on market-based advertising infrastructure, which is equally accessible to actors with competing or opposing agendas. While the authors briefly acknowledge that such interventions could be weaponized, the manuscript does not fully engage with what this means for real-world deployment.

The present studies demonstrate effectiveness in largely non-confrontational informational contexts, where competing messages are not systematically introduced. Explicitly acknowledging this boundary condition, and briefly discussing how effectiveness might change in competitive or adversarial advertising environments (i.e. its effectiveness is likely to be dramatically reduced), would strengthen the manuscript’s policy relevance and conceptual completeness, without requiring additional analyses.

4. Interpret “belief accuracy” with greater precision

Belief accuracy is operationalized through factual endorsement items administered shortly after exposure. While this is reasonable and clearly reported, the manuscript would benefit from more explicitly acknowledging that such measures may reflect short-term information uptake, accessibility or demand characteristics rather than deeper or more durable belief change.

Clarifying this distinction would help prevent overinterpretation and better align the discussion with what the measures can support, particularly given the paper’s emphasis on belief accuracy as a key outcome.

5. Clarify distinctions between beliefs, attitudes, and behavioural intentions

The manuscript distinguishes between beliefs (factual accuracy), attitudes (evaluative judgments), and behavioural intentions, but these distinctions remain largely implicit. Some belief items are politically or normatively loaded, and attitudes are measured using similarly brief binary items, which can blur conceptual boundaries.

Similarly, while the authors generally distinguish behavioural intentions from behaviour, some discussion sections gesture toward downstream behavioural implications more strongly than the evidence allows. Tightening language throughout to maintain these distinctions consistently would improve conceptual clarity and interpretive precision.

Minor Comments

1. The limitations section is generally strong; integrating brief acknowledgements of the points above (belief accuracy interpretation and competitive messaging environments) would improve coherence.

2. The discussion of persistence effects is appropriately cautious; it may be useful to reiterate clearly that longer-term effects are demonstrated for climate change outcomes specifically.

3. Consider briefly noting that effects may differ for populations with stronger priors or higher issue engagement, to further contextualize generalizability.

**Reviewer #2:**  In this paper, the authors presented an internal meta-analysis of 48 social media field experiments designed to promote belief accuracy and change attitudes that may be downstream of misinformation. The authors find that most of their experiments work in increasing belief accuracy and changing public opinion among their samples (people disproportionately unlikely to consume political/news media). Positive effects seem to exist regardless of the intervention type, and the effects of these interventions are highly durable.

I am largely satisfied with the paper as it is. It details an impressive array of experiments. It provides a useful and citable set of evidence that not only are the positive findings for many misinformation interventions credible, but scalable. I really only have one suggestion for the manuscript, which is generally highlighting what the contribution of the interventions are in the front end. In a first read, it sounded like an alternative intervention was being proposed, but in reality, it sounds like the gap this research fills is a) how to scale up that interventions and b) to see if, upon scaling it up, the interventions work in the field like they do in the lab/surveys. Front-loading this would be very useful for the manuscript's visibility. Otherwise, I think this paper is in excellent shape and doesn't really need further changes.

**********

6. PLOS authors have the option to publish the peer review history of their article (what does this mean?). If published, this will include your full peer review and any attached files.

If you choose “no”, your identity will remain anonymous but your review may still be made public.

**Do you want your identity to be public for this peer review?**  For information about this choice, including consent withdrawal, please see our Privacy Policy.

Reviewer #1: **Yes:** Piers Howe

Reviewer #2: No

**********

[NOTE: If reviewer comments were submitted as an attachment file, they will be attached to this email and accessible via the submission site. Please log into your account, locate the manuscript record, and check for the action link "View Attachments". If this link does not appear, there are no attachment files.]

To ensure your figures meet our technical requirements, please review our figure guidelines: https://journals.plos.org/plosone/s/figures

You may also use PLOS’s free figure tool, NAAS, to help you prepare publication quality figures: https://journals.plos.org/plosone/s/figures#loc-tools-for-figure-preparation.

NAAS will assess whether your figures meet our technical requirements by comparing each figure against our figure specifications.

# Author comment: Author response to Decision Letter 1

**DOI:** 10.1371/journal.pone.0352588.r002

19 May 2026

Thank you and both reviewers for your careful and constructive evaluations. We are grateful for the positive assessment of the manuscript’s empirical contribution and methodological rigor. We found the comments helpful and have revised the manuscript to respond to all the suggestions. Across the revision, our main goals were to:

(1) clarify and front-load the manuscript’s core contribution, namely to propose and test an intervention that presents more verified and factual information in the social media feeds of users who do not encounter much public affairs information at all;

(2) position that contribution more explicitly within existing literatures on news non-use, informational absence, and misinformation interventions;

(3) revisit and clarify language around behaviors versus behavioral intentions, as well as around belief accuracy and attitudes to more carefully bracket each;

(4) expand the discussion of boundary conditions and limitations.

Below, we respond point by point to each comment from the editor and from each reviewer and the final manuscript highlights the changes in blue.

Additionally, we have included specific point by point responses in an attached document, please see our response to reviewers file for all specific responses to the editor and reviewer comments.

Attachment Submitted filename: RR Memo.docx

# Editor report: Decision Letter 1

**DOI:** 10.1371/journal.pone.0352588.r003

**Authors:** Carlos Carrasco-Farré, Carlos Carrasco-Farré

11 Jun 2026

Ad-Based Social Media Interventions Increase Belief Accuracy and Generate Pro-Social Opinions Among Non-News Readers

PONE-D-25-67892R1

Dear Dr. WOJCIESZAK,

We’re pleased to inform you that your manuscript has been judged scientifically suitable for publication and will be formally accepted for publication once it meets all outstanding technical requirements.

Within one week, you’ll receive an e-mail detailing the required amendments. When these have been addressed, you’ll receive a formal acceptance letter and your manuscript will be scheduled for publication.

An invoice will be generated when your article is formally accepted. Please note, if your institution has a publishing partnership with PLOS and your article meets the relevant criteria, all or part of your publication costs will be covered. Please make sure your user information is up-to-date by logging into Editorial Manager at Editorial Manager® and clicking the ‘Update My Information' link at the top of the page. For questions related to billing, please contact billing support.

If your institution or institutions have a press office, please notify them about your upcoming paper to help maximize its impact. If they’ll be preparing press materials, please inform our press team as soon as possible -- no later than 48 hours after receiving the formal acceptance. Your manuscript will remain under strict press embargo until 2 pm Eastern Time on the date of publication. For more information, please contact onepress@plos.org.

Kind regards,

Carlos Carrasco-Farré

Academic Editor

PLOS One

Additional Editor Comments (optional):

Reviewers' comments:

# Editor report: Acceptance letter

**DOI:** 10.1371/journal.pone.0352588.r004

**Authors:** Carlos Carrasco-Farré, Carlos Carrasco-Farré

PONE-D-25-67892R1

PLOS One

Dear Dr. WOJCIESZAK,

I'm pleased to inform you that your manuscript has been deemed suitable for publication in PLOS One. Congratulations! Your manuscript is now being handed over to our production team.

At this stage, our production department will prepare your paper for publication. This includes ensuring the following:

* All references, tables, and figures are properly cited

* All relevant supporting information is included in the manuscript submission,

* There are no issues that prevent the paper from being properly typeset

You will receive further instructions from the production team, including instructions on how to review your proof when it is ready. Please keep in mind that we are working through a large volume of accepted articles, so please give us a few days to review your paper and let you know the next and final steps.

Lastly, if your institution or institutions have a press office, please let them know about your upcoming paper now to help maximize its impact. If they'll be preparing press materials, please inform our press team within the next 48 hours. Your manuscript will remain under strict press embargo until 2 pm Eastern Time on the date of publication. For more information, please contact onepress@plos.org.

You will receive an invoice from PLOS for your publication fee after your manuscript has reached the completed accept phase. If you receive an email requesting payment before acceptance or for any other service, this may be a phishing scheme. Learn how to identify phishing emails and protect your accounts at https://explore.plos.org/phishing.

If we can help with anything else, please email us at customercare@plos.org.

Thank you for submitting your work to PLOS ONE and supporting open access.

Kind regards,

PLOS ONE Editorial Office Staff

on behalf of

Dr. Carlos Carrasco-Farré

Academic Editor

PLOS One
